# Association of Assisted Reproductive Technology With Offspring Growth and Adiposity From Infancy to Early Adulthood

**DOI:** 10.1001/jamanetworkopen.2022.22106

**Published:** 2022-07-26

**Authors:** Ahmed Elhakeem, Amy E. Taylor, Hazel M. Inskip, Jonathan Huang, Muriel Tafflet, Johan L. Vinther, Federica Asta, Jan S. Erkamp, Luigi Gagliardi, Kathrin Guerlich, Jane Halliday, Margreet W. Harskamp-van Ginkel, Jian-Rong He, Vincent W. V. Jaddoe, Sharon Lewis, Gillian M. Maher, Yannis Manios, Toby Mansell, Fergus P. McCarthy, Sheila W. McDonald, Emanuela Medda, Lorenza Nisticò, Angela Pinot de Moira, Maja Popovic, Irwin K. M. Reiss, Carina Rodrigues, Theodosia Salika, Ash Smith, Maria A. Stazi, Caroline Walker, Muci Wu, Bjørn O. Åsvold, Henrique Barros, Sonia Brescianini, David Burgner, Jerry K. Y. Chan, Marie-Aline Charles, Johan G. Eriksson, Romy Gaillard, Veit Grote, Siri E. Håberg, Barbara Heude, Berthold Koletzko, Susan Morton, George Moschonis, Deirdre Murray, Desmond O’Mahony, Daniela Porta, Xiu Qiu, Lorenzo Richiardi, Franca Rusconi, Richard Saffery, Suzanne C. Tough, Tanja G. M. Vrijkotte, Scott M. Nelson, Anne-Marie Nybo Andersen, Maria C. Magnus, Deborah A. Lawlor, The Assisted Reproductive Technology and Future Health (ART-Health) Cohort Collaboration

**Affiliations:** 1 MRC Integrative Epidemiology Unit at the University of Bristol, Bristol, United Kingdom; 2 Population Health Science, Bristol Medical School, University of Bristol, Bristol, United Kingdom; 3 National Institute for Health Research Bristol Biomedical Research Centre, Bristol, United Kingdom; 4 MRC Lifecourse Epidemiology Centre, University of Southampton, Southampton, United Kingdom; 5 National Institute for Health Research Southampton Biomedical Research Centre, University of Southampton and University Hospital Southampton National Health Service Foundation Trust, Southampton, United Kingdom; 6 Singapore Institute for Clinical Science, Agency for Science, Technology, and Research, Singapore; 7 Academic Clinical Program in Obstetrics and Gynaecology, Duke-NUS Medical School, Singapore; 8 Université de Paris, National Institute for Health and Medical Research, National Research Institute for Agriculture, Food and Environment, Centre for Research in Epidemiology and Statistics, Paris, France; 9 Section of Epidemiology, Department of Public Health, University of Copenhagen, Copenhagen, Denmark; 10 Department of Epidemiology, Lazio Regional Health Service, Rome, Italy; 11 The Generation R Study Group, Erasmus MC, University Medical Center, Rotterdam, the Netherlands; 12 Department of Paediatrics, Erasmus MC, University Medical Center, Rotterdam, the Netherlands; 13 Department of Mother and Child Health, Ospedale Versilia, Viareggio, Azienda Usl Toscana Nord Ovest, Pisa, Italy; 14 Division of Metabolic and Nutritional Medicine, Department of Pediatrics, Dr von Hauner Children’s Hospital, University Hospital, LMU Munich, Munich, Germany; 15 Murdoch Children’s Research Institute, Parkville, Australia; 16 University of Melbourne, Parkville, Australia; 17 Amsterdam University Medical Centers, University of Amsterdam, Department of Public and Occupational Health, Amsterdam Public Health Research Institute, Amsterdam, the Netherlands; 18 Division of Birth Cohort Study, Guangzhou Women and Children’s Medical Center, Guangzhou Medical University, Guangzhou, China; 19 School of Public Health, University College Cork, Cork, Ireland; 20 The Irish Centre for Maternal and Child Health Research, University College Cork, Cork, Ireland; 21 Department of Nutrition and Dietetics, School of Health Science and Education, Harokopio University, Athens, Greece; 22 Institute of Agri-Food and Life Sciences, Hellenic Mediterranean University Research Centre, Heraklion, Greece; 23 Department of Obstetrics and Gynaecology, University College Cork, Cork, Ireland; 24 Department of Paediatrics, Cumming School of Medicine, University of Calgary, Calgary, Canada; 25 Department of Community Health Sciences, Cumming School of Medicine, University of Calgary, Calgary, Canada; 26 Center for Behavioral Sciences and Mental Health, Istituto Superiore di Sanità, Rome, Italy; 27 Cancer Epidemiology Unit, Department of Medical Sciences, University of Turin, Reference Centre for Epidemiology and Cancer Prevention Piemonte, Turin, Italy; 28 Epidemiology Research Unit, Instituto de Saúde Pública, Universidade do Porto, Porto, Portugal; 29 Laboratório para a Investigação Integrativa e Translacional em Saúde Populacional, Porto, Portugal; 30 Centre for Longitudinal Research, He Ara ki Mua, Faculty of Medical and Health Sciences, University of Auckland, Auckland, New Zealand; 31 K.G. Jebsen Center for Genetic Epidemiology, Department of Public Health and Nursing, Faculty of Medicine and Health Sciences, Norwegian University of Science and Technology, Trondheim, Norway; 32 HUNT Research Centre, Department of Public Health and Nursing, Faculty of Medicine and Health Sciences, Norwegian University of Science and Technology, Levanger, Norway; 33 Department of Endocrinology, Clinic of Medicine, St. Olav’s Hospital, Trondheim University Hospital, Trondheim, Norway; 34 Department of Paediatrics, University of Melbourne, Parkville, Australia; 35 Department of Paediatrics, Monash University, Clayton, Australia; 36 Department of Reproductive Medicine, KK Women’s and Children’s Hospital, Singapore; 37 National Institute for Demographic Studies, National Institute for Health and Medical Research, National Blood Service Joint Unit Elfe, Paris, France; 38 Department of Obstetrics and Gynaecology and Human Potential Translational Research Programme, Yong Loo Lin School of Medicine, National University of Singapore, Singapore; 39 Department of General Practice and Primary Health Care, University of Helsinki and Helsinki University Hospital, Helsinki, Finland; 40 Folkhälsan Research Center, Helsinki, Finland; 41 Centre for Fertility and Health, Norwegian Institute of Public Health, Oslo, Norway; 42 Department of Food, Nutrition and Dietetics, School of Allied Health, Human Services and Sport, La Trobe University, Melbourne, Australia; 43 Department of Pediatrics and Child Health, University College Cork, Cork, Ireland; 44 National Longitudinal Study of Children in Ireland, Economic and Social Research Institute, Dublin, Ireland; 45 School of Medicine, University of Glasgow, Glasgow, United Kingdom

## Abstract

**Importance:**

People conceived using assisted reproductive technology (ART) make up an increasing proportion of the world’s population.

**Objective:**

To investigate the association of ART conception with offspring growth and adiposity from infancy to early adulthood in a large multicohort study.

**Design, Setting, and Participants:**

This cohort study used a prespecified coordinated analysis across 26 European, Asia-Pacific, and North American population-based cohort studies that included people born between 1984 and 2018, with mean ages at assessment of growth and adiposity outcomes from 0.6 months to 27.4 years. Data were analyzed between November 2019 and February 2022.

**Exposures:**

Conception by ART (mostly in vitro fertilization, intracytoplasmic sperm injection, and embryo transfer) vs natural conception (NC; without any medically assisted reproduction).

**Main Outcomes and Measures:**

The main outcomes were length / height, weight, and body mass index (BMI; calculated as weight in kilograms divided by height in meters squared). Each cohort was analyzed separately with adjustment for maternal BMI, age, smoking, education, parity, and ethnicity and offspring sex and age. Results were combined in random effects meta-analysis for 13 age groups.

**Results:**

Up to 158 066 offspring (4329 conceived by ART) were included in each age-group meta-analysis, with between 47.6% to 60.6% females in each cohort. Compared with offspring who were NC, offspring conceived via ART were shorter, lighter, and thinner from infancy to early adolescence, with differences largest at the youngest ages and attenuating with older child age. For example, adjusted mean differences in offspring weight were −0.27 (95% CI, −0.39 to −0.16) SD units at age younger than 3 months, −0.16 (95% CI, −0.22 to −0.09) SD units at age 17 to 23 months, −0.07 (95% CI, −0.10 to −0.04) SD units at age 6 to 9 years, and −0.02 (95% CI, −0.15 to 0.12) SD units at age 14 to 17 years. Smaller offspring size was limited to individuals conceived by fresh but not frozen embryo transfer compared with those who were NC (eg, difference in weight at age 4 to 5 years was −0.14 [95% CI, −0.20 to −0.07] SD units for fresh embryo transfer vs NC and 0.00 [95% CI, −0.15 to 0.15] SD units for frozen embryo transfer vs NC). More marked differences were seen for body fat measurements, and there was imprecise evidence that offspring conceived by ART developed greater adiposity by early adulthood (eg, ART vs NC difference in fat mass index at age older than 17 years: 0.23 [95% CI, −0.04 to 0.50] SD units).

**Conclusions and Relevance:**

These findings suggest that people conceiving or conceived by ART can be reassured that differences in early growth and adiposity are small and no longer evident by late adolescence.

## Key Points

**Question:**

Is conception by assisted reproductive technology associated with growth and adiposity from infancy to early adulthood?

**Findings:**

In this cohort study of up to 158 066 infants, children, adolescents and young adults from Europe, Asia-Pacific, and Canada, those conceived using assisted reproductive technology (in vitro fertilization or intracytoplasmic sperm injection, plus embryo transfers) were shorter, lighter, and thinner by most estimates from infancy up to early adolescence compared with their naturally conceived peers; however, the differences were small across all ages and reduced with older age, with 95% CIs sometimes including the null.

**Meaning:**

These findings suggest that parents conceiving or hoping to conceive through assisted reproductive technology and their offspring should be reassured that differences in early life growth and adiposity are small and no longer apparent by late adolescence.

## Teaser

This cohort study investigates the association of conception with assisted reproductive technology with offspring growth and adiposity from infancy to early adulthood across 26 population-based cohorts in Europe, Asia-Pacific, and North America.

## Introduction

Assisted reproductive technology (ART; ie, in vitro handling of both human oocytes and sperm, or of embryos, for the purpose of reproduction^1^), which mainly involves in vitro fertilization (IVF) and intracytoplasmic sperm injection (ICSI), has resulted in more than 8 million births worldwide,^1,2^ and use of ART is expected to continue increasing for several reasons, including increasingly delayed childbearing.^3,4^ Ever since the first ART birth in 1978, research focus has been on improving live-birth rates.^5^ Now that ART is acknowledged as an effective procedure for infertility treatment, attention has shifted toward identifying and reducing any adverse effects of ART on maternal or offspring health. Studies investigating growth-related outcomes have mostly considered perinatal measures, with results showing an increased risk of low birthweight, small-for-gestational-age, and preterm birth in offspring conceived by ART.^6,7,8,9^ Furthermore, studies comparing ART procedures suggest perinatal differences between IVF and ICSI,^10^ and fresh embryo transfers (ET) and frozen-thawed ET (FET).^11,12,13,14^

Besides perinatal outcomes, long-term associations between ART conception and offspring growth and adiposity remain largely unknown, with the few studies that have examined these mostly limited by small sample size, short follow-up, and limited adjustment for confounders or overadjustment for possible mediators.^15,16,17^ A 2021 study that examined trajectories of change in height, weight, and body mass index (BMI; calculated as weight in kilograms divided by height in meters squared) from birth to age 7 years in 81 461 offspring, including 1721 conceived by ART, in the Norwegian Mother, Father and Child Cohort Study (MOBA) found that offspring conceived via ART started smaller and grew faster than those who were NC.^18^ Another considerably smaller Singaporean birth cohort study from 2021 of 1180 offspring, including with 85 conceived by ART, discovered smaller height and lower skinfold thickness at age 6 years in offspring conceived via ART than those who were NC.^19^

Our primary aim was to conduct a multicohort study to provide evidence on the associations of ART conception (compared with NC) with offspring growth and adiposity from infancy to early adulthood. We additionally compared results according to parental subfertility status, in males and females, in ICSI and IVF, and in fresh ET and FET.

## Methods

This multicohort study was carried out within the newly established Assisted Reproductive Technology and Future Health (ART-Health) Cohort Collaboration, following a prespecified analysis plan, which has been published elsewhere.^20^ All cohorts had ethical approval from the relevant local or national ethics committees and all offspring provided informed consent or assent to participate in the respective cohorts and secondary data analyses. Details on ethics approvals and consent are in eMethods in the Supplement. This study is reported following the Strengthening the Reporting of Observational Studies in Epidemiology (STROBE) reporting guideline.

### Cohort Studies

Eligible cohorts were identified from the European Union Child Cohort Network^21,22,23^ and by searching cohort profile papers. We targeted population-based cohorts without selection or oversampling of offspring conceived via ART to reduce potential for selection bias and to ensure identical growth and adiposity assessments for offspring conceived via ART and those who were NC. All cohorts from any geographical region, with birth years from older to more contemporary cohorts, were eligible for inclusion, provided they had data on whether offspring were conceived by ART or NC and 1 or more offspring growth or adiposity outcome measurement assessed from age 1 month (including repeated measurements). A total of 30 cohorts were invited to participate, and 26 were included in this study (eTable 1 in the Supplement). Detailed description of the 26 included cohorts is provided in the eMethods in the Supplement.

### Mode of Conception and Fertility Treatment

Fertility treatment use was defined according to the International Glossary on Infertility and Fertility Care.^1^ Information on mode of conception and fertility treatment was collected using questionnaires or by record linkage (eMethods in the Supplement). This information was used to identify whether offspring were conceived by ART or were NC. *ART* was defined as all interventions that included the in vitro handling of human oocytes and sperm or embryos for reproduction purposes, which mostly included IVF and ICSI (as well as ET) but excluded other medically assisted reproduction, such as assisted insemination.^1^ *NC* was defined as conceiving without fertility treatment or medically assisted reproduction.

We additionally identified whether ART conception involved IVF or ICSI, whether fresh ET or FET was used, and whether NC offspring were born to parents who were fertile, defined as those who became pregnant within 12 months of when they began trying, or parents who were subfertile, defined as those with a time to pregnancy of greater than 12 months after they began trying.^1,24^

### Offspring Growth and Adiposity Outcomes

Primary outcomes for this study were length / height (in centimeters), weight (in kilograms), and BMI. Secondary outcomes were waist circumference (in centimeters), total body fat percentage, and fat mass index (FMI; in kilograms per square meter). Length / height and weight were obtained from research clinics, child health records, and maternal- or self-reports. Waist circumference was mostly measured at research clinics. Body fat percentage was calculated from bioelectrical impedance analysis conducted at research clinics, and FMI was derived as fat mass in kilograms from dual-energy radiograph absorptiometry scans divided by height in meters squared. Details on outcome measurements and ages in each cohort are in the eMethods in the Supplement. Descriptive data on outcomes and ages at outcome assessments are in eTable 2 in the Supplement.

Outcome age groups for this study were determined by available data from each cohort (ie, ages at outcomes assessment). Cohorts were allocated to meta-analysis age groups by mean age at outcome assessment, with the aim of maximizing cohort numbers in each age group meta-analysis. The primary outcomes were allocated to 13 age groups, and secondary outcomes (available in 17 of 26 cohorts) were allocated to 4 age groups. If a cohort had more than 1 outcome assessment in an age group, we selected the one with the biggest sample size.

### Confounders

We used a directed acyclic graph (eFigure 1 in the Supplement) to identify and adjust for confounders, ie, anything that could plausibly cause ART use and influence offspring growth or adiposity.^25,26^ This process identified the following maternal factors as potential confounders: age at pregnancy or birth, socioeconomic status (using education as a proxy), prepregnancy or early-pregnancy BMI, prepregnancy or early-pregnancy smoking, parity, and ethnicity. A total of 19 cohorts were able to adjust for all these confounders, 4 did not adjust for ethnicity but were ethnically homogeneous, 1 did not adjust for parity because it only included nulliparous women, and 2 were unable to adjust for BMI and smoking. Details on the available confounders in each cohort are in eMethods in the Supplement.

### Statistical Analysis

Analyses were performed separately in each cohort, applying identical standardized statistical methods,^20^ and results were combined using meta-analysis. Cohorts were analyzed using R software version 3.6.0 (R Project for Statistical Computing) and Stata software version 14 (StataCorp), and meta-analysis was conducted in R software using the ‘metafor’ package.^27^ In the cohort-specific analyses, we estimated associations of ART conception (vs NC) with offspring outcomes using linear regression adjusted for confounders (plus offspring age and sex). Analysis was performed in offspring with data on mode of conception, at least 1 growth or adiposity outcome, and confounders. To facilitate comparison of results for different outcomes and ages, outcomes were analyzed in age- and sex-cohort–specific SD units (mean [SD], 0 [1]). Cohort results were then combined using random-effects meta-analyses in subgroups defined by mean age at outcome assessment. Variability in the pooled estimates that was due to between-cohort heterogeneity was quantified using the *I*^2^ statistic.^28^ A sensitivity analysis to identify influential cohorts (ie, whose exclusion led to significant change in the meta-analysis model) was conducted by repeating the meta-analyses with each cohort left out in turn.

To separate outcomes associated with ART from any outcomes associated with parental subfertility, we repeated analyses comparing offspring conceived via ART with those who were NC and whose parents were subfertile and separately for offspring who were NC and whose parents were fertile. Differences by sex and ART treatment types were explored by repeating analyses stratified by sex, comparing IVF and ICSI separately with NC, and comparing fresh ET and FET separately with NC. Lastly, we explored if results reflected outcomes associated with multiple births by repeating analysis in singletons and investigated if results were mediated by birth size and pregnancy duration by including extra adjustments (on top of confounders) for birth weight and gestational age. Data were analyzed from November 2019 to February 2022.

## Results

A total of 26 cohorts with participants from Europe (20 cohorts), Australia (2 cohorts), New Zealand (1 cohort), China (1 cohort), Singapore (1 cohort), and Canada (1 cohort) were included in this study (eTable 1 in the Supplement). Most (23 cohorts) were population-based cohorts, 2 were twins-register cohorts, and 1 was a clinical cohort of young adults who had been conceived via ART and NC controls from the same source population. Birth years were from 1984 to 2018, with most (19 cohorts) born after 2002. Mean age at outcome ranged from 0.6 months to 27.4 years. Fifteen cohorts included singletons and multiple births (proportion of multiple births across these ranged from 0.9%-12.9%), 9 cohorts included singletons only, and 2 cohorts included twins only. Between 3 and 16 cohorts were included in each meta-analysis, with numbers of participants in each meta-analysis ranging from 158 066 participants (including 4329 participants conceived via ART) for weight at age 3 to 5 months to 3111 participants (including 151 participants conceived via ART) for FMI at age older than 17 years.

**Figure 1.**
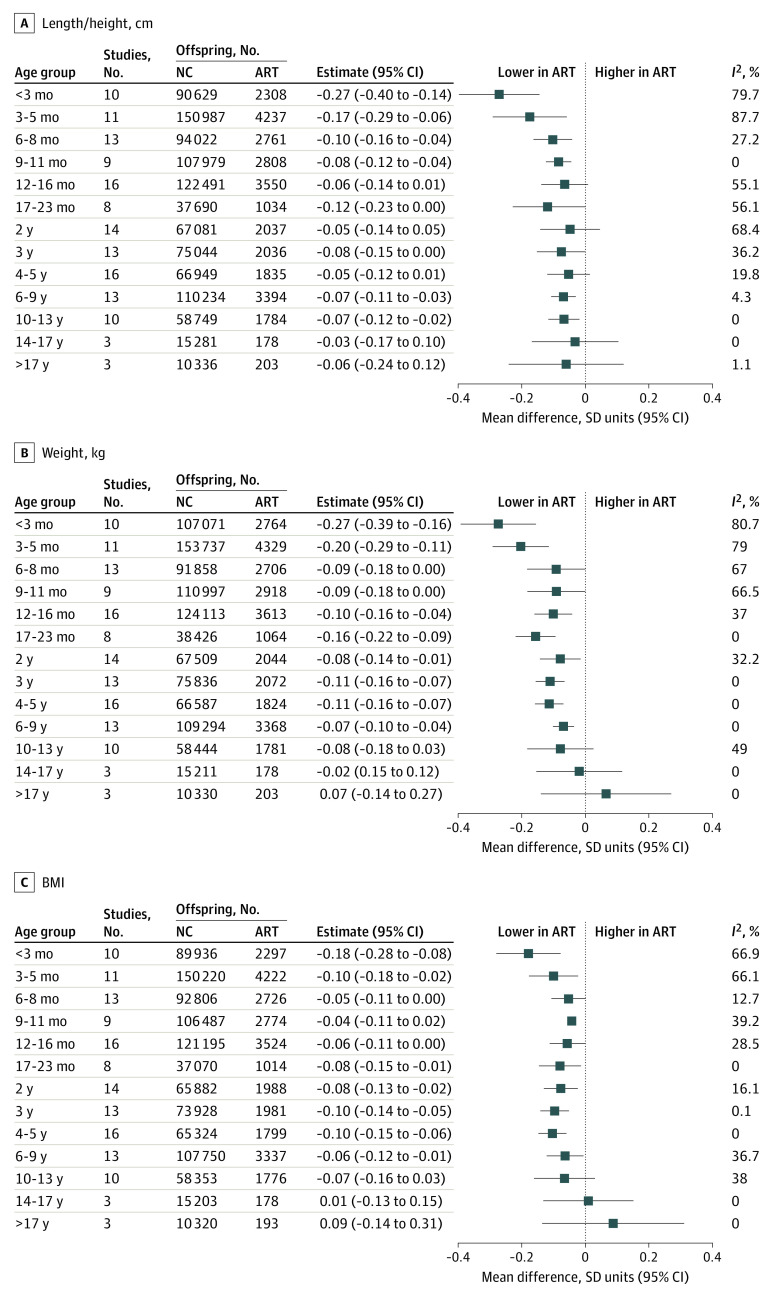
Mean Difference in Length / Height, Weight, and Body Mass Index (BMI) Between Offspring Conceived via Assisted Reproductive Technology (ART) and Those Who Were Naturally Conceived (NC) Cohort-specific results were adjusted for maternal age, parity, BMI (calculated as weight in kilograms divided by height in meters squared), smoking, education, ethnicity or country of birth, plus offspring sex and age. *I*^2^ represents the percentage of total variability that is due to between-cohort heterogeneity. Cohort-specific results are provided in eFigures 2-4 in the Supplement.

Mean length / height was smaller in participants conceived via ART than those conceived via NC in the youngest age groups, although with most older ages, point estimates were close to the null value, which was included in the 95% CIs (Figure 1). The largest differences in length / height were at the youngest ages, and these differences attenuated with older child age. Offspring conceived via ART were more similar in height to offspring who were NC in older adolescence and young adulthood, although estimates were imprecise (Figure 1).

Mean weight was lower in offspring conceived via ART than those who were NC from age younger than 3 months up to age 10 to 13 years, although 95% CIs included the null at ages 6 to 8 months, 9 to 11 months, and 10 to 13 years (Figure 1). For example, adjusted mean differences in offspring weight were −0.27 (95% CI, −0.39 to −0.16) SD units at age younger than 3 months, −0.16 (95% CI, −0.22 to −0.09) SD units at age 17 to 23 months, −0.07 (95% CI, −0.10 to −0.04) SD units at age 6 to 9 years, and −0.02 (95% CI, −0.15 to 0.12) SD units at age 14 to 17 years. Similar to what was observed for length / height, differences were greatest at the youngest ages and smaller at older offspring ages. The difference in mean weight was close to the null in older adolescents, and mean weight in young adults was higher in ART-conceived than NC, but with wide 95% CIs that included the null (Figure 1).

Differences in mean BMI followed a similar pattern to that of weight, with mean BMI lower in offspring conceived via ART than those who were NC up to age 10 to 13 years, with differences being greatest at youngest ages but with wide 95% CIs that included the null value for some results (Figure 1). Similar to what was observed for weight, difference in mean BMI was closest to the null in older adolescents, and mean BMI in young adulthood was higher for participants conceived via ART vs those who were NC, although this was imprecisely estimated with 95% CIs that included the null (Figure 1).

**Figure 2.**
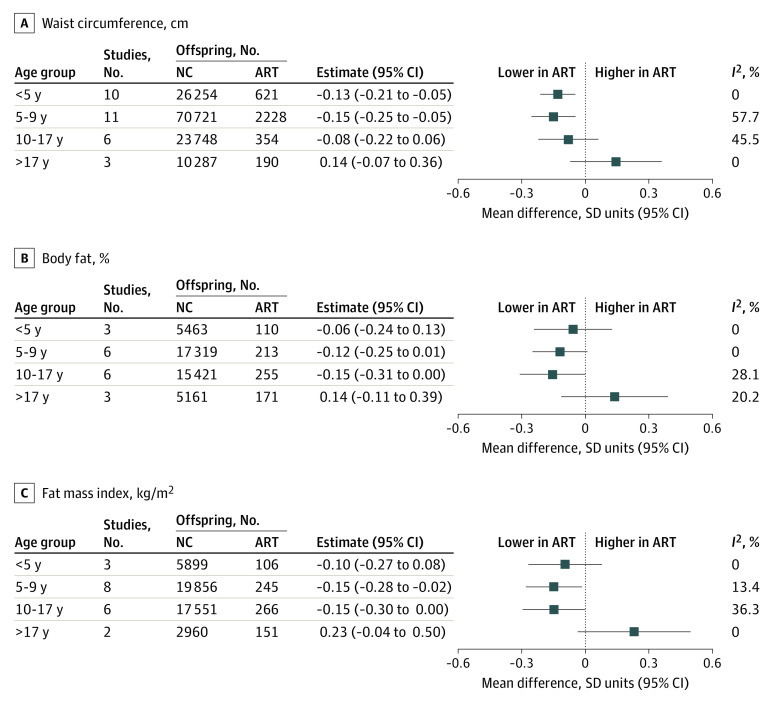
Mean Difference in Waist Circumference, Body Fat Percentage, and Fat Mass Index Between Offspring Conceived via Assisted Reproductive Technology (ART) and Those Who Were Naturally Conceived (NC) Cohort-specific results were adjusted for maternal age, parity, body mass index, smoking, education, ethnicity/country of birth, plus offspring sex and age. *I*^2^ represents the percentage of total variability that is due to between-cohort heterogeneity. Cohort-specific results are provided in eFigures 5-7 in the Supplement.

Results for waist circumference, total body fat percentage, and FMI were like those observed for weight and BMI, with lower mean adiposity measurements during childhood and adolescence in offspring conceived via ART than those who were NC, although with larger differences that were imprecisely estimated and not statistically significant for several time points (Figure 2). Similar to what was observed for weight and BMI, adiposity measures were higher in adulthood for offspring conceived via ART than those who were NC, but with larger mean differences and wider 95% CIs that included the null (eg, ART vs NC difference in fat mass index at age older than 17 years: 0.23 [95% CI, −0.04 to 0.50] SD units) (Figure 2).

Between-cohort heterogeneity was low to moderate for all outcomes at all ages, with a few exceptions. There was substantial between-cohort heterogeneity in results for length / height, weight, and BMI at ages younger than 3 months and 3 to 5 months (Figure 1). Sensitivity analysis showed that results for outcomes at both ages were robust to influential cohorts, although they were attenuated when the MOBA cohort was omitted (eFigure 8 in the Supplement).

**Figure 3.**
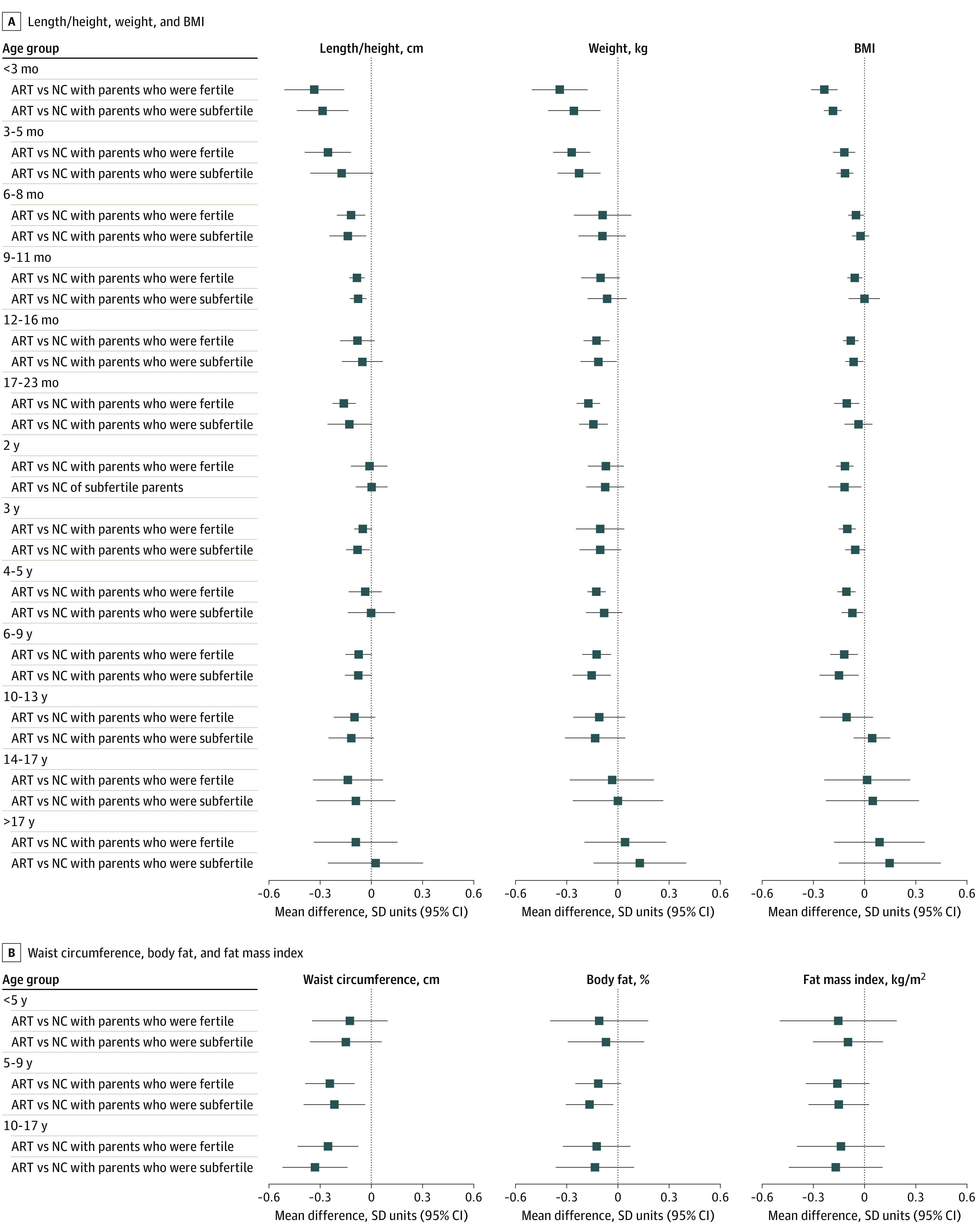
Mean Difference in Growth and Adiposity Outcomes Between Offspring Conceived via Assisted Reproductive Technology (ART) and Those Who Were Naturally Conceived (NC), Separately for Offspring Who Were NC by Parents Who Were Subfertile or Fertile Parents were classified as fertile if time to pregnancy within 12 months from when they began trying; parents were classified as subfertile if time to pregnancy was greater than 12 months. Cohort-specific results were adjusted for maternal age, parity, body mass index (BMI), smoking, education, ethnicity or country of birth, plus offspring sex and age.

**Figure 4.**
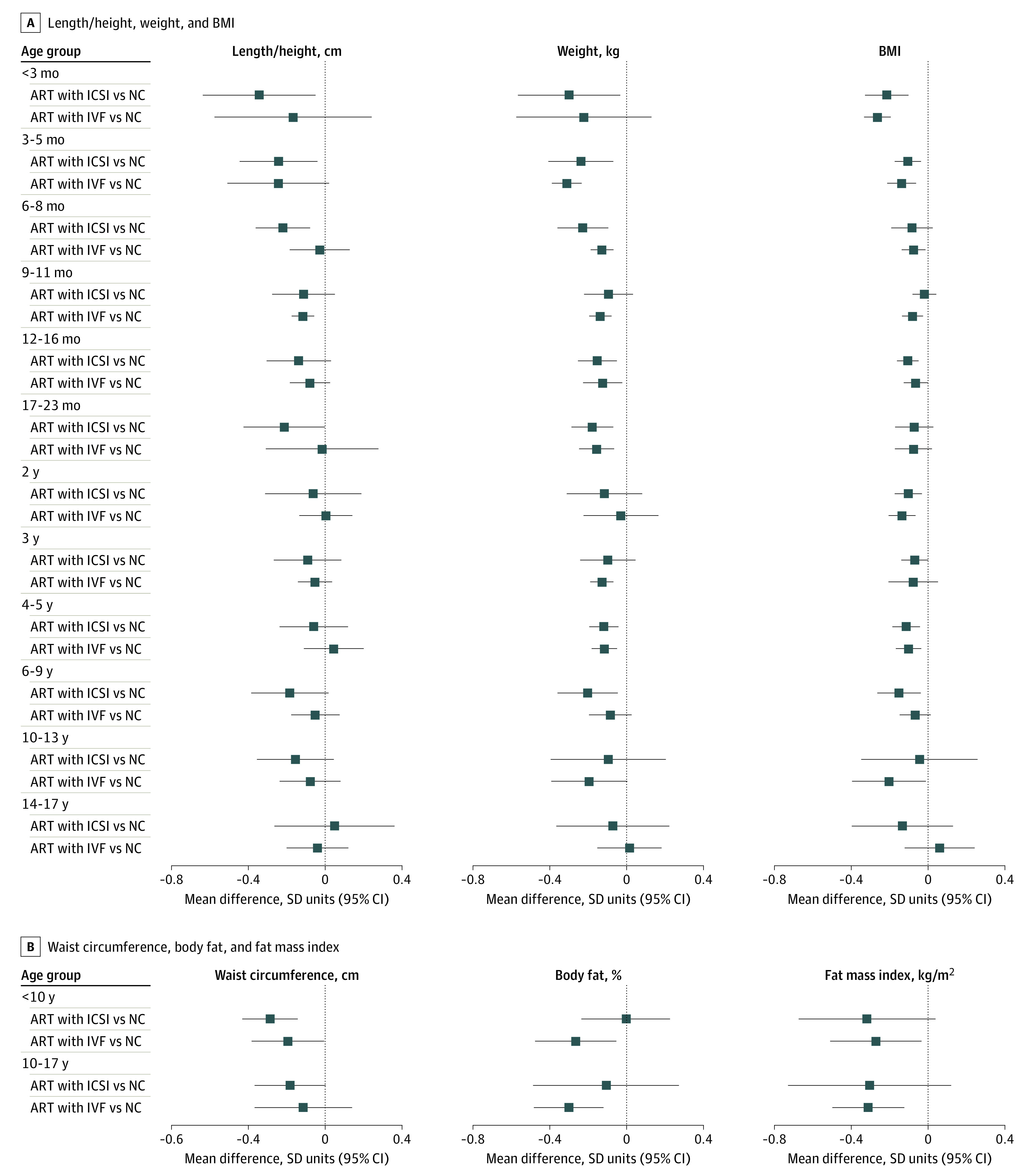
Mean Difference in Growth and Adiposity Outcomes Between Offspring Conceived via Assisted Reproductive Technology (ART) and Those Who Were Naturally Conceived (NC), Separately for Offspring Conceived by Conventional In Vitro Fertilization (IVF) and Intracytoplasmic Sperm Injection (ICSI) Cohort-specific results were adjusted for maternal age, parity, body mass index (BMI; calculated as weight in kilograms divided by height in meters squared), smoking, education, ethnicity or country of birth, plus offspring sex and age. The number of offspring at each age for the primary outcomes (length / height, weight, and BMI) varied from 1517 offspring conceived via conventional IVF, 1382 offspring conceived via ICSI, and 102 386 offspring who were NC for weight at age 3 to 5 months to 105 offspring conceived via conventional IVF, 37 offspring conceived via ICSI, and 11 164 offspring who were NC for BMI at age 14 to 17 years.

**Figure 5.**
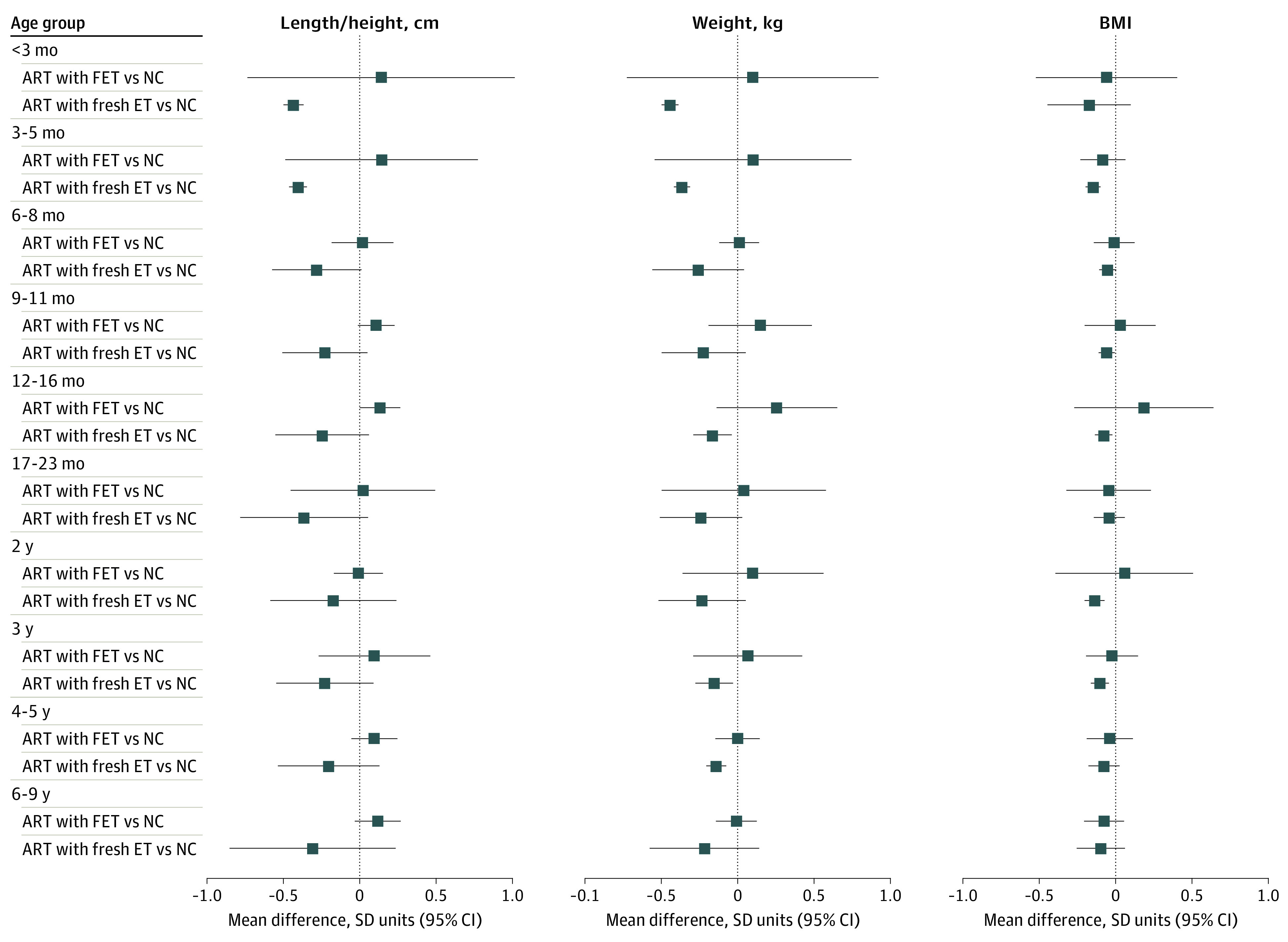
Mean Difference in Length / Height, Weight, and Body Mass Index (BMI) Between Offspring Conceived via Assisted Reproductive Technology (ART) and Those Who Were Naturally Conceived (NC), Separately for Offspring Conceived Using Fresh Embryo Transfer (ET) and Frozen-Thawed Embryo Transfer (FET) Cohort-specific results were adjusted for maternal age, parity, BMI (calculated as weight in kilograms divided by height in meters squared), smoking, education, ethnicity or country of birth, plus offspring sex and age The number of offspring at each age varied from 1904 offspring conceived by fresh ET, 303 offspring conceived by FET, and 78 128 offspring who were NC for weight at age 3 to 5 months to 433 offspring conceived by fresh ET, 84 offspring conceived by FET, and 15 490 offspring who were NC for BMI at age 17 to 23 months.

For some additional analyses, there were too few ART conceptions to include all older age groups. Results were similar when offspring conceived via ART were compared between those who were NC and whose parents were subfertile or fertile (Figure 3), when ICSI and IVF were compared with NC (Figure 4), and in females and males (eFigure 9 in the Supplement). Mean length / height, weight, and BMI were lower in offspring conceived by fresh ET compared with offspring who were NC across all available age groups, ie, from age younger than 3 months to age 6 to 9 years (Figure 5). Conversely, differences were closer to the null for FET compared with NC, although results were imprecise (Figure 5). For example, the differences in weight at age 4 to 5 years was −0.14 (95% CI, −0.20 to −0.07) SD units for fresh ET vs NC and 0.00 (95% CI, −0.15 to 0.15) SD units for FET vs NC. The differences in all growth and adiposity outcomes were only partially attenuated when analyses were restricted to singletons (eFigure 10 in the Supplement), whereas differences between offspring conceived via ART and those who were NC (eFigure 11 in the Supplement), and between offspring conceived via fresh ET and those who were NC (eFigure 12 in the Supplement) were considerably attenuated after further adjustment for birth weight and gestational age, particularly at younger ages.

## Discussion

This cohort study used data from 26 population-based cohort studies to investigate the association of ART conception with offspring growth and adiposity. The large number of offspring and long length of follow-up allowed us to explore findings in subgroups by age from infancy to early adulthood. We found that offspring conceived by ART were shorter, lighter, and thinner by most estimates from infancy to early adolescence than NC offspring. Differences were largest earlier in life but were small in magnitude across all ages and were attenuated as children grew older. There was little evidence that differences were driven by parental subfertility, given similar results when we compared offspring conceived via ART with those who were NC with parents who conceived after 12 months of trying and for whom conception occurred within a shorter period from the start of trying. Offspring conceived from fresh ET were smaller than those who were NC, whereas those conceived via FET were comparable to those who were NC. Results appeared independent of multiple births and were at least partly mediated by birth weight and gestational age, particularly at younger ages.

Our findings are in line with previous studies and reviews of outcomes at birth and in young children.^6,15,16,17^ Although not directly comparable with our study, our finding of smaller differences among older children is consistent with results from a recent study that found more rapid growth from birth to age 3 years in offspring conceived via ART than those who were NC.^18^ That study also examined outcomes at age 17 years in individuals screened for conscription in Norway and found no difference at that age between those who were conceived via ART and those who were NC, which is consistent with our finding of no difference in growth in older adolescence.

Our results for fresh ET and FET are consistent with previous studies showing smaller birth weight in offspring conceived via fresh ET compared with those who were NC, and higher birth weight and large-for-gestational-age in offspring conceived via FET compared with those conceived via fresh ET.^12,13^ Our study also agrees with results from a UK record linkage study that assessed birth size and body size at ages 6 to 8 weeks and 5 years in offspring born between 1997 and 2009, showing that compared with offspring who were NC, offspring born by fresh ET were lighter and those born by FET were heavier at birth and age 6 to 8 weeks, and that all groups had similar weight at 5 years.^16^

The reasons for lower birth weight and higher risk of small-for-gestational-age shown in previous studies^8,15^ and the smaller infant and child size in our study in offspring conceived via ART are not fully understood. The gametic and embryonic manipulations associated with ART may impact embryonic or fetal development in a manner that is reflected in different growth patterns relative to those conceived via NC. Growth differences could also reflect physiological responses to ART-induced lower birth size (and gestational age) and are unlikely to be sex-specific or differ by ART type, given our finding of broadly similar results in males and females and conventional IVF and ICSI. This is supported by our observation that differences attenuate by adjustment for birthweight and gestational age, although this should be interpreted with caution, since assumptions for such analyses and potential for collider bias makes them difficult to interpret.^25,26,28^ Other possible explanations include effects of ART-induced epigenetic alterations,^29,30^ and effects of the ovarian stimulating hormones administered prior to ART.^31^ The different findings for fresh ET and FET may reflect effects of ovarian stimulation on endometrium and corpus luteum when using fresh embryos^32^ or the impact of freezing on embryos.

Ours is the first study to our knowledge to explore long-term associations with waist circumference, body fat percentage, and FMI, with results suggesting individuals conceived via ART had lower central and total adiposity in childhood, and possibly higher levels in adulthood, although with wide 95% CIs that included the null. Our early life results agree with findings from 85 offspring conceived via ART in Singapore showing lower skinfold thickness than offspring who were NC at age 6 years.^19^ Our finding suggestive of higher adiposity in offspring conceived via ART in young adulthood, although with 95% CIs that included the null, is similar in direction to results from a Nordic registry study showing increased obesity risk in young adults who had been conceived via ART.^33^ One possible reason for this result is that the rapid infant growth we observed in offspring conceived via ART continues (at a decelerating rate) for extended time. The direction of our findings was consistent with prior evidence of an association of rapid infant growth with adult overweight and obesity^34^ and with cardiovascular disease risk in later adulthood.^34^

It is important to note that our pooled effect sizes were small across all age groups, including at the youngest ages when they were largest in magnitude. For example, when expressed in its natural units, the largest differences in weight, observed at age younger than 3 months, was 183 (95% CI, 105 to 261) g lower in offspring conceived by ART. Therefore, it is unlikely that these differences will result in any clinically meaningful differences at any age. It is also worth acknowledging that our pooled results represent mean differences in outcomes across all populations from all included cohorts, and there was some evidence of heterogeneity for some outcomes. However, sensitivity analyses indicated results were robust to influential cohorts, and heterogeneity was due to differences in directionally consistent effect sizes.

Strengths of this study include the large sample size and inclusion of cohorts from different geographic regions, which should make our findings generalizable to more populations. The large numbers allowed an assessment of heterogeneity in the main results and explorations of potential roles of subfertility, different ART treatments, multiple births, and indirect effects through prematurity. Use of birth cohorts with comparison with children who were NC from the same underlying population as those conceived by ART and with identical baseline data collection, follow-up periods, and assessments is another important strength. Many previous studies have compared clinical ART cohorts with a comparator group selected at the time of outcome assessment, thus lacking early data on potential confounders, and these were often selected from relatives or friends of the individuals undergoing ART, which may introduce a selection bias.^17^ Record linkage studies can avoid this selection bias but are limited in the extent to which they can adjust for confounding or explore the role of subfertility. Our 2-stage meta-analysis approach, in which all cohorts had harmonized exposures and outcomes and applied identical analyses, helps avoid ethicolegal challenges in sharing participant-level data across borders by only sharing cohort-level summary results (when sharing participant-level data was difficult).

### Limitations

This study has some limitations, including low precision or power at older ages, which highlights the importance of measuring outcomes in adult life. Individuals with outcomes at older ages were exposed to ART some decades ago, using treatments and embryo culture techniques that are less relevant to contemporary practices, thus making it difficult to know the extent to which findings would generalize to more recently born cohorts. Therefore, there is a need to promote collection of data on mode of conception from birth cohorts and to ensure that individuals conceived by ART are included so that future analyses can continually add new cohorts to examine changes in associations by birth years and age. Our analysis was restricted to individuals with complete data on mode of conception, outcomes, and confounders which may have reduced precision of estimates and introduced bias due to missing data. Residual confounding by unmeasured factors (eg, paternal health) is possible and might influence our findings. We did not explore possible mediation by multiple births because more than half of the cohorts only included singleton births, meaning we would have limited power to explore this. Instead, we compared the pooled results in singleton-only cohorts with our main results (ie, across all cohorts).

## Conclusions

In this cohort study, we found that offspring conceived via ART were smaller and had lower adiposity by most estimates than those who were NC during early life, with associations reduced to null with older child age, with some imprecise evidence for higher adiposity by early adulthood with ART conception. Overall, our findings are reassuring since differences in early growth were small, although there is a need for additional follow-up and studies with larger numbers into older ages to investigate the possibility of greater adiposity in adulthood.

## Notes

Supplement.
eMethods.

eReferences.
**eTable 1.** Overview of Participating Cohorts **eTable 2.** Descriptive Data on Participant Numbers and Outcomes in Each Included Cohort **eFigure 1.** Directed Acyclic Graph Used to Identify Potential Confounders **eFigure 2.** Cohort-Specific Mean Differences in Length / Height Between Offspring Conceived via ART and Those Who Were NC **eFigure 3.** Cohort-Specific Mean Differences in Weight Between Offspring Conceived via ART and Those Who Were NC **eFigure 4.** Cohort-Specific Mean Differences in Body Mass Index Between Offspring Conceived via ART and Those Who Were NC **eFigure 5.** Cohort-Specific Mean Differences in Waist Circumference Between Offspring Conceived via ART and Those Who Were NC **eFigure 6.** Cohort-Specific Mean Differences in Body Fat Percentage Between Offspring Conceived via ART and Those Who Were NC **eFigure 7.** Cohort-Specific Mean Differences in Fat Mass Index Between Offspring Conceived via ART and Those Who Were NC **eFigure 8.** Mean Difference In Length / Height, Weight, and Body Mass Index at Ages Younger Than 3 Months and 3 to 5 Months Between Offspring Conceived via ART and Those Who Were NC, After Leaving Each Cohort Study Out of Meta-Analysis **eFigure 9.** Mean Difference in Growth and Adiposity Outcomes Between Offspring Conceived via ART and Those Who Were NC, Stratified by Sex **eFigure 10.** Mean Difference in Growth and Adiposity Outcomes Between Offspring Conceived via ART and Those Who Were NC, Comparing Results in all Participants With Singleton Births Only **eFigure 11.** Mean Difference in Growth and Adiposity Outcomes Between Offspring Conceived via ART and Those Who Were NC, After Further Adjustment for Birth Weight and Gestational Age **eFigure 12.** Mean Difference in Length / Height, Weight, and Body Mass Index Between Offspring Conceived via ART and Those Who Were NC, Separately for Fresh and Frozen-Thawed Embryo Transfer, After Further Adjustment for Birth Weight and Gestational Age **eAppendix.** Cohort-Specific Acknowledgements and Funding
