# Supplementary material for: Association of Assisted Reproductive Technology With Offspring Growth and Adiposity From Infancy to Early Adulthood
Source: JAMA Netw Open. 2022 Jul 26;5(7):e2222106. doi: 10.1001/jamanetworkopen.2022.22106 (PMC9327583; doi:10.1001/jamanetworkopen.2022.22106)
Supplement: Supplement. — eMethods. eReferences. eTable 1. Overview of Participating Cohorts eTable 2. Descriptive Data on Participant Numbers and Outcomes in Each Included Cohort eFigure 1. Directed Acyclic Graph Used to Identify Potential Confounders eFigure 2. Cohort-Specific Mean Differences in Length / Height Between Offspring Conceived via ART and Those Who Were NC eFigure 3. Cohort-Specific Mean Differences in Weight Between Offspring Conceived via ART and Those Who Were NC eFigure 4. Cohort-Specific Mean Differences in Body Mass Index Between Offspring Conceived via ART and Those Who Were NC eFigure 5. Cohort-Specific Mean Differences in Waist Circumference Between Offspring Conceived via ART and Those Who Were NC eFigure 6. Cohort-Specific Mean Differences in Body Fat Percentage Between Offspring Conceived via ART and Those Who Were NC eFigure 7. Cohort-Specific Mean Differences in Fat Mass Index Between Offspring Conceived via ART and Those Who Were NC eFigure 8. Mean Difference In Length / Height, Weight, and Body Mass Index at Ages Younger Than 3 Months and 3 to 5 Months Between Offspring Conceived via ART and Those Who Were NC, After Leaving Each Cohort Study Out of Meta-Analysis eFigure 9. Mean Difference in Growth and Adiposity Outcomes Between Offspring Conceived via ART and Those Who Were NC, Stratified by Sex eFigure 10. Mean Difference in Growth and Adiposity Outcomes Between Offspring Conceived via ART and Those Who Were NC, Comparing Results in all Participants With Singleton Births Only eFigure 11. Mean Difference in Growth and Adiposity Outcomes Between Offspring Conceived via ART and Those Who Were NC, After Further Adjustment for Birth Weight and Gestational Age eFigure 12. Mean Difference in Length / Height, Weight, and Body Mass Index Between Offspring Conceived via ART and Those Who Were NC, Separately for Fresh and Frozen-Thawed Embryo Transfer, After Further Adjustment for Birth Weight and Gestational Age eAppendix. Cohort-Specific Acknowledgements and Funding [file jamanetwopen-e2222106-s001.pdf]

Supplemental Online Content

Elhakeem A, Taylor AE, Inskip HM, et al; Assisted Reproductive Technology and Future Health (ART-Health) Cohort Collaboration. Association of assisted reproductive technology with offspring growth and adiposity from infancy to early adulthood. *JAMA Netw Open*. 2022;5(7):e2222106. doi:10.1001/jamanetworkopen.2022.22106

eMethods.
eReferences.
eTable 1. Overview of Participating Cohorts
eTable 2. Descriptive Data on Participant Numbers and Outcomes in Each Included Cohort
eFigure 1. Directed Acyclic Graph Used to Identify Potential Confounders
eFigure 2. Cohort-Specific Mean Differences in Length / Height Between Offspring Conceived via ART and Those Who Were NC
eFigure 3. Cohort-Specific Mean Differences in Weight Between Offspring Conceived via ART and Those Who Were NC
eFigure 4. Cohort-Specific Mean Differences in Body Mass Index Between Offspring Conceived via ART and Those Who Were NC
eFigure 5. Cohort-Specific Mean Differences in Waist Circumference Between Offspring Conceived via ART and Those Who Were NC
eFigure 6. Cohort-Specific Mean Differences in Body Fat Percentage Between Offspring Conceived via ART and Those Who Were NC
eFigure 7. Cohort-Specific Mean Differences in Fat Mass Index Between Offspring Conceived via ART and Those Who Were NC
eFigure 8. Mean Difference In Length / Height, Weight, and Body Mass Index at Ages Younger Than 3 Months and 3 to 5 Months Between Offspring Conceived via ART and Those Who Were NC, After Leaving Each Cohort Study Out of Meta-Analysis
eFigure 9. Mean Difference in Growth and Adiposity Outcomes Between Offspring Conceived via ART and Those Who Were NC, Stratified by Sex
eFigure 10. Mean Difference in Growth and Adiposity Outcomes Between Offspring Conceived via ART and Those Who Were NC, Comparing Results in all Participants With Singleton Births Only
eFigure 11. Mean Difference in Growth and Adiposity Outcomes Between Offspring Conceived via ART and Those Who Were NC, After Further Adjustment for Birth Weight and Gestational Age
eFigure 12. Mean Difference in Length / Height, Weight, and Body Mass Index Between Offspring Conceived via ART and Those Who Were NC, Separately for Fresh and Frozen-Thawed Embryo Transfer, After Further Adjustment for Birth Weight and Gestational Age
eAppendix. Cohort-Specific Acknowledgements and Funding

This supplemental material has been provided by the authors to give readers additional information about their work.

eMethods.

This section provides a description of the 26 cohort studies, including details of how exposures/outcomes were measured and how ethical approvals and consent were obtained. eTable 1 summarises the characteristics of the cohort studies. eTable 2 provides descriptive data on the numbers included in the analysis including for each timepoint where repeats are available and for each exposure and outcome, as well as the means and standard deviations of each outcome and age at outcome assessment at each assessment wave.

1. All Our Families Study (AOF)

AOF is a community-based longitudinal pregnancy cohort of mother-child dyads investigating maternal, birth and child development outcomes[1]. A total of 3387 pregnant women, residing in Calgary, Canada, enrolled in the study between 2008-2011. Data are collected through self-report questionnaires during pregnancy, post-partum, and post-birth. Mothers have completed eight questionnaires to date spanning pregnancy to 8 years post-birth and provided access to their labour and delivery medical records.

Up to 41 offspring conceived by ART and 1,780 NC offspring were included in this study (including multiple births). AOF contributed results to the main analysis (ART vs. NC) and to additional analysis stratified by sex, sub-fertility, IVF/ICSI, and ET/FET (for FET only), for height, weight, and BMI. Data were available for all study confounders (i.e., maternal age, parity, BMI, smoking, education, ethnicity and offspring sex and age at outcome assessment).

This study was approved by the Child Health Research Office and the Conjoint Health Research Ethics Board of the Faculties of Medicine, Nursing, and Kinesiology, University of Calgary, and the Affiliated Teaching Institutions (Ethics ID 20821 and 22821). Participants provided consent at the time of recruitment and were provided copies of the consent form for their records

2. Amsterdam Born Children and their Development Study (ABCD)

Between January 2003 and March 2004, all pregnant women living in Amsterdam were asked to participate in the ABCD study during their first prenatal visit to an obstetric care provider (general practitioner, midwife, or gynaecologist)[2]. Of the 12,373 women approached, 8,266 women filled out the pregnancy questionnaire (response rate: 67%). Of this group, 7,050 women granted permission for follow-up (85%) and 7,043 women granted permission for perusal of her and her child's medical files (85%). Through a questionnaire, women provided information on time to pregnancy (in months) and mode of conception.

Up to 61 ART-conceived offspring and 4,701 NC offspring were included in this study (singleton births only). ABCD contributed results to the main analysis (ART vs. NC) and to additional analysis stratified by sex, sub-fertility, and IVF/ICSI, for all study outcomes (i.e., height, weight, BMI, waist circumference, body fat % and fat mass index). Data were available for all study confounders (i.e., maternal age, parity, BMI, smoking, education, ethnicity and offspring sex and age at outcome assessment). Confounders were measured by questionnaire (maternal self-report) administered during first trimester of pregnancy.

Approval for the ABCD study was obtained from the Central Committee on Research involving Human Subjects in the Netherlands, the Medical Ethical Committees of the participating hospitals, and from the Registration Committee of the Municipality of Amsterdam. Written informed consent was obtained from all participating mothers.

3. Avon Longitudinal Study of Parents and Children (ALSPAC)

ALSPAC is a prospective birth cohort study that recruited all pregnant women residing within the catchment area of 3 National Health Service authorities in southwest England with an expected date of delivery between April 1991 and December 1992[3-5]. The initial number of pregnancies enrolled is 14,541 (for these at least one questionnaire has been returned or a "Children in Focus" clinic had been attended by 19/07/99). Of these initial pregnancies, there was a total of 14,676 fetuses, resulting in 14,062 live births and 13,988 children who were alive at 1 year of age. Please note that the study website contains details of all the data that is available through a fully searchable data dictionary and variable search tool" and reference the following webpage: http://www.bristol.ac.uk/alspac/researchers/our-data/.

Detailed information has been collected from offspring and their parents using questionnaires, data extraction from medical records, linkage to health records, and clinic assessments up to the last completed contact. Numerous height and weight measures have been obtained from various sources from after birth to age 25 years, including from routine data collected from midwives, health visitors, linkage to child health records, and from ALSPAC research clinic visits. Waist circumference was measured to the nearest mm at research clinics. Body fat % was measured by bio-electrical-impedance (Tanita Body Fat Analyser), and fat mass was measured by whole-body DXA scans (Lunar prodigy).

Up to 56 offspring conceived by ART and 10,380 NC offspring were included in this study (including multiple births). ALSPAC contributed results to the main analysis (ART vs. NC) and to additional analyses stratified by sex, and sub-fertility, for all study outcomes (i.e., height, weight, BMI, waist circumference, body fat % and fat mass index). Data were available for all study confounders (i.e., maternal age, parity, BMI, smoking, education, ethnicity and offspring sex and age at outcome assessment).

Ethical approval for the study was obtained from the ALSPAC Ethics and Law Committee and the Local Research Ethics Committees. Informed consent for the use of data collected via questionnaires and clinics was obtained from participants following the recommendations of the ALSPAC Ethics and Law Committee at the time. At age 18, study children were sent 'fair processing' materials describing ALSPAC's intended use of their health and administrative records and were given clear means to consent or object via a written form. Data were not extracted for participants who objected, or who were not sent fair processing materials. Ethical approval for the study was obtained from the ALSPAC Law and Ethics committee and local research ethics committees (NHS Haydock REC: 10/H1010/70).

4. Babies After SCOPE: Evaluating the Longitudinal Impact on Neurological and Nutritional Endpoints (BASELINE)

The Cork BASELINE Birth Cohort Study [6] is the first Irish prospective birth cohort study and provides detailed information on maternal health, fetal growth, childhood nutrition, growth, and development in the first five years of life. Participants were healthy nulliparous women with singleton pregnancies recruited from the Screening for Pregnancy Endpoints (SCOPE) pregnancy cohort. Detailed information, including information on demographics, lifestyle, obstetric history (including history of fertility and ART) and maternal anthropometric assessment were collected by research midwives at 15 weeks and 20 weeks' gestation using questionnaires and clinical examination. Child anthropometric measures were completed at each study visit (at day 2 and at age 2, 6 and 12 months, and 2 and 5 years) and were conducted according to standard operating procedures. Body composition was measured using air displacement plethysmography with the PEA POD Infant Body Composition System (COSMED USA, Concord, CA).

Up to 20 ART-conceived offspring and 1,031 NC offspring were included in this study (singleton births only). BASELINE contributed results to the main analysis (ART vs. NC) and to additional analysis stratified by sex and sub-fertility, for all study outcomes (i.e., height, weight, BMI, waist circumference, body fat % and fat mass index). Data were available for all study confounders (maternal age, BMI, smoking, education, ethnicity and offspring sex and age at outcome assessment), except for parity as only nulliparous women were included.

Research objectives and measurements in this birth cohort were conducted according to the guidelines laid down in the Declaration of Helsinki and all procedures were approved by the Clinical Research Ethics Committee of the Cork Teaching Hospitals, [ref ECM5(9) 01/07/ 2008]. Families provided written informed consent at 20 weeks' gestation or at birth to participate in BASELINE follow-up.

5. Barwon Infant Study (BIS)

BIS is a prospective pre-birth population-based cohort study (n = 1064 mother–1074 infant pairs [10 sets of twins]) with antenatal recruitment conducted in the Barwon region in Victoria, Australia[7]. Pregnant women were recruited before 28 weeks of gestation between years 2010 and 2013. Detailed questionnaire and clinical data and extensive biospecimens have been collected from multiple time points from pregnancy to 4 years of age. BMI was calculated from height and weight measured by research staff during clinic visits. Body fat percentage was measured using DXA scanning at 4 years of age.

Up to 35 offspring conceived by ART and 673 NC offspring were included in this study. BIS contributed results to the main analysis (ART vs. NC) and to additional sex-stratified analysis, for height, weight, BMI, and body

fat % at the 12-month and 4-year time points. Data were available for all study confounders (i.e., maternal age, parity, BMI, smoking, education, ethnicity and offspring sex and age at outcome assessment).

Ethics approval was obtained from the Barwon Health Human Research Ethics Committee (10/24). All mothers provided written informed consent.

6. Born in Guangzhou Cohort Study (BIGCS)

BIGCS a prospective birth cohort study launched by the Guangzhou Women and Children's Medical Center (GWCMC), China, in 2012[8]. Pregnant women attending their first antenatal care visit at the GWCMC were invited to participate in BIGCS if they were at <20 weeks of gestation, if they intended to deliver at GWCMC and if they intended to stay in Guangzhou for at least 3 years after delivery. Data on the exposures (ART) were collected using a self-reported questionnaire at recruitment. Weight and length/height were measured by trained research assistants. The children were asked to remove their shoes and heavy clothes but to retain a single layer of clothing.

Up to 379 ART-conceived offspring and 9,875 NC offspring were included in this analysis (singleton births only). BIGCS contributed results to the main analysis (ART vs. NC) and to additional analysis stratified by sex and sub-fertility, for, height, weight, and BMI. Data were available for all study confounders (maternal age, BMI, smoking, education, parity and offspring sex and age at outcome assessment), except for ethnicity, which was not inlcuded in the model as all participants were Chinese and about 98% were Han Chinese. Data on potential confounders were collected using self-reported questionnaires.

The study protocol was approved by the institutional ethics committee of the GWCMC. Written informed consent was provided by all participants.

7. Clinical review of the Health of 22–33 years old conceived with and without ART (CHART)

The CHART study[9] is a clinical review of a cohort comprising 547 ART-conceived adults and 549 matched naturally conceived (NC) controls. Recruitment was by letter (postal mailing), with a follow-up letter after 3 weeks and a phone call after a further 3 weeks. Additional attempts at contacting the participants included the use of social media and phone calls to their mothers. Data on clinical and biomarker outcomes were measured including cardiovascular structure and function, auxology, respiratory function, cardiometabolic profile and epigenome-wide DNA methylation analysis. Confounding variables were collected via a questionnaire.

Up to 130 ART-conceived and 73 NC offspring were included in this study. CHART contributed results to the main analysis (ART vs. NC) and to additional analysis stratified by sex and ET/FET, for all study outcomes (i.e., height, weight, BMI, waist circumference, body fat % and fat mass index). Data were available for some study confounders (maternal age, parity, education, and offspring sex and age at outcome assessment), but not for maternal BMI, smoking or ethnicity.

The study was approved by the Royal Children's Hospital Human Research Ethics Committee, and all study participants provided consent to take part in the study.

8. Danish National Birth Cohort (DNBC)

DNBC is a nationwide cohort of pregnant women, recruited from 1996 through 2002 consisting of 100,415 pregnancies[10]. Information on lifestyle and environmental factors potentially associated with offspring health was collected through 4 prenatal and postnatal telephone interviews at target ages gestational weeks 12 and 30 and child ages 6 and 18 months. The parent-child dyads were then invited for follow-up at 7, 11, and 18 years.

Up to 1481 offspring conceived by ART and 45,203 NC offspring were included in this study (including multiple births). DNBC contributed results to the main analysis (ART vs. NC) and to additional sex-stratified analysis, for height, weight, BMI, and waist circumference. Data were available for most study confounders (i.e., maternal age, parity, BMI, smoking, education, and offspring sex and age at outcome assessment), except ethnicity (though >95% were of white ethnicity).

The DNBC complies with the Declaration of Helsinki and was approved by the Danish National Committee on Biomedical Research Ethics. Informed consent was obtained from participants upon enrolment.

9. Etude de cohorte généraliste, menée en France sur les Déterminants pré et post natals précoces du développement psychomoteur et de la santé de l'Enfant (EDEN)

EDEN is a birth cohort study that enrolled 2,002 pregnant women attending their prenatal visit before 24 weeks' gestation at Nancy and Poitiers university hospitals (France) between 2003 and 2006[11]. Detailed information has been collected from parents using questionnaires (including mode of conception and fertility treatment), data extraction from obstetrical file and three clinic assessments up to the last completed contact at age 11 yrs. Height and weight measures were obtained from birth to age 9 years mainly through parental report of measurements performed by health professionals retrieved from the child health booklet and from research clinic visits at 1,3 and 5 years. Waist circumference was measured at the 3- and 5-year research clinic visits. Body fat % was measured by bio-impedance at 5 years.

Up to 22 ART-conceived offspring and 1,326 NC offspring were included in this study. EDEN contributed results to the main analysis (ART vs. NC) and to additional analysis stratified by sex and sub-fertility, for all study outcomes (i.e., height, weight, BMI, waist circumference, body fat % and fat mass index). Data were available for all study confounders (maternal age, BMI, smoking, education, ethnicity, parity and offspring sex and age at outcome assessment).

The study received approval from the ethics committee (CCPPRB) of Kremlin Bicêtre on 12 December 2002 and from CNIL (Commission Nationale Informatique et Liberté), the French data privacy institution. All subjects gave their informed consent for inclusion before they participated in the study. Consent for the child was obtained from both parents after the child's birth.

10. Etude Longitudinale Françcaise depuis l'Enfance (ELFE)

ELFE is a nationwide birth cohort, including 18 329 children born in 2011 in a random sample of 349 maternity units from mainland France[12]. Inclusion criteria were singleton or twins born after 33 weeks' gestation to mothers aged ≥18 years and not planning to move outside of metropolitan France in the next 3 years. Detailed information has been collected from parents using questionnaires ((including mode of conception and fertility treatment), data extraction from obstetrical file, and clinical assessment by general practitioner at age 2. Height and weight measures have been collected through parental report of measurements performed by health professionals retrieved from the child health booklet and from the 2-yr general practionner clinical exam. Data up to 39 months have been used for this analysis.

Up to 309 ART-conceived offspring and 9,632 NC offspring were included in this study (including multiple births). ELFE contributed results to the main analysis (ART vs. NC) and to additional analysis stratified by sex, ICSI/IVF, and sub-fertility, for height, weight, and BMI. Data were available for all study confounders (maternal age, BMI, smoking, education, ethnicity, parity and offspring sex and age at outcome assessment).

Ethical approvals for data collection in maternity units and for each data collection wave during follow-up were obtained from the national advisory committee on information processing in health research (CCTIRS: Comité Consultatif sur le Traitement de l'Information en matière de Recherche dans le domaine de la Santé), the national data protection authority (CNIL: Comission Nationale Informatique et Liberté) and, in case of invasive data collection such as biological sampling, the committee for protection of persons engaged in research (CPP: Comité de Protection des Personnes). The ELFE study was also approved by the national committee for statistical information (CNIS: Conseil National de l'Information Statistique). Informed consent was signed by the parents or the mother alone, with the father being informed of his right to deny consent for participation

11. EU Childhood Obesity Project (CHOP)

The European Childhood Obesity Project (CHOP) was a one-year multicentre double-blind randomized controlled intervention trial including 1678 children (registered at ClinicalTrials.gov: NCT00338689). Healthy singleton term infants born between 1st October 2002 and 31st July 2004 were recruited in five European countries (Belgium, Germany, Italy, Poland, Spain) during their first 8 weeks of life. They were randomized to cow-milk based formula with either higher or lower protein-content. Additionally, a reference group of breastfed children was included. The aim was to test whether feeding infant formula, which differ in their level of milk proteins, can influence infant growth and the risk of later childhood obesity ('early protein hypothesis'). After the intervention, children were prospectively followed up until the age of 11 years. More detailed information on the study design and results can be found elsewhere [13-16]. Data on the exposure (ART) and the

© 2022 Elhakeem A et al. *JAMA Network Open.*

confounders were collected using questionnaires at baseline. Study nurses measured height, weight and waist circumference of the children at various ages.

Up to 20 ART-conceived offspring and 1,479 NC offspring were included in this study (singleton births only). CHOP contributed results to the main analysis (ART vs. NC) only, for height, weight, BMI, and waist circumference. Data were available for all study confounders (maternal age, BMI, smoking, education, ethnicity, parity and offspring sex and age at outcome assessment).

The study was approved by the ethics committees of all study centres. Written informed parental consent was obtained for each infant. All research was conducted in accordance with the Declaration of Helsinki.

12. Gene and Environment: Prospective Study on Infancy in Italy (GASPII)

GASPII is a prospective birth cohort study of 709 children born between June 2003 and October 2004 in 2 maternal units located in Rome, Italy[17].

Up to 8 ART-conceived offspring and 554 NC offspring were included in this study. GASPII contributed results to the main analysis (ART vs. NC) to additional analysis stratified by sub-fertility, for height, weight, BMI, and waist circumference. Data were available for all study confounders (maternal age, BMI, smoking, education, ethnicity, parity and offspring sex and age at outcome assessment).

The protocol of the study has been approved by the Ethics committees of the Università Cattolica del Sacro Cuore, Rome, and all study participants provided consent to take part in the study.

13. Generation R (Gen-R)

Gen-R is a population-based prospective cohort study from fetal life until adulthood[18,19]. In total, 9,778 mothers with a delivery date from April 2002 until January 2006 were enrolled in the study. Response at baseline was 61%. Extensive assessments including physical examinations and DXA measurements are performed in mothers, fathers and their children.

Up to 47 ART-conceived offspring and 4,334 NC offspring were included in this study. Gen-R contributed results to the main analysis (ART vs. NC) to additional analysis stratified by sex, for height, weight, BMI, and fat mass index. Data were available for all study confounders (maternal age, BMI, smoking, education, ethnicity, parity and offspring sex and age at outcome assessment).

The study has been approved by the Medical Ethical Committee of the Erasmus MC, University Medical Center in Rotterdam (MEC-2012-165-NL40020.078.12). Written informed consent was obtained from the parents or legal representatives of the children. Even with consent of the parents, when the child is not willing to participate actively, no measurements are performed.

14. Generation XXI (G21)

Generation XXI (G21) is a prospective population-based birth cohort that recruited pregnant women delivering live-born infants (including multiple births) between April 2005 and August 2006 at all five public maternity units that served the metropolitan area of Porto, Portugal[20]. Overall, 8,647 infants with gestational age above 23 weeks and their mothers (n=8,495) were enrolled (91.4% participation). Subsequent evaluations of the entire cohort took place when children were 4 (n=7,459), 7 (n=6,889), 10 (n=6,397), and 13 years old (n=4,640, interrupted due to the COVID-19 pandemic). The cohort has more than 95% Caucasian participants. Data on demographic and socioeconomic characteristics, lifestyles, obstetric history, and anthropometrics were collected within 72 hours after delivery, in a face-to-face interview conducted by trained interviewers using structured questionnaires. During follow-up, physical examination and multiple questionnaires were performed by trained examiners, according to standard procedures, including weight, height and waist circumference measurements, body fat % and fat mass was measured by bio-impedance.

Up to 92 ART-conceived offspring and 5,746 NC offspring were included in this study (including multiple births). G21 contributed results to the main analysis (ART vs. NC) to additional analysis stratified by sex, sub-fertility, and IVF/ICSI, for all study outcomes (i.e., height, weight, BMI, waist circumference, body fat % and fat mass index). Data were available for all study confounders (maternal age, BMI, smoking, education, maternal country of birth, parity and offspring sex and age at outcome assessment).

The Ethics Committee of Hospital de São João, and of the Institute of Public Health of the University of Porto approved the study protocols. The study complies with the Ethical Principles expressed in the Helsinki Declaration and with the national legislation and was registered with the Portuguese Authority for Data Protection. In all evaluations, participants were informed about the purposes and design of the study, as well as the potential discomfort caused by participation. Signed informed consent was obtained from all parents or legal guardians, and oral assent was obtained from children at each evaluation.

15. Growing Up in Ireland Infant Cohort (GUI)

GUI The Growing Up in Ireland (GUI) study is a nationally representative prospective infant cohort study which recruited a random sample of 11,134 infants born in Ireland from 2007-2008[21]. The children and their families had a baseline face-to-face questionnaire-based interview conducted by trained interviewers in participating households when the infants were approximately nine months old. Mother-infant pairs were subsequently followed-up by home interview when infants were three and five years old. The child's height and weight were measured by a trained interviewer using a validated standard measuring stick (Leicester portable height measure) and a medically approved weighing scale (SECA 835 digital weighing scales). Parity defined as the total number of stillbirths and live births a woman has had was not available, however, we used the number of individuals currently in the study household who were a son/daughter of the mother as a proxy for parity.

Up to 173 ART-conceived offspring and 9,742 NC offspring were included in this study (including multiple births). GUI contributed results to the main analysis (ART vs. NC) to additional analysis stratified by sex, and IVF/ICSI, for height, weight, and BMI. Data were available for all study confounders (maternal age, BMI, smoking, education, ethnicity, parity and offspring sex and age at outcome assessment).

The GUI study received independent ethics approval from a Research Ethics Committee convened by the Department of Health and Children. Written informed consent was obtained from parents or guardians. All methods were performed in accordance with the relevant guidelines and regulations.

16. Growing Up in New Zealand (GUiNZ)

GUiNZ is a prospective birth cohort study that recruited 6,853 children via their pregnant mothers if they had an expected delivery date between 25 April 2009 and 25 March 2010 and were residing within a geographically defined region of New Zealand which was chosen because it could provide a cohort of births that would be representative of all current births in NZ, especially with respect to ethnic and socioeconomic diversity[22]. Birth parameters were retrieved via linkage to routine perinatal records (with maternal consent) and repeated child height and weight measurements were collected as part of field interviews when the children were 2 years and 4 years of age. Anthropometric measurements were undertaken by trained interviewers using a standardised approach used by the NZ Ministry of Health.

Up to 173 ART-conceived offspring and 4,274 NC offspring were included in this study. GUiNZ contributed results to the main analysis (ART vs. NC) to additional analysis stratified by sex, for height, weight, BMI, and waist circumference. Data were available for all study confounders (maternal age, BMI, smoking, education, ethnicity, parity and offspring sex and age at outcome assessment).

Ethical approval for GUiNZ was received from the Ministry of Health Northern Y Regional Ethics Committee (NTY/08/06/055). Written informed consent was obtained from all participating mothers at recruitment and confirmed at each subsequent interview.

17. Growing up in Singapore Towards healthy Outcomes (GUSTO)

GUSTO recruited pregnant women aged 18 years and above, attending their first trimester antenatal dating ultrasound scan clinic at Singapore's two major public maternity units[23]. Women were eligible if 18 years and older, Singaporean citizens or permanent residents, with self-reported homogenous ethnic ancestry (Chinese, Indian, Malay), intended to deliver at the either of the recruitment hospitals and reside in Singapore for the next 5 years. Women greater than 14 weeks of gestation, receiving chemotherapy, psychotropic medications, or having an existing type I diabetes mellitus diagnosis at the time of recruitment were excluded. Women who ultimately did not agree to donate birth tissues (cord, placenta, cord blood) were also excluded. Women were asked to self-report whether the current pregnancy was conceived via IVF and use of assisted reproductive technologies, along with relevant treatment modalities, were confirmed via medical record review by a senior

obstetrician and fertility consultant. Women reporting IVF conception with multiple gestations were further excluded.

Maternal obstetric and medical history including self-reported pre-pregnancy body weight, sociodemographic characteristics, and health behaviors, such as personal and family tobacco smoking, were ascertained by study staff administered standardized questionnaire at recruitment and at a study visit at 26-28 weeks gestation. Mode of delivery, procedures, and complications and birth weight, length, and head circumference were abstracted from delivery record. At all post-delivery visits weight (calibrated Seca 334 or Seca 803 digital scales; Seca, Hamburg, Germany); recumbent crown-to-sole length (up to 24 months; Seca 210 Mobile Measuring Mat) / standing height (beginning at 18 months; Seca 213 Stadiometer); head, mid-upper arm, and abdominal circumferences (inelastic measuring tape); and skinfold (triceps, biceps, subscapular, and suprailiac) thickness (Holtain skinfold calipers; Holtain Ltd., Crymych, UK) were collected by trained study staff in duplicate or triplicate (or 4-5 time for skinfold) and averaged under standardized protocols based on U.S. National Health and Nutrition Examination Survey (NHANES) protocols.

Up to 66 ART-conceived offspring and 935 NC offspring were included in this study (singletons only). GUSTO contributed results to the main analysis (ART vs. NC) to additional analysis stratified by sex, IVF/ICSI, and ET/FET for all study outcomes (i.e., height, weight, BMI, waist circumference, body fat % and fat mass index). Data were available for all study confounders (maternal age, BMI, smoking, education, ethnicity, parity and offspring sex and age at outcome assessment).

18. Healthy Growth Study (HGS)

HGS is a child cohort study started in 2007 that recruited schoolchildren aged 9–13 years, attending primary schools located in municipalities within the counties of Attica, Aitoloakarnania, Thessaloniki and Iraklio, in Greece[24]. Participants underwent a physical examination by two trained members of the research team. The protocol and equipment used were the same in all schools.

Weight was measured to the nearest 10 g using a digital scale (Seca Alpha, model 770; Seca, Hamburg, Germany). Children were weighed without shoes in the minimum clothing possible. Height was measured to the nearest 0·1 cm using a commercial stadiometer (Leicester Height Measure; Invicta Plastics, Oadby, UK) with the child standing barefoot, keeping shoulders in a relaxed position, arms hanging freely and head in the Frankfurt horizontal plane. Waist circumference was measured to the nearest 0·1 cm with the use of a non-elastic tape (Hoechstmass, Sulzbach, Germany) with the child standing, at the end of a gentle expiration, after placing the measuring tape on a horizontal plane around the trunk, at the level of the umbilicus, midway between the lower rib margin and the iliac crest. Bioelectrical impedance analysis (BIA) was used for the assessment of percentage body fat (Akkern BIA 101; Akkern Srl, Florence, Italy). Data on the socio-economic background of the families having at least one child participating in the study were collected from the parents (most preferably from the mother) during scheduled face-to-face interviews at school.

Up to 63 ART-conceived offspring and 2,182 NC offspring were included in this study. HGS contributed results to the main analysis (ART vs. NC) to additional analysis stratified by sex, and IVF/ICSI, for all study outcomes (i.e., height, weight, BMI, waist circumference, body fat % and fat mass index). Data were available for all study confounders (maternal age, BMI, smoking, education, ethnicity, parity and offspring sex and age at outcome assessment).

Approval to conduct the study was granted by the Greek Ministry of National Education and the Ethics Committee of Harokopio University of Athens, and the study was conducted in accordance with the ethical standards specified in the 1964 Declaration of Helsinki. Parents who agreed to the participation of their children in the study had to sign the consent form and provide their contact details.

19. Italian Twins Register (ITR)

ITR[25] is a population-based registry of voluntary twins. Since its inception, 29,000 twins have been enrolled and about 20% of them are minors. The ITR collects information on a large spectrum of phenotypes by either self-reported questionnaires or clinical examinations. In the case of underage twins, the information is reported by the parents who sign an informed consent form.

The ITR offspring were aged between 6 months and 13 years at time of outcome assessments. Due to the wide age range at outcome assessment, ITR was analysed in 7 separate age groups that each included between 32 and

54 ART-conceived offspring (and between 140 and 819 NC offspring (multiple births only). ITR contributed results to the main analysis (ART vs. NC) to additional analysis stratified by sex, and IVF/ICSI (for IVF only), for height, weight, and BMI. Data were available for all study confounders (maternal age, BMI, smoking, education, parity and offspring sex and age at outcome assessment) except for ethnicity.

The study was approved by the ethics committee of Istituto Superiore di Sanità (prot. Number CE-ISS 05-113). All included twins gave their consent to participate in the studies proposed by the ITR research group.

20. Millenium Cohort Study (MCS)

MCS is a nationally representative birth cohort study that followed 19,244 children born in the UK in 2000–2002[26]. Baseline interviews were conducted when the children were approximately nine months old, and follow-up interviews were conducted when the children were around 3, 5, 7, 11, 14 and 17 years old. MCS includes detailed information about the demographic, health and socio-economic characteristics of the respondents and their families. At the baseline interview, the cohort member's mother was asked whether they had used any fertility treatment to conceive.

Up to 30 ART-conceived offspring and 2,153 NC offspring were included in this study (including multiple births). MCS contributed results to the main analysis (ART vs. NC) to additional analysis stratified by sex, sub-fertility, and IVF/ICSI, for all study outcomes (i.e., height, weight, BMI, waist circumference, body fat % and fat mass index). Data were available for all study confounders (maternal age, BMI, smoking, education, ethnicity, parity and offspring sex and age at outcome assessment).

Ethical approval for the Millennium Cohort Study was granted from the multi-centre research ethics committee. Following ethical approval for the study from an NHS Research Ethnics Committee (MREC), informed consent was obtained from parents, as well as from the children themselves as they grew up.

21. MUltiple BIrth Cohort Study (MUBICOS)

MUBICOS [25] has been established within the Italian Twin registry since 2010 but these cohorts do not overlap. About 360 families were enrolled and DNA was taken from parents and twins. Follow-up questionnaires have been administered at 6, 12, 18 and 36 months of age. All height and weight measures are self-reported by parents.

Up to 54 ART-conceived offspring and 101 NC offspring were included in this study (multiple births only). MUBICOS contributed results to the main analysis (ART vs. NC) to additional analysis stratified by sex, and IVF/ICSI (for IVF only), for height, weight, BMI. Data were available for most study confounders (maternal age, BMI, smoking, education, parity and offspring sex and age at outcome assessment), except ethnicity.

The study was approved by the ethics committee of Istituto Superiore di Sanità (prot. Number CE-ISS 09-281). All included twins gave their consent to participate in the studies proposed by the ITR research group.

22. Nascita e INFanzia: gli Effetti dell'Ambiente (NINFEA)

NINFEA study is an Internet-based birth cohort established in 2005 in Italy (http://www.progettoninfea.it)[27]. A baseline questionnaire on general health and exposures before and during pregnancy is completed by mothers at enrolment, which may occur at any time during pregnancy. During the period 2005-2016 around 7,500 mothers were recruited. Further follow-up information was obtained with repeated questionnaires completed 6 and 18 months after delivery and when children turn 4, 7, 10 and 13 years. At each follow-up mothers reported their child current weight and height measurements, and if able to recall or retrieve from baby books or child health records retrospective measurements at pre-defined ages (3 months at 6-month, 12 months at 18-month, and 5 and 6 years at 7-year follow-up). Additional information on whether the measurements were recalled or taken from baby books is available.

Information on exposures was retrieved from the baseline questionnaire completed during pregnancy where mothers reported whether the pregnancy was planned or not, and in the case of affirmative response the following information was collected: i) number of months since she begun trying and became pregnant, ii) if she used any ART treatment, and iii) the type of ART as a checkbox (ovulation induction, intrauterine insemination, gamete intrafallopian transfer, in vitro fertilization, intra-cytoplasmic sperm injection, other technique). Information on the following confounding variables was collected in the baseline and 6-month follow-up

questionnaires: maternal age (continuous), maternal BMI (continuous, derived from maternal pre-pregnancy weight and height reported at enrolment); smoking during pregnancy (yes vs. no, defined as any maternal smoking during pregnancy, independently whether sustained or not), educational level (low — primary school or less, medium—secondary school, and high—university degree), maternal country of birth as a proxy for ethnicity (born in Italy vs. born outside Italy, with more than 95% of mothers born in Italy), and parity (nulliparous vs. multiparous, based on the number of previous livebirths and stillbirths).

Up to 253 ART-conceived offspring and 4,990 NC offspring were included in this study. NINFEA contributed results to the main analysis (ART vs. NC), and to additional analyses stratified by sex, sub-fertility, and IVF/ICSI, for height, weight, and BMI. Data were available for all study confounders (maternal age, BMI, smoking, education, ethnicity, parity and offspring sex and age at outcome assessment).

The Ethical Committee of the San Giovanni Battista Hospital and CTO/CRF/Maria Adelaide Hospital of Turin approved the NINFEA study (approval N. 0048362, and subsequent amendments). Informed consent was obtained from all the participants at enrolment and at each follow-up.

23. Norwegian Mother, Father and Child Cohort Study (MoBa)

MoBa is a nationwide, pregnancy cohort comprising family triads (mother-father-offspring) who are followed longitudinally. All pregnant women in Norway who were able to read Norwegian were eligible to participate. The first child was born in 1999 and the last in 2009[28,29]. Extensive longitudinal data were collected using nine questionnaires: three during pregnancy, and then follow-up questionnaires when the children were 6 months, 18 months, 36 months, 5 years, 7 years and 8 years of age. Data collected include general background and health information, including diet and lifestyle, a semi-quantitative food frequency questionnaire, information on birth and pregnancy outcomes, and on several aspects of child nutrition and development, as well as the physical and mental health of both mother and child. MoBa is linked to the Medical Birth Registry of Norway, which provides standardized information about the health of the mother during pregnancy, other essential medical information related to the pregnancy and birth, and standard post-natal measures of the child. The Medical Birth Registry (MBRN) is a national health registry containing information about all births in Norway.

Up to 2,097 ART-conceived offspring and 77,210 NC offspring were included in this study (multiple birth included). MoBa contributed results to the main analysis (ART vs. NC) to all additional analysis, for height, weight, BMI. Data were available for most study confounders (maternal age, BMI, smoking, education, parity, and offspring sex and age at outcome assessment), but not ethnicity.

The establishment and data collection in MoBa was previously based on a license from the Norwegian Data protection agency and approval from The Regional Committee for Medical Research Ethics, and it is now based on regulations related to the Norwegian Health Registry Act. MoBa is conducted according to the guidelines laid down in the declaration of Helsinki, and written informed consent was obtained from all participants. A detailed protocol of the study including the consent can be found elsewhere (http://www.fhi.no/morogbarn).

24. Piccolipiù

Piccolipiù is a prospective birth cohort study of 3,358 children born in selected maternal units located in five Italian cities (Florence, Rome, Trieste, Turin, and Viareggio) between 2011-2015. Piccolipiù study recruited singleton pregnant women aged at least 18 years old and giving birth in one of the selected maternity units. Mothers were recontacted when the child was 6, 12, 24, 48 months and 6 years old for follow-up questionnaires[30].

Up to 86 ART-conceived offspring and 2,479 NC offspring were included in this study. Piccolipiù contributed results to the main analysis (ART vs. NC) to additional analysis stratified by sex, and sub-fertility, for height, weight, BMI, and waist circumference. Data were available for all study confounders (maternal age, BMI, smoking, education, ethnicity, parity and offspring sex and age at outcome assessment).

The protocol of the study has been approved by the Ethics committees of the Local Health Unit Roma E (management centre), of the Istituto Superiore di Sanità (National Institute of Public Health) and of each local centre. Standard procedures for the protection of confidential individual information were applied according to the Italian law. Consent forms for participation was signed by the mother and also by the father, when both legally responsible for the newborn.

© 2022 Elhakeem A et al. *JAMA Network Open.*

25. Southampton Women's Survey (SWS)

SWS is a population-based prospective birth cohort study of 12 583, initially non-pregnant, women aged 20–34 years, living in the city of Southampton, UK[31]. Assessments of lifestyle, diet and anthropometry were done at study entry in 1998–2002. Women who subsequently became pregnant with singleton pregnancies were followed up during pregnancy; and their offspring have been studied in infancy and childhood. Research nurses collected all anthropometric measurements on offspring and DXA scans were performed at various ages to determine body fat % and fat mass index Information on ART was obtained at the time of the first scan by questioning the mother.

Up to 36 ART-conceived offspring and 2,554 NC offspring were included in this study (singleton births only). SWS contributed results to the main analysis (ART vs. NC) to additional analysis stratified by sex, for all study outcomes (i.e., height, weight, BMI, waist circumference, body fat % and fat mass index). Data were available for all study confounders (maternal age, BMI, smoking, education, ethnicity, parity and offspring sex and age at outcome assessment).

SWS study was conducted according to the guidelines laid down in the Declaration of Helsinki and was approved by the Southampton and South West Hampshire Local Research Ethics Committee (06/Q1702/104). Written informed consent was obtained from all participating women and by a parent or guardian with parental responsibility on behalf of their children.

26. The Trøndelag Health Study (HUNT)

The Trøndelag Health Study (HUNT) is a population-based study where all adult residents of the Nord-Trøndelag region, Norway have been invited to repeated surveys since the 1980s. Since the 1990s, all adolescents (aged 13-19 years) in the region have also been invited (the Young-HUNT Study)[32,33]. The participants have consented to data linkage to health registries, such as the Medical Birth Registry of Norway (MBRN), which includes information on virtually all births in Norway since 1967. In this study, we included participants from the Young-HUNT1 (1995-97), Young-HUNT2 (1999-2000) and Young-HUNT3 (2006-08) surveys, which included clinical measurements of height, weight and waist and hip circumferences. Information on mode of conception was obtained through linkage to information from the MBRN.

Up to 121 ART-conceived offspring and 9,711 NC offspring were included in this study (including multiple births). HUNT contributed results to the main analysis (ART vs. NC) to additional analysis stratified by sex, IVF/ICSI, and ET/FET, for height, weight, BMI, and waist circumference. Data were available for some study confounders (maternal age, parity and offspring sex and age at outcome assessment), but not for maternal BMI, smoking, education, or ethnicity.

The study is approved by the Regional Committee for Medical and Health Research Ethics and by the Norwegian Data Protection Authority, and all study participants gave consent to take part in the study.

eReferences.

1. Tough SC, McDonald SW, Collisson BA, et al. Cohort Profile: The All Our Babies pregnancy cohort (AOB). *Int J Epidemiol* 2017; **46**(5): 1389-90k.
2. van Eijsden M, Vrijkotte TG, Gemke RJ, van der Wal MF. Cohort profile: the Amsterdam Born Children and their Development (ABCD) study. *Int J Epidemiol* 2011; **40**(5): 1176-86.
3. Boyd A, Golding J, Macleod J, et al. Cohort Profile: the 'children of the 90s'--the index offspring of the Avon Longitudinal Study of Parents and Children. *Int J Epidemiol* 2013; **42**(1): 111-27.
4. Fraser A, Macdonald-Wallis C, Tilling K, et al. Cohort Profile: the Avon Longitudinal Study of Parents and Children: ALSPAC mothers cohort. *Int J Epidemiol* 2013; **42**(1): 97-110.
5. Northstone K, Lewcock M, Groom A, et al. The Avon Longitudinal Study of Parents and Children (ALSPAC): an update on the enrolled sample of index children in 2019. *Wellcome open research* 2019; **4**: 51-.
6. O'Donovan SM, Murray DM, Hourihane JO, Kenny LC, Irvine AD, Kiely M. Cohort profile: The Cork BASELINE Birth Cohort Study: Babies after SCOPE: Evaluating the Longitudinal Impact on Neurological and Nutritional Endpoints. *Int J Epidemiol* 2015; **44**(3): 764-75.
7. Vuillermin P, Saffery R, Allen KJ, et al. Cohort Profile: The Barwon Infant Study. *Int J Epidemiol* 2015; **44**(4): 1148-60.
8. Qiu X, Lu J-H, He J-R, et al. The Born in Guangzhou Cohort Study (BIGCS). *European Journal of Epidemiology* 2017; **32**(4): 337-46.
9. Lewis S, Kennedy J, Burgner D, et al. Clinical review of 24-35 year olds conceived with and without in vitro fertilization: study protocol. *Reprod Health* 2017; **14**(1): 117-.
10. Olsen J, Melbye M, Olsen SF, et al. The Danish National Birth Cohort - its background, structure and aim. *Scandinavian Journal of Public Health* 2001; **29**(4): 300-7.
11. Heude B, Forhan A, Slama R, et al. Cohort Profile: The EDEN mother-child cohort on the prenatal and early postnatal determinants of child health and development. *International Journal of Epidemiology* 2015; **45**(2): 353-63.
12. Charles MA, Thierry X, Lanoe J-L, et al. Cohort Profile: The French national cohort of children (ELFE): birth to 5 years. *International Journal of Epidemiology* 2019; **49**(2): 368-9j.
13. Grote V, Theurich M, Luque V, et al. Complementary Feeding, Infant Growth, and Obesity Risk: Timing, Composition, and Mode of Feeding. *Nestle Nutr Inst Workshop Ser* 2018; **89**: 93-103.
14. Weber M, Grote V, Closa-Monasterolo R, et al. Lower protein content in infant formula reduces BMI and obesity risk at school age: follow-up of a randomized trial. *The American journal of clinical nutrition* 2014; **99**(5): 1041-51.
15. Koletzko B, von Kries R, Closa R, et al. Lower protein in infant formula is associated with lower weight up to age 2 y: a randomized clinical trial. *The American journal of clinical nutrition* 2009; **89**(6): 1836-45.
16. Totzauer M, Luque V, Escribano J, et al. Effect of Lower Versus Higher Protein Content in Infant Formula Through the First Year on Body Composition from 1 to 6 Years: Follow-Up of a Randomized Clinical Trial. *Obesity (Silver Spring)* 2018; **26**(7): 1203-10.
17. Porta D, Fantini MP. Prospective cohort studies of newborns in Italy to evaluate the role of environmental and genetic characteristics on common childhood disorders. *Italian Journal of Pediatrics* 2006; **32**: 350-7.
18. Jaddoe VW, Mackenbach JP, Moll HA, et al. The Generation R Study: Design and cohort profile. *Eur J Epidemiol* 2006; **21**(6): 475-84.
19. Kooijman MN, Kruithof CJ, van Duijn CM, et al. The Generation R Study: design and cohort update 2017. *European journal of epidemiology* 2016; **31**(12): 1243-64.
20. Larsen PS, Kamper-Jørgensen M, Adamson A, et al. Pregnancy and birth cohort resources in europe: a large opportunity for aetiological child health research. *Paediatric and perinatal epidemiology* 2013; **27**(4): 393-414.
21. Gallagher AL, Galvin R, Robinson K, Murphy C-A, Conway PF, Perry A. The characteristics, life circumstances and self-concept of 13 year olds with and without disabilities in Ireland: A secondary analysis of the Growing Up in Ireland (GUI) study. *PloS one* 2020; **15**(3): e0229599-e.
22. Morton SMB, Atatoa Carr PE, Grant CC, et al. Cohort Profile: Growing Up in New Zealand. *International Journal of Epidemiology* 2012; **42**(1): 65-75.
23. Soh S-E, Tint MT, Gluckman PD, et al. Cohort Profile: Growing Up in Singapore Towards healthy Outcomes (GUSTO) birth cohort study. *International Journal of Epidemiology* 2013; **43**(5): 1401-9.
24. Moschonis G, Kalliora AC, Costarelli V, et al. Identification of lifestyle patterns associated with obesity and fat mass in children: the Healthy Growth Study. *Public Health Nutr* 2014; **17**(3): 614-24.
25. Brescianini S, Fagnani C, Toccaceli V, et al. An update on the Italian Twin Register: advances in cohort recruitment, project building and network development. *Twin Res Hum Genet* 2013; **16**(1): 190-6.

© 2022 Elhakeem A et al. *JAMA Network Open.*

26. Connelly R, Platt L. Cohort Profile: UK Millennium Cohort Study (MCS). *International Journal of Epidemiology* 2014; **43**(6): 1719-25.
27. Richiardi L, Baussano I, Vizzini L, Douwes J, Pearce N, Merletti F. Feasibility of recruiting a birth cohort through the Internet: the experience of the NINFEA cohort. *Eur J Epidemiol* 2007; **22**(12): 831-7.
28. Magnus P, Birke C, Vejrup K, et al. Cohort Profile Update: The Norwegian Mother and Child Cohort Study (MoBa). *Int J Epidemiol* 2016; **45**(2): 382-8.
29. Magnus P, Irgens LM, Haug K, Nystad W, Skjaerven R, Stoltenberg C. Cohort profile: the Norwegian Mother and Child Cohort Study (MoBa). *Int J Epidemiol* 2006; **35**(5): 1146-50.
30. Farchi S, Forastiere F, Vecchi Brumatti L, et al. Piccolipiù, a multicenter birth cohort in Italy: protocol of the study. *BMC Pediatrics* 2014; **14**(1): 36.
31. Inskip HM, Godfrey KM, Robinson SM, Law CM, Barker DJ, Cooper C. Cohort profile: The Southampton Women's Survey. *Int J Epidemiol* 2006; **35**(1): 42-8.
32. Krokstad S, Langhammer A, Hveem K, et al. Cohort Profile: The HUNT Study, Norway. *International Journal of Epidemiology* 2012; **42**(4): 968-77.
33. Holmen TL, Bratberg G, Krokstad S, et al. Cohort profile of the Young-HUNT Study, Norway: A population-based study of adolescents. *International Journal of Epidemiology* 2013; **43**(2): 536-44.

© 2022 Elhakeem A et al. *JAMA Network Open.*

eTable 1. Overview of Participating Cohorts

Cohort name	Cohort country	Birth years	Analysis sample size (% female)	Number of ART offspring included (%)	Offspring growth/adiposity outcome(s) included in analysis (number of repeat measurements)	Number of meta-analysis age groups contribution (and mean age/range of mean ages if included in >1 age group)
AOF	AU	2008-2011	1,804 (47.8)	41 (2.3)	weight, height, BMI	4 (1.1y to 5.2y)
ABCD	NL	2003-2004	4,510 (55.5)	61 (1.4)	weight, height, BMI, waist, bio, FMI	10 (0.2y to 11.7y)
ALSPAC	UK	1990-1992	8,652 (48.8)	53 (0.6)	weight, height, BMI, waist, bio, FMI	9 (0.1y to 24.5y)
BASELINE	IE	2008-2011	1,051 (48.7)	20 (1.9)	weight, height, BMI, waist, bio, FMI	5 (0.2y to 5.1y)
BIS	AU	2010-2013	708 (47.6)	35 (4.9)	weight, height, BMI, bio	2 (1.1y and 4.2y)
BIGCS	CH	2012-present	10,074 (47.7)	349 (3.5)	weight, height, BMI	4 (0.1y to 2.8y)
CHART	AU	1982-1992	203 (60.6)	130 (64.0)	weight, height, BMI, waist, bio, FMI	1 (27.4y)
DNBC	DN	1996-2003	36,380 (48.6)	1,481 (4.1)	weight, height, BMI, waist	4 (0.4y to 11.3y)
EDEN	FR	2003-2006	1,348 (48.0)	22 (1.6)	weight, height, BMI, waist, bio, FMI	6 (0.3y to 5.7y)
ELFE	FR	2011	9,941 (48.9)	309 (3.1)	weight, height, BMI	5 (0.3y to 3.0y)
CHOP	EU	2002-2004	1,499 (50.0)	20 (1.3)	weight, height, BMI, waist	4 (0.1y to 1.0y)
GASPII	IT	2003-2004	562 (48.8)	8 (1.4)	weight, height, BMI, waist	3 (1.4y to 7.8y)
Gen-R	NL	2002-2006	4,307 (49.6)	51 (1.2)	weight, height, BMI, waist, bio, FMI	6 (0.1y to 9.8y)
G21	PO	2005-2006	4,756 (48.8)	92 (1.9)	weight, height, BMI, waist, bio, FMI	11 (0.2y to 10.2y)
GUI	IE	2011	9,915 (48.8)	173 (1.7)	weight, height, BMI	3 (0.8y to 5.2y)
GUiNZ	NZ	2009-2010	4,447 (48.9)	173 (3.9)	weight, height, BMI, waist	3 (2.0y to 8.6y)
GUSTO	SG	2009-2010	905 (48.3)	64 (7.1)	weight, height, BMI, waist, bio, FMI	10 (0.1y to 6.1y)
HGS	GR	2007-2009	2,245 (50.7)	63 (2.8)	weight, height, BMI, waist, bio, FMI	1 (11.2 years)
ITR	IT	2003-2018	248 (49.2)	54 (21.8)	weight, height, BMI	7 (0.6y to 13.6y)
MCS	UK	2000-2002	2,183 (48.0)	30 (1.4)	weight, height, BMI, waist, bio, FMI	5 (3.1y to 17.2y)
MUBICOS	IT	2009-2015	155 (48.4)	54 (34.8)	weight, height, BMI	2 (1.1y and 3.0y)
NINFEA	IT	2006-2017	5,260 (49.4)	270 (5.1)	weight, height, BMI	8 (0.3y to 10.2y)
MoBa	NO	1998-2008	79,358 (49.1)	2,148 (2.7)	weight, height, BMI	10 (0.1y to 7.1y)
Piccolipiù	IT	2011-2014	2,565 (48.3)	86 (3.4)	weight, height, BMI, waist	7 (0.1y to 4.4y)
SWS	UK	1998-2005	2,589 (48.2)	35 (1.4)	weight, height, BMI, waist, bio, FMI	7 (0.5y to 9.2y)
HUNT	NO	1984-2006	9,832 (49.8)	121 (1.2)	weight, height, BMI, waist	2 (15.0y to 18.1y)
Potentially eligible cohorts not included in analyses						
BiB*	UK	2007-2011	13,740 (49.3)	7	weight, height, BMI	-
Raine*	AU	1989-1991	around 5,000	5	weight, height, BMI, waist, bio, FMI	-
PREDO*	FI	2006-2010	around 2,500	-	weight, height, BMI	-
UBCoS*	SE	-	-	-	-	-

For studies with repeat measures (and multiple outcomes), sample size and number of ART is shown for timepoint with the largest number of ART offspring. Thirty likely eligible cohorts were invited and all, except one, agreed to participate in this study. We had pre-specified that to be included cohorts should have data on at least 10 ART-

conceived infants. Of those that agreed, all, except three, completed their analysis and were included. Two of the three cohorts informed us that they had fewer than 10 ART-conceived infants and the other one did not to respond to repeated requests to complete the analysis. The four excluded cohorts are indicated by *. The BiB (Born in Bradford: https://borninbradford.nhs.uk/) and Raine (The Western Australian Pregnancy Cohort: https://rainestudy.org.au/) studies did not participate because once they checked they reported that they had too few offspring conceived using ART according to our criterial of cohorts having to have at least 10 ART conceived infants. PREDO (Prediction and Prevention of Preeclampsia and Intrauterine Growth Restriction: https://academic.oup.com/ije/article/46/5/1380/2622848) initially agreed to contribute but unfortunately did not respond to subsequent requests to run the analysis and was excluded from the meta-analysis. UBCoS (Uppsala Birth Cohort Multigeneration Study: https://www.chess.su.se/ubcosmg/) did not respond to the initial invitations to participate in this study and was excluded.

© 2022 Elhakeem A et al. *JAMA Network Open.*

eTable 2. Descriptive Data on Participant Numbers and Outcomes in Each Included Cohort

AOF															
**Cardio-metabolic outcome**	**Age at outcome assessment - y**		**Outcome at assessment - kg, cm etc.**		**Numbers included in the analyses: adjusted models**										
	**Mean**	**SD**	**Mean**	**SD**	**ART**	**ART: males**	**ART: females**	**ICSI**	**IVF**	**FET**	**ET**	**NC**	**NC: males**	**NC: females**	**NC: fertile parents**	**NC: sub-fertile parent**
**Weight - kg**	1.1	0.1	9.9	1.6	36	17	19	20	16	x	21	1386	726	660	1257	219
	2.0	0.1	12.5	1.6	41	20	21	21	20	x	27	1406	724	682	1278	213
	3.0	0.2	14.7	1.9	37	17	20	19	18	x	23	1811	941	870	1655	310
	5.2	0.3	19.2	2.9	41	22	19	23	18	x	26	1763	919	844	1621	287
**Height - cm**	1.1	0.1	73.8	9.4	35	17	18	19	16	x	20	1296	676	620	1173	207
	2.0	0.1	86.2	6.8	41	20	21	21	20	x	27	1358	697	661	1235	202
	3.0	0.2	96.0	4.8	37	17	20	19	18	x	23	1780	925	855	1626	305
	5.2	0.3	111.1	7.0	41	22	19	23	18	x	26	1731	898	833	1589	282
**BMI - kg/m2**	1.1	0.1	19.9	14.1	35	17	18	19	16	x	20	1292	674	618	1169	205
	2.0	0.1	17.0	3.5	41	20	21	21	20	x	27	1357	697	660	1234	202
	3.0	0.2	15.9	1.9	37	17	20	19	18	x	23	1774	921	853	1621	304
	5.2	0.3	15.6	3.0	41	22	19	23	18	x	26	1725	896	829	1584	281

ABCD																
**Cardio-metabolic outcome**	**Age at outcome assessment - y**		**Outcome at assessment - kg, cm etc.**		**Numbers included in the analyses: adjusted models**											
	**Mean**	**SD**	**Mean**	**SD**	**ART**	**ART: males**	**ART: females**	**ICSI**	**IVF**	**FET**	**ET**	**NC**	**NC: males**	**NC: females**	**NC: fertile parents**	**NC: sub-fertile parent**
**Weight (kg)**	0.2	0.1	5.7	0.9	61	30	31	22	39	x	x	4449	2203	2246	3270	1179
	0.3	0.6	6.2	2.1	55	25	30	19	36	x	x	4701	2338	2363	3304	1397

© 2022 Elhakeem A et al. *JAMA Network Open.*

	0.6	0.5	8.2	1.7	51	23	28	15	36	x	x	4388	2185	2203	3091	1297
	0.8	0.5	9.0	1.9	49	23	26	15	34	x	x	4319	2156	2163	3029	1290
	1.1	0.7	10.0	2.3	51	23	28	16	35	x	x	4168	2058	2110	2939	1229
	1.5	0.9	11.2	2.9	48	23	25	15	33	x	x	4021	2001	2020	2849	1172
	2.1	1.2	12.7	3.4	47	20	27	14	33	x	x	3874	1929	1945	2744	1130
	5.7	0.5	20.8	3.3	35	17	18	12	23	x	x	3581	1791	1790	2662	919
	10.6	0.4	38.2	8.3	36	19	17	12	24	x	x	2741	1324	1417	1887	854
	11.7	0.4	40.5	7.2	30	13	17	13	17	x	x	2304	1156	1148	1772	532
**Height (cm)**	0.2	0.1	59.3	3.5	60	29	31	21	39	x	x	4328	2152	2176	3190	1138
	0.3	0.6	61.7	7.9	50	22	28	18	32	x	x	4395	2201	2194	3079	1316
	0.6	0.5	69.3	5.8	49	21	28	14	35	x	x	4284	2141	2143	3019	1265
	0.8	0.5	72.4	6.3	46	23	23	14	32	x	x	4229	2118	2111	2961	1268
	1.1	0.7	76.2	8.0	49	22	27	16	33	x	x	4082	2015	2067	2877	1205
	1.5	0.9	81.1	10.0	48	23	25	15	33	x	x	3919	1953	1966	2772	1147
	2.1	1.2	87.1	11.4	45	18	27	12	33	x	x	3813	1900	1913	2706	1107
	5.7	0.5	116.0	5.8	35	16	19	13	22	x	x	3598	1798	1800	2672	926
	10.6	0.4	146.4	7.2	36	19	17	12	24	x	x	2742	1325	1417	1888	854
	11.7	0.4	153.1	7.0	34	14	20	15	19	x	x	2517	1249	1268	1937	580
**BMI (kg/m2)**	0.2	0.1	16.1	1.5	60	29	30	20	39	x	x	4288	2089	2142	3168	1120
	0.3	0.6	16.2	1.5	50	22	28	18	32	x	x	4394	2200	2194	3078	1316
	0.6	0.5	17.1	1.5	49	21	28	14	35	x	x	4273	2136	2137	3014	1259
	0.8	0.5	17.2	1.5	46	23	23	14	32	x	x	4225	2114	2111	2959	1266
	1.1	0.7	17.1	1.4	49	22	27	16	33	x	x	4075	2012	2063	2871	1204
	1.5	0.9	16.9	1.4	48	23	25	15	33	x	x	3912	1949	1963	2768	1144
	2.1	1.2	16.5	1.4	44	18	26	12	32	x	x	3798	1893	1905	2693	1105
	5.7	0.5	15.4	1.5	35	16	19	13	22	x	x	3498	1748	1750	2594	904
	10.6	0.4	17.7	3.0	36	19	17	12	24	x	x	2738	1322	1416	1885	553
	11.7	0.4	17.2	2.2	28	11	17	13	15	x	x	2236	1133	1103	1730	506

© 2022 Elhakeem A et al. *JAMA Network Open.*

**Waist circumference - cm**	5.7	0.5	52.5	3.9	26	14	12	9	17	x	x	2915	1460	1455	2152	763
	11.7	0.4	62.5	6.1	14	5	9	4	10	x	x	983	504	479	770	213
**Body fat %**	5.7	0.5	23.8	6.3	25	13	12	8	17	x	x	2881	1446	1435	2126	755
	11.7	0.4	23.4	5.6	14	5	9	4	10	x	x	967	496	471	756	211
**Fat mass index - kg/m2**	5.7	0.5	3.2	1.3	25	13	12	8	17	x	x	2878	1443	1435	2125	753
	11.7	0.4	4.2	1.6	14	5	9	4	10	x	x	967	496	471	756	211
**ALSPAC**																
**Cardio-metabolic outcome**	**Age at outcome assessment - y**		**Outcome at assessment - kg, cm etc.**		**Numbers included in the analyses: adjusted models**											
	**Mean**	**SD**	**Mean**	**SD**	**ART**	**ART: males**	**ART: females**	**ICSI**	**IVF**	**FET**	**ET**	**NC**	**NC: males**	**NC: females**	**NC: fertile parents**	**NC: sub-fertile parent**
**Weight - kg**	0.1	0.0	5.0	0.7	53	36	17	x	x	x	x	8599	4399	4200	x	x
	0.4	0.2	7.2	1.4	15	9	6	x	x	x	x	1150	606	544	x	x
	0.8	0.1	9.2	1.1	47	32	15	x	x	x	x	7920	4060	3860	x	x
	1.7	0.2	11.9	1.5	45	30	15	x	x	x	x	7296	3702	3594	x	x
	3.7	0.2	16.4	2.0	37	26	11	x	x	x	x	6167	3364	3215	x	x
	7.5	0.3	25.7	4.5	47	33	14	x	x	x	x	6167	3119	3048	x	x
	12.8	0.2	49.2	10.9	47	31	16	x	x	x	x	5089	2489	2600	x	x
	15.5	0.4	61.4	11.7	37	23	14	x	x	x	x	4109	1938	2171	x	x
	24.5	0.8	73.1	16.4	31	19	12	x	x	x	x	2976	1145	1831	x	x
**Height - cm**	0.1	0.0	57.4	2.9	49	34	15	x	x	x	x	8123	4152	3971	x	x
	0.4	0.2	64.7	4.2	15	9	6	x	x	x	x	1150	606	544	x	x
	0.8	0.1	72.3	3.2	47	32	15	x	x	x	x	7870	4035	3835	x	x
	1.7	0.2	83.8	4.2	44	29	15	x	x	x	x	7238	3691	3547	x	x
	3.7	0.2	100.4	4.2	37	26	11	x	x	x	x	6580	3359	3221	x	x
	7.5	0.3	125.6	5.4	47	33	14	x	x	x	x	6174	3124	3050	x	x

© 2022 Elhakeem A et al. *JAMA Network Open.*

	12.8	0.2	157.3	7.6	47	31	16	x	x	x	x	5143	2505	2638	x	x
	15.5	0.4	169.2	8.4	37	23	14	x	x	x	x	4117	1941	2176	x	x
	24.5	0.8	171.3	9.2	31	19	12	x	x	x	x	2976	1146	1830	x	x
**BMI - kg/m2**	0.1	0.0	15.2	1.8	49	34	15	x	x	x	x	8106	4141	3965	x	x
	0.4	0.2	17.2	1.6	15	9	6	x	x	x	x	1150	606	544	x	x
	0.8	0.1	17.5	1.8	46	31	15	x	x	x	x	7696	3943	3753	x	x
	1.7	0.2	16.9	1.8	44	29	11	x	x	x	x	7060	3591	3469	x	x
	3.7	0.2	16.2	1.4	37	26	14	x	x	x	x	6492	3315	3177	x	x
	7.5	0.3	16.2	2.0	47	33	16	x	x	x	x	6166	3118	3048	x	x
	12.8	0.2	19.8	3.5	47	31	16	x	x	x	x	5089	2489	2600	x	x
	15.5	0.4	21.4	3.5	37	23	14	x	x	x	x	4109	1938	2171	x	x
	24.5	0.8	24.9	5.0	31	19	12	x	x	x	x	2974	1145	1829	x	x
**Waist circumference - cm**	2.6	0.3	50.5	3.1	13	8	5	x	x	x	x	1,007	538	469	x	x
	7.5	0.3	56.3	5.2	47	33	14	x	x	x	x	6170	3120	3050	x	x
	12.8	0.2	70.6	9.5	47	31	16	x	x	x	x	5102	2491	2611	x	x
	24.5	0.8	81.3	12.6	31	19	12	x	x	x	x	2967	1144	1823	x	x
**Body fat %**	7.5	0.3	13.8	8.0	46	32	13	x	x	x	x	5866	2896	2950	x	x
	12.8	0.2	22.4	8.3	47	31	16	x	x	x	x	5080	2483	2597	x	x
	17.8	0.5	25.6	11.6	30	21	9	x	x	x	x	3723	1649	2074	x	x
**Fat mass index kg/m2**	11.7	0.2	5.1	2.8	44	30	14	x	x	x	x	5303	2597	2706	x	x
	24.5	0.8	8.0	3.8	30	18	12	x	x	x	x	2892	1118	1774	x	x
**BASELINE**																
**Cardio-metabolic outcome**	**Age at outcome assessment - y**		**Outcome at assessment - kg, cm etc.**		**Numbers included in the analyses: adjusted models**											
	**Mean**	**SD**	**Mean**	**SD**	**ART**	**ART: males**	**ART: females**	**ICSI**	**IVF**	**FET**	**ET**	**NC**	**NC: males**	**NC: females**	**NC: fertile parents**	**NC: sub-fertile parent**

© 2022 Elhakeem A et al. *JAMA Network Open.*

**Weight - kg**	0.2	0.0	5.5	0.7	16	10	6	x	x	x	x	961	499	462	825	136
	0.5	0.0	8.0	0.9	18	11	7	x	x	x	x	1026	526	500	889	137
	1.1	0.1	10.1	1.1	19	11	8	x	x	x	x	961	497	464	832	129
	2.2	0.1	13.0	1.4	20	12	8	x	x	x	x	1031	527	504	890	141
	5.1	0.2	19.8	2.6	13	7	6	x	x	x	x	813	409	404	714	99
**Height - cm**	0.2	0.0	58.5	2.3	16	10	6	x	x	x	x	961	499	462	825	136
	0.5	0.0	67.9	2.3	18	11	7	x	x	x	x	1025	525	500	888	137
	1.1	0.1	76.6	2.8	19	11	8	x	x	x	x	963	498	465	833	130
	2.2	0.1	88.1	3.3	20	12	8	x	x	x	x	1014	514	500	874	140
	5.1	0.2	111.0	4.4	13	7	6	x	x	x	x	812	408	404	714	98
**BMI - kg/m2**	0.2	0.0	15.9	1.4	16	10	6	x	x	x	x	961	499	462	825	136
	0.5	0.0	17.4	1.5	18	11	7	x	x	x	x	1024	525	499	887	137
	1.1	0.1	17.2	1.4	19	11	8	x	x	x	x	958	496	462	829	129
	2.2	0.1	16.7	1.2	20	12	8	x	x	x	x	1004	508	496	867	137
	5.1	0.2	16.0	1.4	13	7	6	x	x	x	x	812	408	404	714	98
**Waist circumference - cm**	2.2	0.1	49.1	3.1	20	12	8	x	x	x	x	998	510	488	860	138
	5.1	0.2	55.1	4.0	13	7	6	x	x	x	x	808	405	403	710	98
**Body fat %**	0.2	0.0	21.8	4.3	15	9	6	x	x	x	x	793	420	373	682	111
	5.1	0.2	26.2	4.2	10	5	5	x	x	x	x	415	205	210	363	52
**Fat mass index kg/m2**	0.2	0.0	3.5	0.9	15	9	6	x	x	x	x	793	420	373	682	111
	5.1	0.2	4.3	1.0	10	5	5	x	x	x	x	415	205	210	363	52
**BIS**																
**Cardio-metabolic outcome**	**Age at outcome assessment - y**		**Outcome at assessment - kg, cm etc.**		**Numbers included in the analyses: adjusted models**											

© 2022 Elhakeem A et al. *JAMA Network Open.*

	Mean	SD	Mean	SD	ART	ART: males	ART: females	ICSI	IVF	FET	ET	NC	NC: males	NC: females	NC: fertile parents	NC: sub-fertile parent
**Weight - kg**	1.1	0.1	10.1	1.3	35	17	18	x	x	x	x	673	354	319	x	x
	4.2	0.3	17.7	2.6	25	14	11	x	x	x	x	447	235	212	x	x
**Height - cm**	1.1	0.1	75.6	3.1	35	17	18	x	x	x	x	665	348	317	x	x
	4.2	0.3	106.3	6.1	25	14	11	x	x	x	x	443	233	210	x	x
**BMI - kg/m2**	1.1	0.1	17.7	1.8	35	17	18	x	x	x	x	627	330	297	x	x
	4.2	0.3	15.6	1.5	26	13	13	x	x	x	x	535	280	255	x	x
**Body fat - %**	4.2	0.3	19.8	3.7	19	9	10	x	x	x	x	359	179	180	x	x
**BIGCS**																
**Cardio-metabolic outcome**	**Age at outcome assessment - y**		**Outcome at assessment - kg, cm etc.**		**Numbers included in the analyses: adjusted models**											
	**Mean**	**SD**	**Mean**	**SD**	**ART**	**ART: males**	**ART: females**	**ICSI**	**IVF**	**FET**	**ET**	**NC**	**NC: males**	**NC: females**	**NC: fertile parents**	**NC: sub-fertile parent**
**Weight - kg**	0.1	0.0	4.6	0.6	349	159	190	x	x	x	x	9725	5106	4619	7900	467
	0.5	0.0	8.0	1.0	375	182	193	x	x	x	x	9875	5214	4661	8175	513
	1.0	0.0	9.5	1.1	273	126	147	x	x	x	x	6933	3655	3278	5390	350
	2.8	0.2	13.5	1.7	267	135	132	x	x	x	x	6133	3186	2947	5193	342
**Height - cm**	0.1	0.0	54.9	2.2	345	158	187	x	x	x	x	9739	5113	4626	7884	464
	0.5	0.0	67.3	2.5	379	183	196	x	x	x	x	9884	5220	4664	8181	516
	1.0	0.0	74.8	2.7	272	126	146	x	x	x	x	6950	3657	3293	5408	346
	2.8	0.2	93.6	4.1	268	134	134	x	x	x	x	6085	3157	2928	5164	341
**BMI - kg/m2**	0.1	0.0	15.3	1.5	344	158	186	x	x	x	x	9668	5075	4593	7854	465
	0.5	0.0	17.6	1.7	373	180	193	x	x	x	x	9770	5164	4606	8086	509
	1.0	0.0	17.0	1.5	271	125	146	x	x	x	x	6848	3607	3241	5323	344
	2.8	0.2	15.5	1.3	268	135	133	x	x	x	x	6055	3139	2916	5135	342

© 2022 Elhakeem A et al. *JAMA Network Open.*

CHART																
**Cardio-metabolic outcome**	**Age at outcome assessment - y**		**Outcome at assessment - kg, cm etc.**		**Numbers included in the analyses: adjusted models**											
	**Mean**	**SD**	**Mean**	**SD**	**ART**	**ART: males**	**ART: females**	**ICSI**	**IVF**	**FET**	**ET**	**NC**	**NC: males**	**NC: females**	**NC: fertile parents**	**NC: sub-fertile parent**
**Weight - kg**	27.4	2.8	75.3	16.4	130	55	75	x	x	38	92	73	25	48	x	x
**Height - cm**	27.4	2.8	171.7	9.9	130	55	75	x	x	38	92	73	25	48	x	x
**BMI - kg/m2**	27.4	2.8	25.5	5.0	120	52	68	x	x	35	85	68	23	45	x	x
**Waist circumference - cm**	27.6	2.7	80.9	12.5	117	49	68	x	x	35	82	67	24	43	x	x
**Body fat - %**	27.6	2.6	26.7	10.3	121	52	69	x	x	35	86	68	24	44	x	x
**Fat mass index - kg/m2**	27.6	2.6	7.5	4.2	121	52	69	x	x	35	86	68	24	44	x	x
**DNBC**																
**Cardio-metabolic outcome**	**Age at outcome assessment - y**		**Outcome at assessment - kg, cm etc.**		**Numbers included in the analyses: adjusted models**											
	**Mean**	**SD**	**Mean**	**SD**	**ART**	**ART: males**	**ART: females**	**ICSI**	**IVF**	**FET**	**ET**	**NC**	**NC: males**	**NC: females**	**NC: fertile parents**	**NC: sub-fertile parent**
**Weight - kg**	0.4	0.1	7.8	1.0	1292	671	621	x	x	x	x	45203	23016	22187	x	x
	1.0	0.1	10.3	1.2	1211	632	579	x	x	x	x	42116	21369	20747	x	x
	7.0	0.3	24.8	4.3	1481	752	729	x	x	x	x	34899	17943	16956	x	x
	11.3	0.6	30.9	7.6	1393	712	681	x	x	x	x	35821	17779	18042	x	x
**Height - cm**	0.4	0.1	68.2	2.9	1292	671	621	x	x	x	x	45203	23016	22187	x	x
	1.0	0.1	77.6	3.1	1211	632	579	x	x	x	x	42116	21369	20747	x	x
	7.0	0.3	125.5	5.7	1481	752	729	x	x	x	x	34899	17943	16956	x	x

	11.3	0.6	149.8	7.5	1393	712	681	x	x	x	x	35821	17779	18042	x	x
**BMI - kg/m2**	0.4	0.1	24.5	2.7	1292	671	621	x	x	x	x	45203	23016	22187	x	x
	1.0	0.1	22.0	2.4	1211	632	579	x	x	x	x	42116	21369	20747	x	x
	7.0	0.3	12.5	1.7	1481	752	729	x	x	x	x	34899	17943	16956	x	x
	11.3	0.6	11.6	1.6	1393	712	681	x	x	x	x	35821	17779	18042	x	x
**Waist circumference - cm**	7.1	0.2	57.03	5.23	1663	858	805	x	x	x	x	41063	20943	20120	x	x
**EDEN**																
**Cardio-metabolic outcome**	**Age at outcome assessment - y**		**Outcome at assessment - kg, cm etc.**		**Numbers included in the analyses: adjusted models**											
	**Mean**	**SD**	**Mean**	**SD**	**ART**	**ART: males**	**ART: females**	**ICSI**	**IVF**	**FET**	**ET**	**NC**	**NC: males**	**NC: females**	**NC: fertile parents**	**NC: sub-fertile parent**
**Weight - kg**	0.3	0.0	6.2	0.9	22	9	13	x	x	x	x	1326	692	634	1014	172
	0.7	0.0	8.7	1.0	17	8	9	x	x	x	x	1171	617	554	887	160
	1.0	0.0	9.8	1.1	21	8	13	x	x	x	x	1362	724	641	1045	180
	2.0	0.0	12.3	1.4	17	7	10	x	x	x	x	1163	611	552	898	151
	3.2	0.1	14.9	1.6	18	8	11	x	x	x	x	1088	572	519	833	142
	5.7	0.2	20.3	2.7	14	6	8	x	x	x	x	980	531	450	760	129
**Height - cm**	0.3	0.0	61.6	2.9	21	8	13	x	x	x	x	1325	691	634	1014	171
	0.7	0.0	71.0	2.7	17	8	9	x	x	x	x	1141	603	540	861	158
	1.0	0.0	74.9	2.7	21	8	14	x	x	x	x	1361	726	641	1045	179
	2.0	0.0	87.5	3.3	17	7	10	x	x	x	x	1154	607	549	890	151
	3.2	0.1	96.9	3.7	18	8	11	x	x	x	x	1085	573	518	832	140
	5.7	0.2	114.4	4.9	14	6	8	x	x	x	x	980	531	450	760	129
**BMI - kg/m2**	0.3	0.0	16.3	1.5	21	8	13	x	x	x	x	1325	691	634	1014	171
	0.7	0.0	17.2	1.5	17	8	9	x	x	x	x	1141	602	539	861	158

© 2022 Elhakeem A et al. *JAMA Network Open.*

	1.0	0.0	17.5	1.5	21	8	13	x	x	x	x	1361	723	641	1045	179
	2.0	0.0	16.0	1.3	17	7	10	x	x	x	x	1154	607	547	890	151
	3.2	0.1	15.9	1.2	18	8	11	x	x	x	x	1085	570	518	832	140
	5.7	0.2	15.4	1.3	14	6	8	x	x	x	x	980	531	450	760	129
	3.2	0.1	49.8	3.0	18	8	11	x	x	x	x	1086	575	518	832	141
	5.7	0.2	53.8	3.7	14	6	8	x	x	x	x	979	531	449	759	129
**Body fat - %**	5.7	0.2	14.4	3.7	13	6	7	x	x	x	x	945	513	433	734	124
**Fat mass index - kg/m2**	5.7	0.2	2.3	0.7	13	6	7	x	x	x	x	945	513	433	734	124
**ELFE**																
**Cardio-metabolic outcome**	**Age at outcome assessment - y**		**Outcome at assessment - kg, cm etc.**		**Numbers included in the analyses: adjusted models**											
	**Mean**	**SD**	**Mean**	**SD**	**ART**	**ART: males**	**ART: females**	**ICSI**	**IVF**	**FET**	**ET**	**NC**	**NC: males**	**NC: females**	**NC: fertile parents**	**NC: sub-fertile parent**
**Weight - kg**	0.3	0.0	6.6	0.8	309	168	141	210	99	x	x	9632	4907	4725	6580	1210
	0.8	0.0	8.7	1.0	302	161	141	200	102	x	x	9246	4700	4546	6323	1179
	1.0	0.0	9.6	1.1	271	150	121	185	86	x	x	8112	4088	4024	5614	993
	2.0	0.0	12.2	1.4	230	120	110	153	77	x	x	6827	3467	3360	4761	823
	3.0	0.1	14.2	1.8	89	48	41	56	33	x	x	2228	1110	1118	1574	271
**Height - cm**	0.3	0.0	62.9	2.6	304	166	138	207	97	x	x	9322	4733	4589	6377	1174
	0.8	0.0	71.3	2.6	295	155	140	195	100	x	x	8940	4527	4413	6118	1143
	1.0	0.0	75.2	2.8	266	148	118	181	85	x	x	7866	3963	3903	5456	958
	2.0	0.0	87.3	3.3	227	118	109	152	75	x	x	6745	3424	3321	4703	815
	3.0	0.1	96.1	3.9	89	48	41	56	33	x	x	2217	1101	1116	1566	271
**BMI - kg/m2**	0.3	0.0	16.6	1.5	304	166	138	207	97	x	x	9322	4733	4589	6377	1174

© 2022 Elhakeem A et al. *JAMA Network Open.*

	0.8	0.0	17.1	1.5	295	155	140	195	100	x	x	8940	4527	4413	6118	1143
	1.0	0.0	16.9	1.4	266	148	118	181	85	x	x	7866	3963	3903	5456	958
	2.0	0.0	16.0	1.3	227	118	109	152	75	x	x	6745	3424	3321	4703	815
	3.0	0.1	15.4	1.5	89	48	41	56	33	x	x	2217	1101	1116	1566	271
**CHOP**																
**Cardio-metabolic outcome**	**Age at outcome assessment - y**		**Outcome at assessment - kg, cm etc.**		**Numbers included in the analyses: adjusted models**											
	**Mean**	**SD**	**Mean**	**SD**	**ART**	**ART: males**	**ART: females**	**ICSI**	**IVF**	**FET**	**ET**	**NC**	**NC: males**	**NC: females**	**NC: fertile parents**	**NC: sub-fertile parent**
**Weight - kg**	0.1	0.0	3.6	0.7	20	x	x	x	x	x	x	1479	x	x	x	x
	0.3	0.0	6.0	0.7	16	x	x	x	x	x	x	1217	x	x	x	x
	0.5	0.0	7.7	0.9	15	x	x	x	x	x	x	1107	x	x	x	x
	1.0	0.0	9.8	1.1	15	x	x	x	x	x	x	1003	x	x	x	x
**Height - cm**	0.1	0.0	51.9	2.8	20	x	x	x	x	x	x	1479	x	x	x	x
	0.3	0.0	60.6	2.2	16	x	x	x	x	x	x	1214	x	x	x	x
	0.5	0.0	67.3	2.3	15	x	x	x	x	x	x	1107	x	x	x	x
	1.0	0.0	75.5	2.7	15	x	x	x	x	x	x	1004	x	x	x	x
**BMI - kg/m2**	0.1	0.0	13.4	1.5	20	x	x	x	x	x	x	1472	x	x	x	x
	0.3	0.0	16.3	1.4	16	x	x	x	x	x	x	1214	x	x	x	x
	0.5	0.0	17.0	1.5	15	x	x	x	x	x	x	1107	x	x	x	x
	1.0	0.0	17.2	1.5	15	x	x	x	x	x	x	1003	x	x	x	x
**Waist circumference - cm**	0.1	0.0	33.2	3.3	19	x	x	x	x	x	x	1461	x	x	x	x
	0.3	0.0	39.9	2.8	16	x	x	x	x	x	x	1217	x	x	x	x
	0.5	0.0	42.7	3.2	15	x	x	x	x	x	x	1105	x	x	x	x
	1.0	0.0	45.7	3.7	15	x	x	x	x	x	x	1001	x	x	x	x
**GASPII**																

© 2022 Elhakeem A et al. *JAMA Network Open.*

Cardio-metabolic outcome	Age at outcome assessment - y		Outcome at assessment - kg, cm etc.		Numbers included in the analyses: adjusted models											
	Mean	SD	Mean	SD	ART	ART: males	ART: females	ICSI	IVF	FET	ET	NC	NC: males	NC: females	NC: fertile parents	NC: sub-fertile parent
**Weight - kg**	1.4	0.1	11.0	1.2	8	x	x	x	x	x	x	554	x	x	495	48
	4.1	0.2	17.8	2.4	8	x	x	x	x	x	x	528	x	x	467	50
	7.8	0.2	29.0	5.6	8	x	x	x	x	x	x	453	x	x	400	42
**Height - cm**	1.4	0.1	80.9	3.1	7	x	x	x	x	x	x	545	x	x	488	48
	4.1	0.2	104.3	4.5	8	x	x	x	x	x	x	528	x	x	467	50
	7.8	0.2	128.0	5.8	8	x	x	x	x	x	x	454	x	x	401	42
**BMI - kg/m2**	1.4	0.1	16.8	1.3	7	x	x	x	x	x	x	543	x	x	486	48
	4.1	0.2	16.4	1.6	8	x	x	x	x	x	x	528	x	x	467	50
	7.8	0.2	17.6	2.5	8	x	x	x	x	x	x	453	x	x	400	42
**Waist circumference - cm**	7.8	0.2	60.3	6.5	8	x	x	x	x	x	x	450	x	x	397	42
**Gen-R**																
**Cardio-metabolic outcome**	**Age at outcome assessment - y**		**Outcome at assessment - kg, cm etc.**		**Numbers included in the analyses: adjusted models**											
	**Mean**	**SD**	**Mean**	**SD**	**ART**	**ART: males**	**ART: females**	**ICSI**	**IVF**	**FET**	**ET**	**NC**	**NC: males**	**NC: females**	**NC: fertile parents**	**NC: sub-fertile parent**
**Weight - kg**	0.1	0.0	4.4	0.6	47	23	24	x	x	x	x	3788	1908	1880	x	x
	0.5	0.1	7.9	0.9	51	23	28	x	x	x	x	4256	2147	2109	x	x
	0.9	0.1	9.7	1.1	45	23	22	x	x	x	x	3891	1966	1925	x	x
	2.1	0.1	13.0	1.5	40	16	24	x	x	x	x	3494	1768	1726	x	x
	6.2	0.5	23.3	4.3	45	20	25	x	x	x	x	4334	2160	2174	x	x
	9.8	0.4	35.4	7.4	42	19	23	x	x	x	x	3754	1842	1912	x	x
**Height - cm**	0.1	0.0	54.3	2.5	41	21	20	x	x	x	x	3150	1576	1574	x	x

© 2022 Elhakeem A et al. *JAMA Network Open.*

	0.5	0.1	67.7	2.7	45	19	26	x	x	x	x	3771	1894	1877	x	x
	0.9	0.1	74.4	2.7	45	23	22	x	x	x	x	3886	1964	1922	x	x
	2.1	0.1	88.3	3.5	40	16	24	x	x	x	x	3439	1736	1703	x	x
	6.2	0.5	119.5	6.1	45	20	25	x	x	x	x	4334	2160	2174	x	x
	9.8	0.4	141.6	6.7	42	19	23	x	x	x	x	3754	1842	1912	x	x
**BMI - kg/m2**	0.1	0.0	15.0	1.4	41	21	20	x	x	x	x	3146	1574	1572	x	x
	0.5	0.1	17.2	1.4	45	19	26	x	x	x	x	3738	1868	1870	x	x
	0.9	0.1	17.4	1.4	45	23	22	x	x	x	x	3864	1951	1913	x	x
	2.1	0.1	16.6	1.4	40	16	24	x	x	x	x	3432	1733	1699	x	x
	6.2	0.5	16.2	1.9	45	20	25	x	x	x	x	4334	2160	2174	x	x
	9.8	0.4	17.6	2.8	42	19	23	x	x	x	x	3754	1842	1912	x	x
**Fat mass index - kg/m2**	6.2	0.5	4.1	1.4	42	20	22	x	x	x	x	4217	2099	2118	x	x
	9.8	0.3	4.9	2.1	41	19	22	x	x	x	x	3713	1819	1894	x	x
**G21**																
**Cardio-metabolic outcome**	**Age at outcome assessment - y**		**Outcome at assessment - kg, cm etc.**		**Numbers included in the analyses: adjusted models**											
	**Mean**	**SD**	**Mean**	**SD**	**ART**	**ART: males**	**ART: females**	**ICSI**	**IVF**	**FET**	**ET**	**NC**	**NC: males**	**NC: females**	**NC: fertile parents**	**NC: sub-fertile parent**
**Weight - kg**	0.2	0	5.3	0.8	68	28	40	26	42	x	x	4887	2532	2355	4566	1869
	0.4	0.1	7.2	0.9	70	30	40	29	41	x	x	5163	2661	2502	4819	1957
	0.6	0.1	8.4	1.0	68	30	38	27	41	x	x	4983	2568	2415	4666	1857
	0.8	0.1	9.2	1.1	57	26	31	25	32	x	x	4264	2202	2062	3984	1559
	1.2	0.1	10.6	1.3	72	31	41	29	43	x	x	4996	2560	2436	4662	1849
	1.7	0.2	12	1.5	68	30	38	27	41	x	x	4904	2528	2376	4572	1830
	2.6	0.3	14.1	2.0	69	31	38	29	40	x	x	4769	2482	2287	4460	1757
	3.5	0.3	16.5	2.6	68	30	38	28	40	x	x	4350	2295	2055	4050	1570
	4.4	0.4	18.2	3.0	89	41	48	37	52	x	x	5158	2626	2532	4826	1967
	7.3	0.4	26.7	5.6	98	45	53	41	57	x	x	5746	2934	2812	5389	2166

© 2022 Elhakeem A et al. *JAMA Network Open.*

	10.2	0.3	37.7	8.9	92	43	49	37	55	x	x	4664	2391	2273	4373	1697
**Height - cm**	0.2	0	57.3	2.9	68	28	40	26	42	x	x	4887	2532	2355	4566	1869
	0.4	0.1	64.7	2.9	70	30	40	29	41	x	x	5163	2661	2502	4819	1957
	0.6	0.1	69.2	3	68	30	38	27	41	x	x	4983	2568	2415	4666	1857
	0.8	0.1	72.2	2.9	57	26	31	25	32	x	x	4264	2202	2062	3984	1559
	1.2	0.1	78	3.2	72	31	41	29	43	x	x	4996	2560	2436	4662	1849
	1.7	0.2	84.3	3.9	68	30	38	27	41	x	x	4904	2528	2376	4572	1830
	2.6	0.3	92.6	4.8	69	31	38	29	40	x	x	4769	2482	2287	4460	1757
	3.5	0.3	100.5	5.1	68	30	38	28	40	x	x	4350	2295	2055	4050	1570
	4.4	0.4	105.3	5.1	89	41	48	37	52	x	x	5150	2622	2528	4820	1963
	7.2	0.4	124.2	6.0	98	45	53	41	57	x	x	5606	2883	2723	5251	2107
	10.2	0.3	141.1	6.7	92	43	49	37	55	x	x	4663	2390	2273	4372	1697
**BMI - kg/m2**	0.2	0	15.9	1.5	68	28	40	26	42	x	x	4887	2532	2355	4566	1869
	0.4	0.1	17.1	1.5	70	30	40	29	41	x	x	5163	2661	2502	4819	1957
	0.6	0.1	17.5	1.5	68	30	38	27	41	x	x	4983	2568	2415	4666	1857
	0.8	0.1	17.5	1.5	57	26	31	25	32	x	x	4264	2202	2062	3984	1559
	1.2	0.1	17.3	1.5	72	31	41	29	43	x	x	4996	2560	2436	4662	1849
	1.7	0.2	16.8	1.5	68	30	38	27	41	x	x	4904	2528	2376	4572	1830
	2.6	0.3	16.4	1.6	69	31	38	29	40	x	x	4769	2482	2287	4460	1757
	3.5	0.3	16.3	1.7	68	30	38	28	40	x	x	4350	2295	2055	4050	1570
	4.4	0.4	16.3	1.8	89	41	48	37	52	x	x	5150	2622	2528	4820	1963
	7.2	0.4	17.1	2.6	98	45	53	41	57	x	x	5591	2875	2716	5237	2097
	10.2	0.3	18.8	3.4	92	43	49	37	55	x	x	4663	2390	2273	4372	1697
**Waist circumference - cm**	4.4	0.4	52.8	4.5	89	41	48	37	52	x	x	5138	2613	2525	4808	1959
	7.1	0.2	59.1	6.9	92	41	51	40	52	x	x	5056	2618	2438	4737	1891
	10.2	0.3	68.0	9.8	92	43	49	37	55	x	x	4651	2385	2266	4361	1691

© 2022 Elhakeem A et al. *JAMA Network Open.*

**Body fat %**	4.4	0.4	16.6	8.0	76	32	44	31	45	x	x	4311	2161	2150	4033	1641
	7.1	0.2	16.3	10.6	91	41	50	40	51	x	x	4977	2576	2401	4663	1865
	10.2	0.3	22.2	9.8	92	43	49	37	55	x	x	4639	2373	2266	4351	1689
**Fat mass index - kg/m2**	4.4	0.4	2.8	1.6	76	32	44	31	45	x	x	4310	2161	2149	4032	1641
	7.1	0.2	3.0	2.3	91	41	50	40	51	x	x	4977	2576	2401	4663	1865
	10.2	0.3	4.5	2.8	92	43	49	37	55	x	x	4639	2373	2266	4351	1689
**GUI**																
**Cardio-metabolic outcome**	**Age at outcome assessment - y**		**Outcome at assessment - kg, cm etc.**		**Numbers included in the analyses: adjusted models**											
	**Mean**	**SD**	**Mean**	**SD**	**ART**	**ART: males**	**ART: females**	**ICSI**	**IVF**	**FET**	**ET**	**NC**	**NC: males**	**NC: females**	**NC: fertile parents**	**NC: sub-fertile parent**
**Weight - kg**	0.8	0.0	9.7	1.2	173	84	89	56	107	x	x	9742	4997	4745	x	x
	3.0	0.0	15.6	2.0	158	77	81	52	96	x	x	8480	4311	4169	x	x
	5.2	0.1	20.2	3.0	151	73	78	49	92	x	x	7864	3999	3865	x	x
**Height - cm**	0.8	0.0	72.9	3.5	173	84	89	56	107	x	x	9742	4997	4745	x	x
	3.0	0.0	96.2	3.9	158	76	82	51	97	x	x	8480	4311	4169	x	x
	5.2	0.1	111.3	4.7	151	73	78	49	92	x	x	7864	3999	3865	x	x
**BMI - kg/m2**	0.8	0.0	18.2	2.3	173	84	89	56	107	x	x	9735	4991	4744	x	x
	3.0	0.0	16.8	1.6	157	76	81	51	96	x	x	9437	4282	4155	x	x
	5.2	0.1	16.2	1.7	151	73	78	49	92	x	x	7850	3990	3860	x	x
**GUiNZ**																
**Cardio-metabolic outcome**	**Age at outcome assessment - y**		**Outcome at assessment - kg, cm etc.**		**Numbers included in the analyses: adjusted models**											
	**Mean**	**SD**	**Mean**	**SD**	**ART**	**ART: males**	**ART: females**	**ICSI**	**IVF**	**FET**	**ET**	**NC**	**NC: males**	**NC: females**	**NC: fertile parents**	**NC: sub-fertile parent**
**Weight - kg**	2.0	0.2	13.3	1.8	151	79	72	x	x	x	x	3643	1867	1776	x	x
	4.5	0.1	19.2	3.1	173	91	82	x	x	x	x	4274	2180	2094	x	x
	8.6	0.4	31.6	7.9	157	85	72	x	x	x	x	3755	1920	1835	x	x

© 2022 Elhakeem A et al. *JAMA Network Open.*

**Height - cm**	2.0	0.2	86.5	4.2	151	79	72	x	x	x	x	3640	1865	1775	x	x
	4.5	0.1	106.9	4.9	173	91	82	x	x	x	x	4274	2180	2094	x	x
	8.6	0.4	133.3	6.7	157	85	72	x	x	x	x	3751	1920	1831	x	x
**BMI - kg/m2**	2.04	0.18	1.35	1.37	152	80	72	x	x	x	x	3721	1911	1810	x	x
	4.50	0.13	0.94	1.20	173	91	82	x	x	x	x	4274	2180	2094	x	x
	8.57	0.40	0.63	1.35	157	85	72	x	x	x	x	3751	1920	1831	x	x
**Waist circumference - cm**	4.50	0.13	54.4	4.4	173	91	82	x	x	x	x	4256	2168	2088	x	x
	8.57	0.40	60.8	8.3	157	85	72	x	x	x	x	3704	1889	1815	x	x
**GUSTO**																
**Cardio-metabolic outcome**	**Age at outcome assessment - y**		**Outcome at assessment - kg, cm etc.**		**N at assessment: adjusted models**											
	**Mean**	**SD**	**Mean**	**SD**	**ART**	**ART: males**	**ART: females**	**ICSI**	**IVF**	**FET**	**ET**	**NC**	**NC: males**	**NC: females**	**NC: fertile parents**	**NC: sub-fertile parent**
**Weight - kg**	0.1	0.0	3.9	0.5	63	35	26	52	9	13	43	935	487	448	x	x
	0.3	0.0	6.1	0.8	66	39	25	54	10	13	46	918	478	440	x	x
	0.5	0.0	7.7	0.9	62	35	26	53	8	13	44	880	455	425	x	x
	0.8	0.0	8.6	1.0	64	36	27	54	9	14	45	841	431	410	x	x
	1.0	0.0	9.4	1.1	62	35	26	52	9	13	44	862	444	418	x	x
	1.5	0.1	10.7	1.3	55	34	20	45	9	13	38	820	425	395	x	x
	2.0	0.1	11.9	1.6	58	34	23	48	9	13	40	832	433	399	x	x
	3.0	0.1	14.2	2.1	59	35	23	49	9	12	42	839	441	398	x	x
	4.6	0.1	17.4	3.0	61	35	25	52	8	12	44	805	415	390	x	x
	6.1	0.1	20.9	4.3	54	32	22	46	8	12	38	748	385	363	x	x
**Height - cm**	0.1	0.0	52.9	2.2	63	35	26	52	9	13	43	933	487	446	x	x
	0.3	0.0	60.9	2.5	66	39	25	54	10	13	46	918	478	440	x	x
	0.5	0.0	67.1	2.7	62	35	26	53	8	13	44	884	458	426	x	x
	0.8	0.0	71.6	2.9	64	36	27	54	9	14	45	842	432	410	x	x

© 2022 Elhakeem A et al. *JAMA Network Open.*

	1.0	0.0	75.4	3.1	62	35	26	52	9	13	44	862	444	418	x	x
	1.5	0.1	82.1	3.3	50	30	19	40	9	13	33	777	404	373	x	x
	2.0	0.1	87.6	3.6	56	33	22	46	9	12	39	801	419	382	x	x
	3.0	0.1	94.8	3.8	59	35	23	49	9	12	42	835	439	396	x	x
	4.6	0.1	105.5	4.4	61	35	25	52	8	12	44	807	416	391	x	x
	6.1	0.1	115.6	5.1	54	32	22	46	8	12	38	747	384	363	x	x
**BMI - kg/m2**	0.1	0.0	13.9	1.3	63	35	26	52	9	13	43	932	486	446	x	x
	0.3	0.0	16.5	1.6	66	39	25	54	10	13	46	918	478	440	x	x
	0.5	0.0	17.1	1.6	62	35	26	53	8	13	44	880	455	425	x	x
	0.8	0.0	16.7	1.5	64	36	27	54	9	14	45	841	431	410	x	x
	1.0	0.0	16.4	1.4	62	35	26	52	9	13	44	860	443	417	x	x
	1.5	0.1	15.9	1.3	50	30	19	40	9	13	33	772	400	372	x	x
	2.0	0.1	15.5	1.4	56	33	22	46	9	12	39	800	418	382	x	x
	3.0	0.1	15.8	1.6	59	35	23	49	9	12	42	832	437	395	x	x
	4.6	0.1	15.5	1.9	61	35	25	52	8	12	44	805	415	390	x	x
	6.1	0.1	15.5	2.3	54	32	22	46	8	12	38	747	384	363	x	x
**Waist circumference - cm**	0.3	0.0	39.1	3.0	66	39	25	54	10	13	46	914	475	439	x	x
	6.1	0.1	54.0	6.4	53	31	22	45	8	12	37	743	382	361	x	x
**Fat mass index - kg/m2**	6.1	0.1	4.2	2.0	11	6	5	9	2	2	7	206	104	102	x	x
**HGS**																
**Cardio-metabolic outcome**	**Age at outcome assessment - y**		**Outcome at assessment - kg, cm etc.**		**Numbers included in the analyses: adjusted models**											
	**Mean**	**SD**	**Mean**	**SD**	**ART**	**ART: males**	**ART: females**	**ICSI**	**IVF**	**FET**	**ET**	**NC**	**NC: males**	**NC: females**	**NC: fertile parents**	**NC: sub-fertile parent**
**Weight - kg**	11.2	0.7	45.3	11	63	37	26	23	40	x	x	2182	1069	1113	x	x

© 2022 Elhakeem A et al. *JAMA Network Open.*

**Height - cm**	11.2	0.7	148.8	7.8	63	37	26	23	40	x	x	2182	1069	1113	x	x
**BMI - kg/m2**	11.2	0.7	20.3	3.8	63	37	26	23	40	x	x	2182	1069	1113	x	x
**Waist circumference - cm**	11.2	0.7	68.8	9.7	61	35	26	22	39	x	x	2161	1062	1099	x	x
**Body fat %**	11.2	0.7	29.3	9.1	61	35	26	22	39	x	x	2133	1054	1079	x	x
**Fat mass index kg/m2**	11.2	0.7	14.1	7.5	61	35	26	22	39	x	x	2133	1054	1079	x	x
**ITR**																
**Cardio-metabolic outcome**	**Age at outcome assessment - y**		**Outcome at assessment - kg, cm etc.**		**Numbers included in the analyses: adjusted models**											
	**Mean**	**SD**	**Mean**	**SD**	**ART**	**ART: males**	**ART: females**	**ICSI**	**IVF**	**FET**	**ET**	**NC**	**NC: males**	**NC: females**	**NC: fertile parents**	**NC: sub-fertile parent**
**Weight - kg**	0.6	0.3	7.5	2.2	50	20	30	x	20	x	x	166	84	82	x	x
	1.5	0.3	10.9	1.6	53	26	26.99999	x	16	x	x	237	117	124	x	x
	2.5	0.3	13.0	1.7	54	29.00001	25	x	34	x	x	194	94	100	x	x
	3.5	0.3	15.4	1.9	44	24	20	x	20	x	x	188	107	81	x	x
	5.5	0.3	20.0	3.1	46	19	27	x	20	x	x	143	80	63	x	x
	8.0	1.2	27.3	6.6	46	19	26.99999	x	22	x	x	367	180	187	x	x
	13.6	2.0	49.3	11.7	34	24.00002	10.00001	x	26	x	x	819	407	412	x	x
**Height - cm**	0.6	0.3	66.3	8.2	46	19	27	x	18	x	x	158	79	77	x	x
	1.5	0.3	80.2	5.3	54	26	28	x	16	x	x	232	112	124	x	x
	2.5	0.3	90.5	4.7	54	29	25	x	34	x	x	190	92	98	x	x
	3.5	0.3	98.4	5.1	44	24	20	x	20	x	x	186	107	79	x	x
	5.5	0.3	112.2	6.5	46	19	27	x	20	x	x	144	80	64	x	x
	8.0	1.2	127.8	9.8	46	19	27	x	22	x	x	366	181	185	x	x

© 2022 Elhakeem A et al. *JAMA Network Open.*

	13.6	2.0	159.1	11.9	32	22	10	x	24	x	x	820	406	414	x	x
**BMI - kg/m2**	0.6	0.3	16.5	2.4	48	19	29	x	20	x	x	156	79	77	x	x
	1.5	0.3	16.9	2.0	53	26	26.99999	x	16	x	x	232	112	124	x	x
	2.5	0.3	15.8	1.6	54	29	25	x	34	x	x	190	92	98	x	x
	3.5	0.3	15.9	2.2	42	22	20	x	18	x	x	186	107	79	x	x
	5.5	0.3	15.9	2.3	46	19	27	x	20	x	x	140	77	63	x	x
	8.0	1.2	16.5	2.4	46	19	27	x	22	x	x	365	180	185	x	x
	13.6	2.0	19.2	2.9	32	21.99998	9.99999	x	24	x	x	815	405	410	x	x
**MCS**																
**Cardio-metabolic outcome**	**Age at outcome assessment - y**		**Outcome at assessment - kg, cm etc.**		**Numbers included in the analyses: adjusted models**											
	**Mean**	**SD**	**Mean**	**SD**	**ART**	**ART: males**	**ART: females**	**ICSI**	**IVF**	**FET**	**ET**	**NC**	**NC: males**	**NC: females**	**NC: fertile parents**	**NC: sub-fertile parent**
**Weight - kg**	3.1	0.2	15.1	2.4	30	12	18	8	22	x	x	2153	1123	1030	1841	303
	5.2	0.2	20.1	3.2	30	13	17	6	24	x	x	2060	1081	979	1770	281
	6.8	0.4	25.5	4.7	27	12	15	6	21	x	x	1925	1004	921	1648	269
	11.2	0.3	41.2	9.6	27	10	17	7	20	x	x	1826	965	861	1568	251
	17.2	0.3	67	14.5	20	8	12	7	14	x	x	1391	731	660	1208	179
**Height - cm**	3.1	0.2	95.6	4.2	30	12	18	8	22	x	x	2132	1112	1020	1820	303
	5.2	0.2	110.8	5	30	13	17	6	24	x	x	2060	1081	979	1769	282
	6.8	0.4	123.7	5.6	28	12	16	6	22	x	x	1929	1007	922	1651	270
	11.2	0.3	146.5	7.2	27	10	17	7	20	x	x	1861	976	885	1598	256
	17.2	0.3	170.3	9.4	20	8	12	7	14	x	x	1427	740	687	1239	184
**BMI - kg/m2**	3.1	0.2	16.8	2	30	12	18	8	22	x	x	2107	1098	1009	1799	299
	5.2	0.2	16.3	1.8	30	13	17	6	24	x	x	2058	1081	977	1768	281
	6.8	0.4	16.6	2.2	27	12	15	6	21	x	x	1925	1004	921	1648	269
	11.2	0.3	19.1	3.5	27	10	17	7	20	x	x	1826	965	861	1568	251

© 2022 Elhakeem A et al. *JAMA Network Open.*

	17.2	0.3	23	4.4	20	8	12	7	14	x	x	1390	730	660	1208	178
**Waist circumference - cm**	5.2	0.2	21.2	1.8	30	13	17	6	24	x	x	2046	1074	972	1759	278
	6.8	0.4	57.1	5.9	26	12	14	6	20	x	x	1916	1001	915	1641	267
**Body fat %**	6.8	0.4	21	5.3	27	12	15	6	21	x	x	1895	985	910	1623	265
	11.2	0.3	22	7.7	27	10	17	7	20	x	x	1806	953	853	1551	248
	17.2	0.3	21.7	9.9	20	8	12	7	14	x	x	1370	717	653	1191	175
**MUBICOS**																
**Cardio-metabolic outcome**	**Age at outcome assessment - y**		**Outcome at assessment - kg, cm etc.**		**Numbers included in the analyses: adjusted models**											
	**Mean**	**SD**	**Mean**	**SD**	**ART**	**ART: males**	**ART: females**	**ICSI**	**IVF**	**FET**	**ET**	**NC**	**NC: males**	**NC: females**	**NC: fertile parents**	**NC: sub-fertile parent**
**Weight - kg**	1.1	0.2	9.6	1.2	54	29	25	x	18	x	x	101	51	50	x	x
	3.0	0.2	14.4	1.8	34	23	11	x	18	x	x	63	33	30	x	x
**Height - cm**	1.1	0.2	75.5	3.3	54	29	25	x	18	x	x	98	51	47	x	x
	3.1	0.2	95.3	5.0	34	23	11	x	18	x	x	62	31	31	x	x
**BMI - kg/m2**	1.1	0.2	13.3	0.6	54	29	25	x	18	x	x	98	51	47	x	x
	3.1	0.2	15.9	1.4	34	23	11	x	18	x	x	61	31	30	x	x
**NINFEA**																
**Cardio-metabolic outcome**	**Age at outcome assessment - y**		**Outcome at assessment - kg, cm etc.**		**Numbers included in the analyses: adjusted models**											
	**Mean**	**SD**	**Mean**	**SD**	**ART**	**ART: males**	**ART: females**	**ICSI**	**IVF**	**FET**	**ET**	**NC**	**NC: males**	**NC: females**	**NC: fertile parents**	**NC: sub-fertile parent**
**Weight - kg**	0.3	0.0	5.7	0.8	253	113	140	165	88	x	x	4762	2440	2322	3400	440
	0.6	0.1	8.0	1.1	270	122	148	177	93	x	x	4990	2541	2449	3560	460
	1.0	0.0	9.7	1.2	219	98	121	153	66	x	x	4443	2267	2176	3157	413
	1.6	0.2	11.4	1.4	241	113	128	165	76	x	x	4761	2428	2333	3392	443
	4.2	0.2	16.7	2.3	147	68	79	97	50	x	x	3778	1935	1843	2704	361

© 2022 Elhakeem A et al. *JAMA Network Open.*

	5.1	0.2	19.0	3.0	42	18	24	31	11	x	x	1076	560	516	770	108
	7.3	0.3	24.4	4.1	77	28	49	52	25	x	x	1940	1012	928	1390	192
	10.2	0.3	34.6	6.8	34	17	17	23	11	x	x	863	448	415	635	86
**Height - cm**	0.3	0.0	60.0	3.4	230	104	126	150	80	x	x	4209	2161	2048	3001	390
	0.6	0.1	68.5	3.7	253	116	137	169	84	x	x	4513	2298	2215	3216	415
	1.0	0.0	76.1	3.8	191	86	105	134	57	x	x	3949	2047	1902	2816	366
	1.6	0.2	82.9	4.2	225	105	120	156	69	x	x	4408	2261	2147	3148	409
	4.2	0.2	103.7	4.8	143	66	77	94	49	x	x	3699	1893	1806	2643	360
	5.1	0.2	111.2	5.9	40	16	24	29	11	x	x	1013	531	482	731	100
	7.3	0.3	124.0	6.3	74	26	48	50	24	x	x	1902	996	906	1360	193
	10.2	0.3	141.5	7.5	35	17	18	23	12	x	x	863	451	412	637	84
**BMI - kg/m2**	0.3	0.0	15.9	1.9	229	103	126	150	79	x	x	4181	2145	2036	2987	386
	0.6	0.1	16.9	1.7	253	116	137	169	84	x	x	4485	2282	2203	3197	413
	1.0	0.0	16.8	1.7	190	85	105	133	57	x	x	3929	2034	1895	2799	365
	1.6	0.2	16.6	1.7	221	103	118	153	68	x	x	4393	2251	2142	3137	408
	4.2	0.2	15.5	1.7	143	66	77	94	49	x	x	3673	1881	1792	2629	356
	5.1	0.2	15.3	1.9	39	16	23	28	11	x	x	971	508	463	701	94
	7.3	0.3	15.9	2.0	74	26	48	50	24	x	x	1876	981	895	1343	189
	10.2	0.3	17.2	2.5	33	16	17	23	10	x	x	846	440	406	624	83
**MoBa**																
**Cardio-metabolic outcome**	**Age at outcome assessment - y**		**Outcome at assessment - kg, cm etc.**		**Numbers included in the analyses: adjusted models**											
	**Mean**	**SD**	**Mean**	**SD**	**ART**	**ART: males**	**ART: females**	**ICSI**	**IVF**	**FET**	**ET**	**NC**	**NC: males**	**NC: females**	**NC: fertile parents**	**NC: sub-fertile parent**
**Weight - kg**	0.1	0.0	5.0	0.7	1,961	991	970	829	1,132	278	1,683	69,347	35,227	34,120	64,609	4,738
	0.3	0.0	6.3	0.8	2,148	1,095	1,053	905	1,243	290	1,858	77,210	39,327	37,883	71,964	5,246
	0.7	0.1	8.8	1.0	1727	884	843	719	1,008	247	1480	58396	29,830	28,566	54264	4132
	1.0	0.1	9.9	1.0	1806	918	888	758	1,048	258	1548	60899	31,095	29,804	56595	4304

© 2022 Elhakeem A et al. *JAMA Network Open.*

	1.3	0.1	10.9	1.1	1442	728	714	606	836	190	1252	49097	25,133	23,964	45719	3378
	1.4	0.1	11.0	1.2	492	275	217	191	301	71	421	14670	7,517	7,153	13551	1119
	2.1	0.2	12.9	1.4	937	489	448	396	541	149	788	29807	15,348	14,459	27541	2266
	3.0	0.1	15.0	1.7	1316	654	662	542	774	189	1127	42365	21,751	20,614	39351	3014
	5.2	0.3	19.9	2.6	1029	511	518	441	588	172	857	34249	17,536	16,713	31888	2361
	7.1	0.2	25.1	3.7	1264	641	623	518	746	183	1081	44397	22,973	21,424	41351	3046
**Height - cm**	0.1	0.0	56.7	2.4	1565	796	769	672	893	240	1325	54811	28,176	26,635	51061	3750
	0.3	0.0	61.9	2.4	2097	1070	1027	877	1,220	280	1817	75841	38,724	37,117	70670	5162
	0.7	0.1	71.3	2.6	1719	874	845	715	1,004	246	1473	58326	29,837	28,489	54203	4123
	1.0	0.1	76.4	2.6	1809	919	890	754	1,055	260	1549	61256	31,315	29,941	56904	4352
	1.3	0.1	80.4	2.8	1416	713	703	591	825	185	1231	48656	24,934	23,722	45287	3369
	1.4	0.1	81.6	3.2	486	265	221	186	300	72	414	14593	7,477	7,116	13480	1113
	2.1	0.2	88.6	3.4	943	485	458	394	549	152	791	29970	15,340	14,630	27687	2283
	3.0	0.1	96.5	3.7	1280	640	640	525	755	186	1094	41341	21,159	20,182	38394	2947
	5.2	0.3	113.0	4.9	1042	520	522	440	602	170	872	34679	17,700	16,979	32285	2394
	7.1	0.2	16.0	5.2	1292	654	638	532	760	187	1105	45507	23,445	22,062	42364	3143
**BMI - kg/m2**	0.1	0.0	15.5	1.4	1556	790	766	667	889	236	1320	54263	27,740	26,523	50545	3718
	0.3	0.0	16.5	1.4	2083	1063	1020	875	1,208	278	1805	75106	38,245	36,861	70009	5097
	0.7	0.1	17.3	1.4	1689	868	821	704	985	242	1447	57342	29,288	28,054	53295	4047
	1.0	0.1	17.0	1.3	1777	906	871	743	1,034	255	1522	60074	30,681	29,393	55822	4252
	1.3	0.1	16.8	1.3	1398	705	693	586	812	183	1215	47486	24,341	23,145	44207	3279
	1.4	0.1	16.6	1.3	471	263	208	181	290	71	400	14190	7,273	6,917	13104	1086
	2.1	0.2	16.4	1.3	895	463	432	381	514	144	751	28809	14,789	14,020	26604	2205
	3.0	0.1	16.1	1.4	1233	621	612	505	728	178	1055	39431	20,253	19,178	36631	2800
	5.2	0.3	15.5	1.5	1006	502	504	429	577	168	840	33142	16,967	16,175	30850	2292
	7.1	0.2	15.8	1.7	1236	627	609	502	734	180	1056	43087	22,294	20,793	40126	2961
**Piccolipiù**																

© 2022 Elhakeem A et al. *JAMA Network Open.*

Cardio-metabolic outcome	Age at outcome assessment - y		Outcome at assessment - kg, cm etc.		Numbers included in the analyses: adjusted models											
	Mean	SD	Mean	SD	ART	ART: males	ART: females	ICSI	IVF	FET	ET	NC	NC: males	NC: females	NC: fertile parents	NC: sub-fertile parent
**Weight - kg**	0.1	0.0	4.0	0.7	86	38	48	x	x	x	x	2479	1287	1192	2231	251
	0.3	0.0	5.9	0.8	83	35	48	x	x	x	x	2299	1171	1128	2026	241
	0.5	0.0	7.7	0.9	74	31	43	x	x	x	x	2045	1049	996	1829	220
	1.0	0.0	9.9	1.1	62	25	37	x	x	x	x	1722	870	852	1542	181
	1.5	0.0	11.2	1.3	62	28	34	x	x	x	x	1717	880	837	1534	184
	2.0	0.0	12.6	1.5	79	37	42	x	x	x	x	1912	988	924	1713	201
	4.4	0.3	17.8	2.2	49	19	30	x	x	x	x	1745	885	860	1590	157
**Height - cm**	0.1	0.0	53.7	2.7	80	36	44	x	x	x	x	2184	1122	1062	1962	225
	0.3	0.0	60.7	2.8	75	31	44	x	x	x	x	2061	1059	1002	1831	214
	0.5	0.0	67.8	3.0	65	27	38	x	x	x	x	1782	913	869	1598	188
	1.0	0.0	76.2	3.4	55	22	33	x	x	x	x	1535	779	756	1374	162
	1.5	0.0	82.2	3.7	59	27	32	x	x	x	x	1601	823	778	1424	178
	2.0	0.0	97.8	3.9	72	34	38	x	x	x	x	1733	899	834	1549	186
	4.4	0.3	105.5	4.4	49	19	30	x	x	x	x	1737	881	856	1584	155
**BMI - kg/m2**	0.1	0.0	14.0	1.6	79	35	44	x	x	x	x	2179	1120	1059	1958	224
	0.3	0.0	16.0	1.7	75	31	44	x	x	x	x	2058	1056	1002	1848	214
	0.5	0.0	16.8	1.7	65	27	38	x	x	x	x	1775	908	867	1591	188
	1.0	0.0	17.0	1.7	55	22	33	x	x	x	x	1520	773	747	1360	161
	1.5	0.0	16.6	1.6	59	27	32	x	x	x	x	1589	814	775	1413	177
	2.0	0.0	16.4	1.6	71	34	37	x	x	x	x	1717	891	826	1537	182
	4.4	0.3	16.0	1.4	49	19	30	x	x	x	x	1735	880	855	1582	155
**Waist circumference - cm**	4.4	0.3	54.0	3.8	46	18	28	x	x	x	x	1593	809	784	1448	147

© 2022 Elhakeem A et al. *JAMA Network Open.*

SWS																
**Cardio-metabolic outcome**	**Age at assessment - y**		**Outcome at assessment - kg, cm etc.**		**Numbers included in the analyses: adjusted models**											
	**Mean**	**SD**	**Mean**	**SD**	**ART**	**ART: males**	**ART: females**	**ICSI**	**IVF**	**FET**	**ET**	**NC**	**NC: males**	**NC: females**	**NC: fertile parents**	**NC: sub-fertile parent**
**Weight - kg**	0.5	0.0	8.0	1.0	25	15	10	x	x	x	x	2154	1126	1028	x	x
	1.0	0.1	10.1	1.2	35	19	16	x	x	x	x	2554	1315	1239	x	x
	2.0	0.1	12.6	1.5	34	18	16	x	x	x	x	2424	1230	1194	x	x
	3.1	0.1	15.0	1.8	34	19	15	x	x	x	x	2323	1184	1139	x	x
	4.1	0.1	17.3	2.3	18	11	7	x	x	x	x	1101	568	533	x	x
	6.7	0.3	23.5	3.9	28	16	12	x	x	x	x	1822	925	897	x	x
	9.2	0.3	31.1	6.4	19	10	9	x	x	x	x	1122	552	570	x	x
**Height - cm**	0.5	0.0	67.4	2.6	24	14	10	x	x	x	x	2136	1120	1016	x	x
	1.0	0.1	75.8	2.9	36	19	17	x	x	x	x	2474	1283	1191	x	x
	2.0	0.1	86.5	3.2	34	18	16	x	x	x	x	2345	1195	1150	x	x
	3.1	0.1	95.7	3.6	33	19	14	x	x	x	x	2291	1163	1128	x	x
	4.1	0.1	104.0	4.0	18	11	7	x	x	x	x	1100	565	535	x	x
	6.7	0.3	120.6	5.3	28	16	12	x	x	x	x	1823	924	899	x	x
	9.2	0.3	135.3	6.0	19	10	9	x	x	x	x	1125	555	570	x	x
**BMI - kg/m2**	0.5	0.0	17.6	1.5	24	14	10	x	x	x	x	2132	1117	1015	x	x
	1.0	0.1	17.5	1.5	35	19	16	x	x	x	x	2465	1281	1184	x	x
	2.0	0.1	16.8	1.4	34	18	16	x	x	x	x	2331	1186	1145	x	x
	3.1	0.1	16.3	1.4	33	19	14	x	x	x	x	2280	1156	1124	x	x
	4.1	0.1	16.0	1.4	18	11	7	x	x	x	x	1097	565	532	x	x
	6.7	0.3	16.1	1.9	28	16	12	x	x	x	x	1818	923	895	x	x
	9.2	0.3	16.9	2.7	19	10	9	x	x	x	x	1121	552	569	x	x
	2.0	0.1	49.0	2.9	32	18	14	x	x	x	x	2225	1122	1103	x	x

**Waist circumference - cm**	6.7	0.3	56.1	4.8	28	16	12	x	x	x	x	1809	917	892	x	x
	9.2	0.3	61.9	6.9	19	10	9	x	x	x	x	1121	553	568	x	x
**Body fat %**	9.2	0.3	21.3	5.5	14	7	7	x	x	x	x	796	398	398	x	x
**Fat mass index kg/m2**	4.1	0.1	4.5	1.1	15	8	7	x	x	x	x	796	405	391	x	x
	6.7	0.3	5.0	2.1	10	6	4	x	x	x	x	733	367	366	x	x
	9.2	0.3	3.7	1.6	14	7	7	x	x	x	x	796	398	398	x	x
**HUNT**																
**Cardio-metabolic outcome**	**Age at outcome assessment - y**		**Outcome at assessment - kg, cm etc.**		**Numbers included in the analyses: adjusted models**											
	**Mean**	**SD**	**Mean**	**SD**	**ART**	**ART: males**	**ART: females**	**ICSI**	**IVF**	**FET**	**ET**	**NC**	**NC: males**	**NC: females**	**NC: fertile parents**	**NC: sub-fertile parent**
**Weight - kg**	15.0	1.1	61.4	13.5	121	48	73	30	91	10	111	9,711	4,886	4,825	x	x
	18.1	0.7	70.7	14.7	42	21	21	14	28	2	40	7,281	2,113	2,357	x	x
**Height - cm**	15.0	1.1	168.2	8.8	121	48	73	30	91	10	111	9,737	4,887	4,850	x	x
	18.1	0.7	173.0	9.2	42	21	21	14	28	2	40	7,287	2,113	2,363	x	x
**BMI - kg/m2**	15.0	1.1	21.6	3.8	121	48	73	30	91	10	111	9,704	4,884	4,820	x	x
	18.1	0.7	23.6	4.2	42	21	21	14	28	2	40	7,278	2,112	2,355	x	x
**Waist circumference - cm**	15.0	1.1	77.6	11.0	121	48	73	30	91	10	111	9,730	4,883	4,847	x	x
	18.1	0.7	84.1	12.2	42	21	21	14	28	2	40	7,253	2,105	2,358	x	x

© 2022 Elhakeem A et al. *JAMA Network Open.*

eFigure 1. Directed Acyclic Graph Used to Identify Potential Confounders

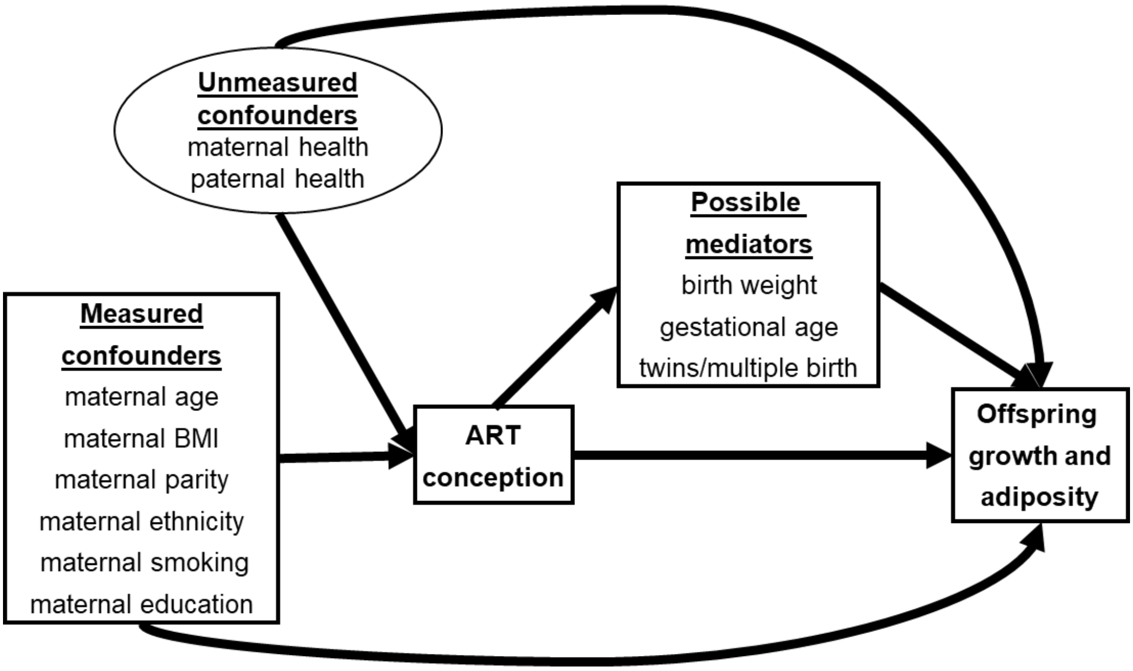

© 2022 Elhakeem A et al. *JAMA Network Open.*

eFigure 2. Cohort-Specific Mean Differences in Length / Height Between Offspring Conceived via ART and Those Who Were NC

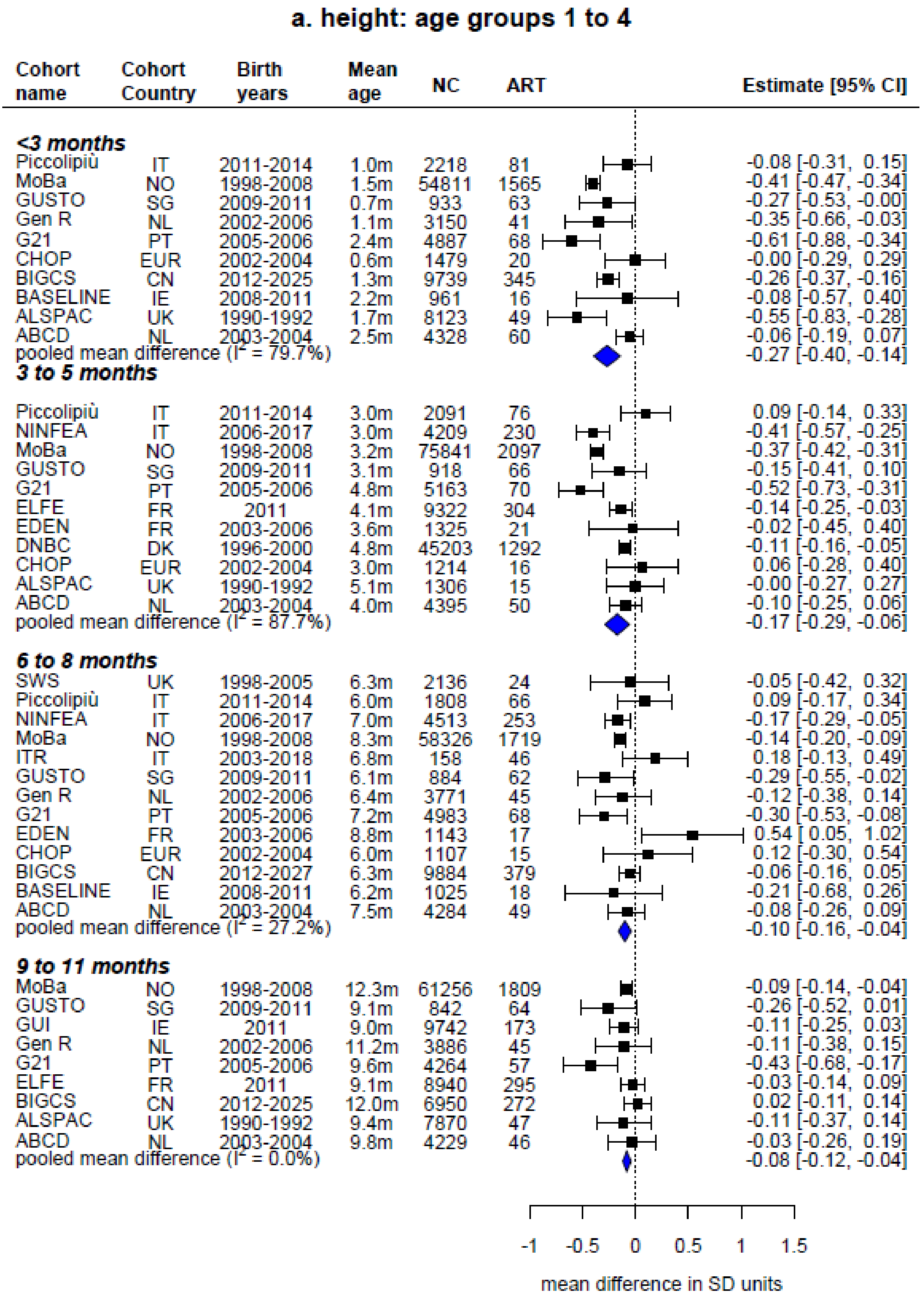

© 2022 Elhakeem A et al. *JAMA Network Open.*

b. height: age groups 5 to 7

Cohort name	Cohort Country	Birth years	Mean age	NC	ART	Estimate [95% CI]
12 to 16 months						
Piccolipiù	IT	2011-2014	12.0m	1558	55	0.09 [-0.19, 0.37]
NINFEA	IT	2006-2017	12.0m	3949	191	-0.23 [-0.39, -0.07]
DNBC	DK	1996-2000	12.0m	42116	1211	-0.04 [-0.10, 0.01]
CHOP	EUR	2002-2004	12.0m	1004	15	0.24 [-0.18, 0.65]
GUSTO	SG	2009-2011	12.1m	862	62	-0.35 [-0.62, -0.09]
ELFE	FR	2011	12.2m	7866	266	0.01 [-0.11, 0.13]
EDEN	FR	2003-2006	12.2m	1361	21	0.21 [-0.21, 0.64]
SWS	UK	1998-2005	12.6m	2474	36	-0.24 [-0.55, 0.07]
BASELINE	IE	2008-2011	12.6m	963	19	-0.13 [-0.58, 0.33]
BIS	AU	2010-2013	13.0m	665	35	0.02 [-0.39, 0.44]
ABCD	NL	2003-2004	13.1m	4082	49	0.05 [-0.22, 0.33]
AOF	CA	2008-2011	13.2m	1296	35	0.06 [-0.28, 0.40]
MUBICOS	IT	2009-2015	13.2m	98	60	0.26 [-0.19, 0.72]
MoBa	NO	1998-2008	16.0m	48656	1416	-0.06 [-0.12, -0.01]
GASPII	IT	2003-2004	16.2m	545	7	0.10 [-0.71, 0.91]
G21	PT	2005-2006	14.4m	4996	72	-0.29 [-0.49, -0.09]
MoBa	NO	1998-2008	17.3m	14593	486	-0.18 [-0.27, -0.08]
pooled mean difference (I^2 = 48.3%)						-0.06 [-0.13, 0.00]
17 to 23 months						
Piccolipiù	IT	2011-2014	18.0m	1619	59	0.19 [-0.08, 0.46]
ABCD	NL	2003-2004	18.1m	3919	48	0.11 [-0.19, 0.41]
GUSTO	SG	2009-2011	18.4m	777	50	-0.32 [-0.61, -0.04]
NINFEA	IT	2006-2017	19.3m	4408	225	-0.15 [-0.29, -0.02]
ALSPAC	UK	1990-1992	20.4m	7238	44	-0.04 [-0.45, 0.37]
ITR	IT	2003-2018	17.4m	232	54	-0.04 [-0.32, 0.24]
G21	PT	2005-2006	20.4m	4904	68	-0.27 [-0.44, -0.10]
pooled mean difference (I^2 = 63.4%)						-0.12 [-0.25, -0.00]
2 years						
AOF	CA	2008-2011	2.0y	1358	41	0.22 [0.02, 0.42]
ELFE	FR	2011	2.0y	6745	227	0.03 [-0.10, 0.16]
EDEN	FR	2003-2006	2.0y	1156	17	0.42 [-0.06, 0.91]
GUSTO	SG	2009-2011	2.0y	801	56	-0.41 [-0.68, -0.13]
GUiNZ	NZ	2009-2010	2.0y	3640	151	-0.12 [-0.30, 0.05]
SWS	UK	1998-2005	2.0y	2345	34	-0.22 [-0.51, 0.07]
Piccolipiù	IT	2011-2014	2.0y	1756	72	0.07 [-0.18, 0.31]
Gen R	NL	2002-2006	2.1y	3439	40	-0.31 [-0.61, -0.01]
ABCD	NL	2003-2004	2.1y	3813	45	-0.01 [-0.32, 0.29]
BASELINE	IE	2008-2011	2.2y	1014	20	-0.09 [-0.52, 0.33]
MoBa	NO	1998-2008	2.2y	29970	943	-0.07 [-0.14, 0.00]
ITR	IT	2003-2018	2.5y	190	54	0.30 [-0.06, 0.65]
BIGCS	CN	2012-2027	2.8y	6085	268	-0.03 [-0.15, 0.10]
G21	PT	2005-2006	2.6y	4769	69	-0.20 [-0.36, -0.04]
pooled mean difference (I^2 = 68.4%)						-0.05 [-0.14, 0.05]

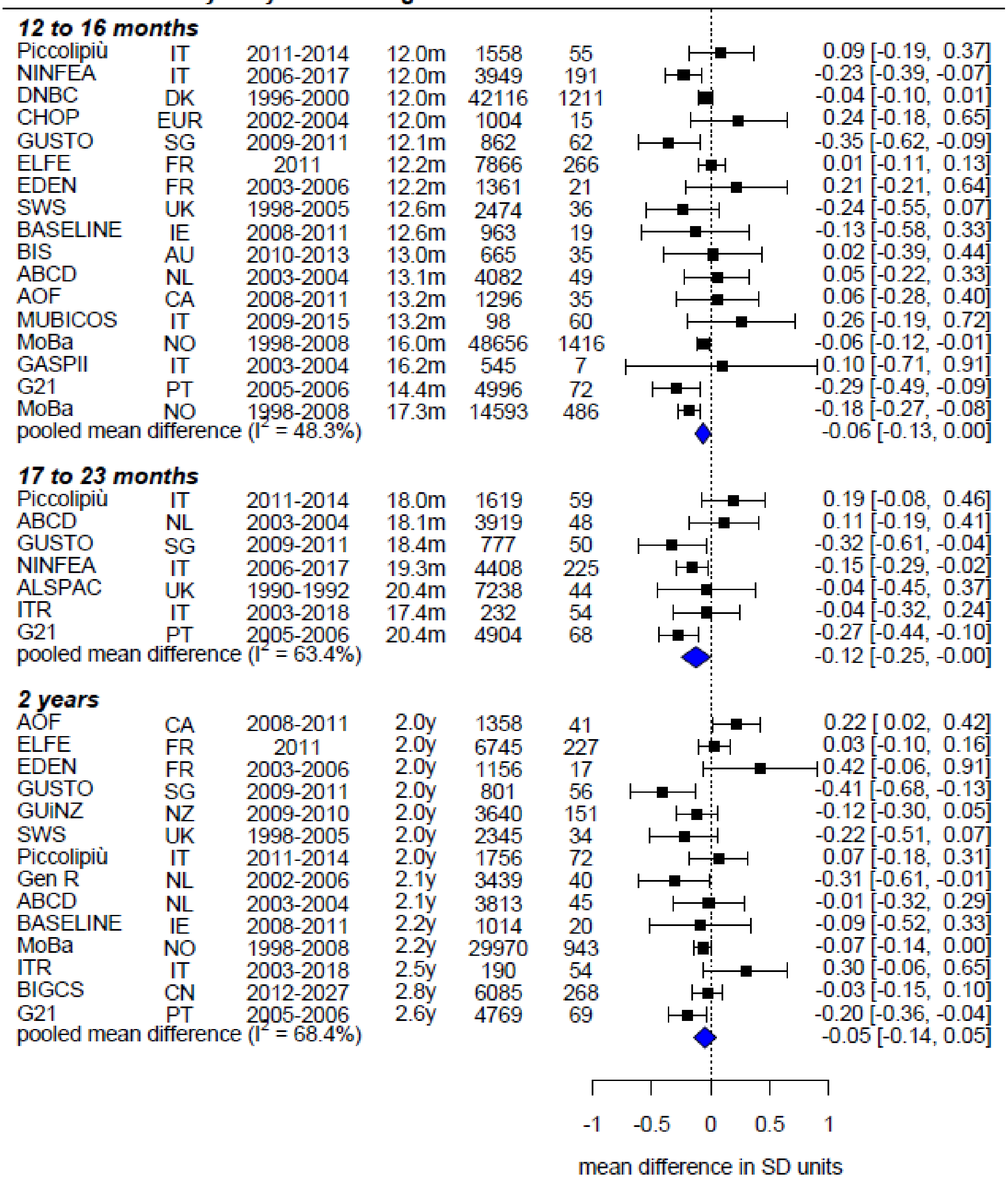

© 2022 Elhakeem A et al. *JAMA Network Open.*

c. height: age groups 8 and 9

Cohort name	Cohort Country	Birth years	Mean age	NC	ART	Estimate [95% CI]
3 years						
AOF	CA	2008-2011	3.0y	1780	37	0.03 [-0.25, 0.32]
ELFE	FR	2011	3.0y	2217	89	0.03 [-0.18, 0.23]
GUI	IE	2011	3.0y	8480	158	0.01 [-0.16, 0.17]
GUSTO	SG	2009-2011	3.0y	835	59	-0.37 [-0.64, -0.10]
MUBICOS	IT	2009-2015	3.0y	62	39	-0.17 [-0.59, 0.25]
SWS	UK	1998-2005	3.1y	2291	33	-0.31 [-0.59, -0.03]
MoBa	NO	1998-2008	3.1y	41341	1280	-0.05 [-0.11, 0.02]
MCS	UK	2000-2002	3.1y	2132	30	-0.23 [-0.60, 0.14]
EDEN	FR	2003-2006	3.2y	1091	19	0.49 [0.03, 0.95]
ITR	IT	2003-2018	3.5y	186	44	0.16 [-0.24, 0.56]
ALSPAC	UK	1990-1992	3.7y	6580	37	-0.14 [-0.44, 0.17]
NINFEA	IT	2006-2017	4.2y	3699	143	-0.02 [-0.19, 0.14]
G21	PT	2005-2006	3.5y	4350	68	-0.23 [-0.42, -0.04]
pooled mean difference (I^2 = 36.2%)						-0.08 [-0.15, 0.00]
4 to 5 years						
SWS	UK	1998-2005	4.1y	1100	18	-0.07 [-0.45, 0.30]
GASPII	IT	2003-2004	4.1y	528	8	-0.20 [-0.82, 0.42]
BIS	EUR	2010-2013	4.2y	443	25	-0.17 [-0.57, 0.22]
Piccolipiù	IT	2011-2014	4.4y	1765	49	0.06 [-0.21, 0.33]
G21	PT	2005-2006	4.4y	5150	89	-0.21 [-0.41, -0.02]
GUiNZ	NZ	2009-2010	4.5y	4274	173	-0.11 [-0.27, 0.04]
GUSTO	SG	2009-2011	4.6y	807	61	-0.38 [-0.64, -0.11]
NINFEA	IT	2006-2017	5.1y	1013	40	-0.17 [-0.49, 0.15]
BASELINE	IE	2008-2011	5.1y	812	13	-0.07 [-0.61, 0.47]
GUI	IE	2011	5.2y	7864	151	0.01 [-0.16, 0.19]
AOF	CA	2008-2011	5.2y	1731	41	0.18 [-0.07, 0.44]
MCS	UK	2000-2002	5.2y	2060	30	0.13 [-0.22, 0.49]
MoBa	NO	1998-2008	5.3y	34679	1042	-0.05 [-0.11, 0.02]
ITR	IT	2003-2018	5.5y	144	46	0.13 [-0.27, 0.54]
EDEN	FR	2003-2006	5.7y	981	14	0.47 [-0.06, 1.00]
ABCD	NL	2003-2004	5.7y	3598	35	0.02 [-0.30, 0.35]
pooled mean difference (I^2 = 19.8%)						-0.05 [-0.12, 0.01]

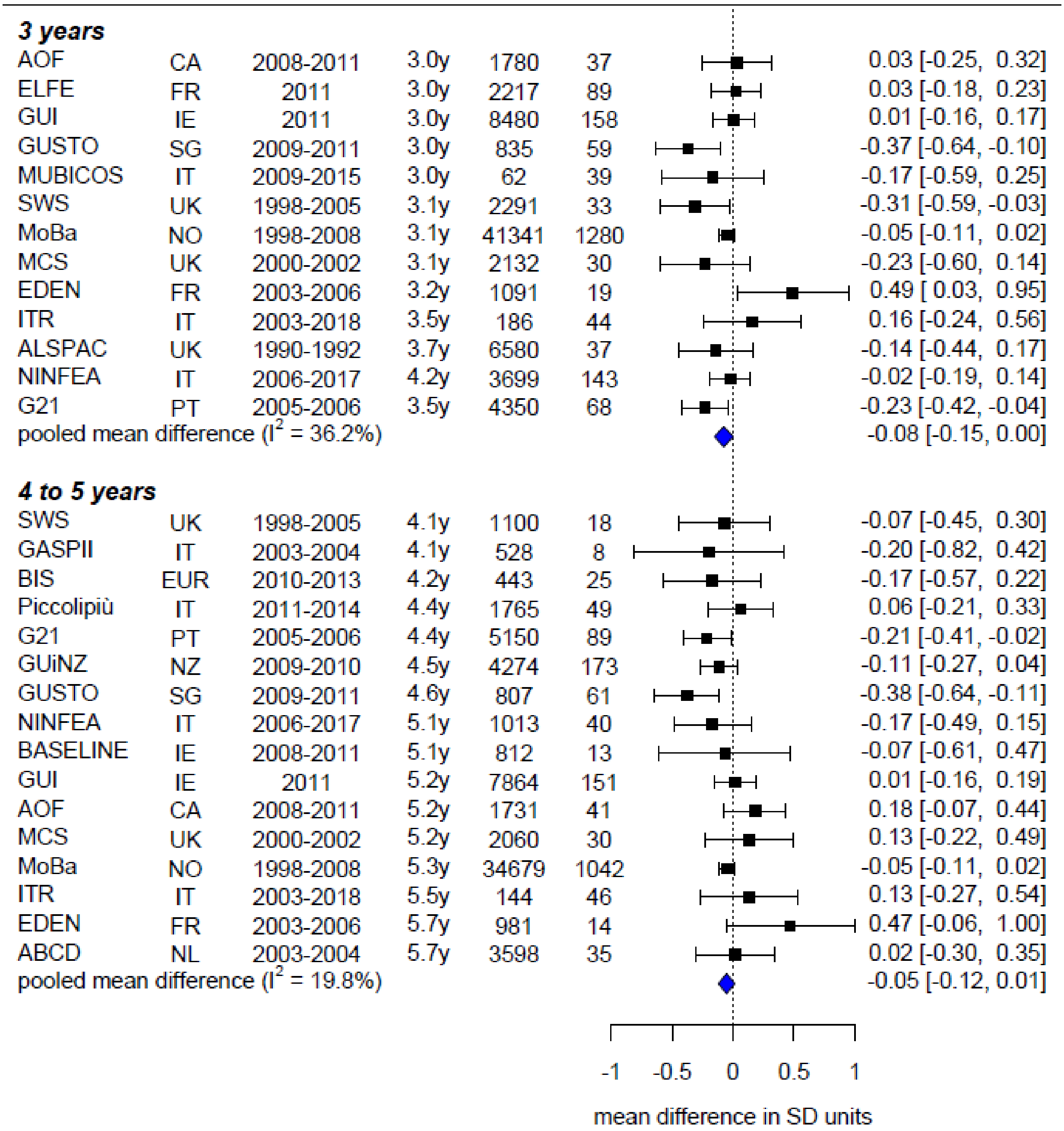

© 2022 Elhakeem A et al. *JAMA Network Open.*

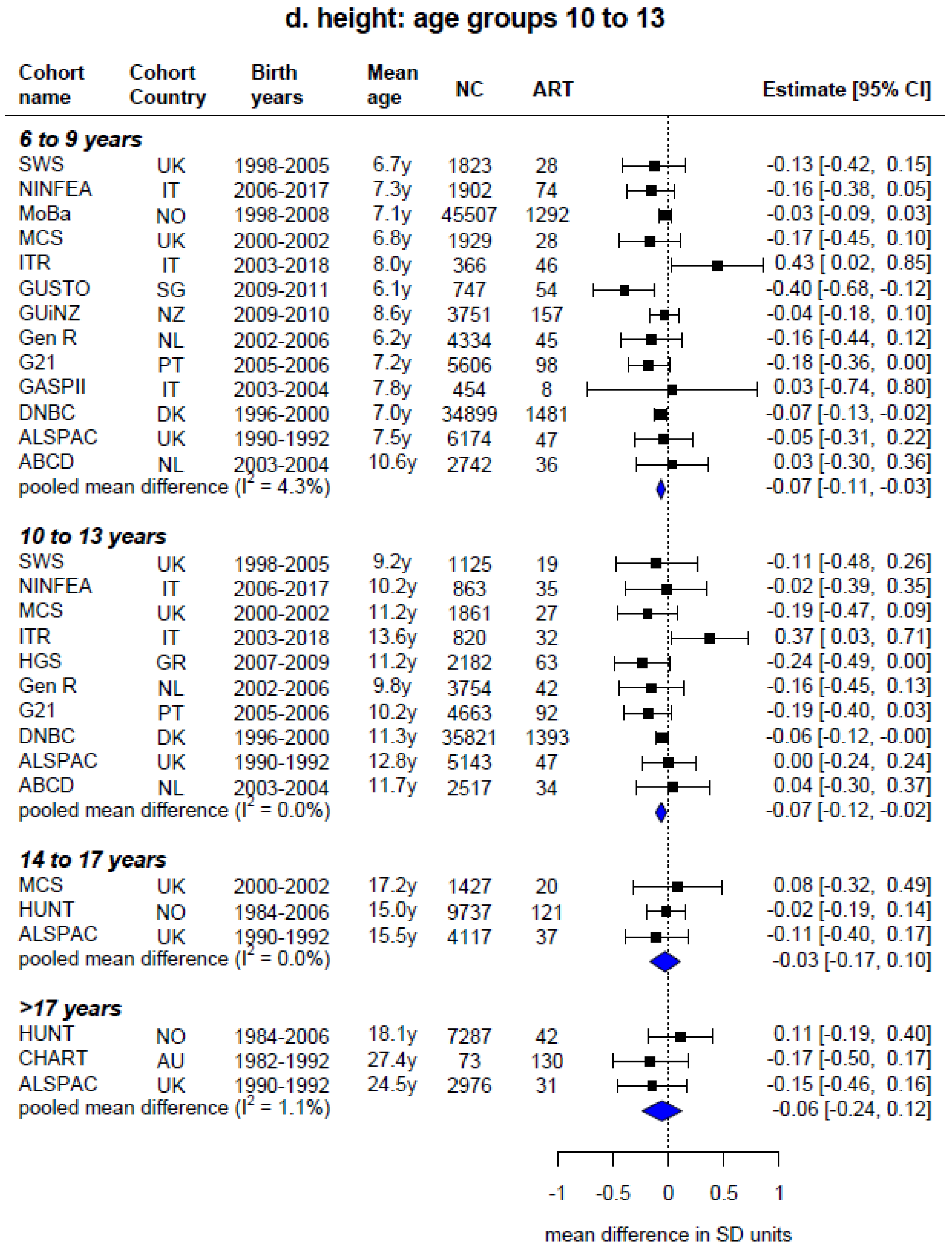

Estimates represent the cohort-specifc confounder-adjusted mean differences in SD units [and 95% confidence intervals] in length/height at each age group between ART-conceived and NC offspring (ART minus NC). Estimates were adjusted (as fully as possible) for maternal age, parity, BMI, smoking, education, ethnicity (or country of birth), plus offspring sex and age at outcome assessment. Cohorts are arranged by the offspring's mean age at outcome assessment. Blue diamonds represent the pooled mean differences from random-effects meta-analyses. NC is the number of NC ofspring; ART is the number of ART-conceived offspring; I² represents the percentage of total variability due to between cohort heterogeneity.

eFigure 3. Cohort-Specific Mean Differences in Weight Between Offspring Conceived via ART and Those Who Were NC

a. weight: age groups 1 to 4

Cohort name	Cohort Country	Birth years	Mean age	NC	ART	Estimate [95% CI]
<3 months						
CHOP	EUR	2002-2004	0.6m	1479	20	-0.10 [-0.36, 0.15]
GUSTO	SG	2009-2011	0.7m	935	63	-0.17 [-0.43, 0.08]
Piccolipiù	IT	2011-2014	1.0m	2479	86	-0.09 [-0.30, 0.13]
Gen R	NL	2002-2006	1.1m	3788	47	-0.40 [-0.71, -0.09]
BIGCS	CN	2012-2025	1.3m	10147	389	-0.22 [-0.32, -0.12]
MoBa	NO	1998-2008	1.5m	69347	1961	-0.42 [-0.48, -0.37]
ALSPAC	UK	1990-1992	1.7m	8599	53	-0.45 [-0.71, -0.19]
BASELINE	IE	2008-2011	2.2m	961	16	-0.34 [-0.82, 0.15]
ABCD	NL	2003-2004	2.5m	4449	61	-0.06 [-0.17, 0.05]
G21	PT	2005-2006	2.4m	4887	68	-0.58 [-0.80, -0.35]
pooled mean difference (I^2 = 80.7%)						-0.27 [-0.39, -0.16]
3 to 5 months						
CHOP	EUR	2002-2004	3.0m	1217	16	-0.02 [-0.39, 0.34]
NINFEA	IT	2006-2017	3.0m	4762	253	-0.37 [-0.51, -0.23]
Piccolipiù	IT	2011-2014	3.0m	2299	83	-0.09 [-0.31, 0.14]
GUSTO	SG	2009-2011	3.1m	918	66	-0.20 [-0.46, 0.06]
MoBa	NO	1998-2008	3.2m	77210	2148	-0.34 [-0.39, -0.29]
EDEN	FR	2003-2006	3.6m	1326	22	-0.10 [-0.52, 0.31]
ABCD	NL	2003-2004	4.0m	4701	55	-0.15 [-0.30, -0.00]
ELFE	FR	2011	4.1m	9632	309	-0.11 [-0.22, 0.00]
DNBC	DK	1996-2000	4.8m	45203	1292	-0.08 [-0.14, -0.03]
ALSPAC	UK	1990-1992	5.1m	1306	15	-0.14 [-0.38, 0.11]
G21	PT	2005-2006	4.8m	5163	70	-0.46 [-0.66, -0.26]
pooled mean difference (I^2 = 79.0%)						-0.20 [-0.29, -0.11]
6 to 8 months						
CHOP	EUR	2002-2004	6.0m	1107	15	0.09 [-0.32, 0.50]
Piccolipiù	IT	2011-2014	6.0m	2045	74	0.10 [-0.14, 0.33]
GUSTO	SG	2009-2011	6.1m	880	62	-0.30 [-0.57, -0.02]
BASELINE	IE	2008-2011	6.2m	1026	18	-0.42 [-0.89, 0.05]
BIGCS	CN	2012-2027	6.3m	6296	278	0.00 [-0.10, 0.11]
SWS	UK	1998-2005	6.3m	2154	25	-0.07 [-0.45, 0.32]
Gen R	NL	2002-2006	6.4m	4256	51	-0.10 [-0.34, 0.15]
ITR	IT	2003-2018	6.8m	166	50	0.02 [-0.18, 0.23]
NINFEA	IT	2006-2017	7.0m	4990	270	-0.22 [-0.34, -0.10]
ABCD	NL	2003-2004	7.5m	4388	51	-0.15 [-0.34, 0.04]
MoBa	NO	1998-2008	8.3m	58396	1727	-0.12 [-0.17, -0.07]
EDEN	FR	2003-2006	8.8m	1171	17	0.71 [0.23, 1.20]
G21	PT	2005-2006	7.2m	4983	68	-0.32 [-0.51, -0.12]
pooled mean difference (I^2 = 67.0%)						-0.09 [-0.18, -0.00]
9 to 11 months						
GUI	IE	2011	9.0m	9742	173	-0.11 [-0.26, 0.05]
ELFE	FR	2011	9.1m	9246	302	-0.04 [-0.15, 0.07]
GUSTO	SG	2009-2011	9.1m	841	64	-0.21 [-0.48, 0.06]
ALSPAC	UK	1990-1992	9.4m	7920	47	-0.27 [-0.58, 0.03]
ABCD	NL	2003-2004	9.8m	4319	49	-0.10 [-0.33, 0.13]
Gen R	NL	2002-2006	11.2m	3891	45	-0.04 [-0.31, 0.22]
BIGCS	CN	2012-2025	12.0m	9875	375	0.14 [0.01, 0.26]
MoBa	NO	1998-2008	12.3m	60899	1806	-0.09 [-0.15, -0.04]
G21	PT	2005-2006	9.6m	4264	57	-0.37 [-0.63, -0.12]
pooled mean difference (I^2 = 66.5%)						-0.09 [-0.18, 0.00]

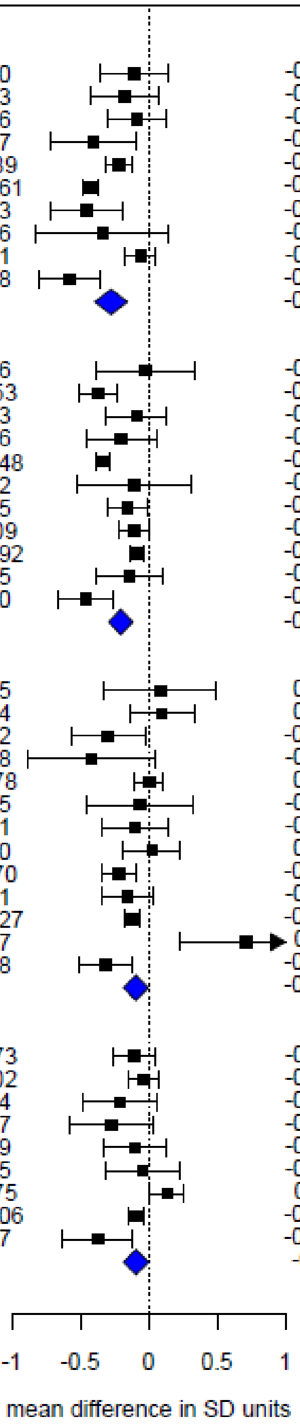

© 2022 Elhakeem A et al. *JAMA Network Open.*

b. weight: age groups 5 to 7

Cohort name	Cohort Country	Birth years	Mean age	NC	ART	Estimate [95% CI]
12 to 16 months						
CHOP	EUR	2002-2004	12.0m	1003	15	0.14 [-0.34, 0.62]
DNBC	DK	1996-2000	12.0m	42116	1211	-0.02 [-0.07, 0.04]
NINFEA	IT	2006-2017	12.0m	4443	219	-0.21 [-0.35, -0.06]
Piccolipiù	IT	2011-2014	12.0m	1722	62	-0.04 [-0.30, 0.22]
GUSTO	SG	2009-2011	12.1m	862	62	-0.16 [-0.43, 0.12]
EDEN	FR	2003-2006	12.2m	1365	21	0.30 [-0.14, 0.74]
ELFE	FR	2011	12.2m	8112	271	-0.06 [-0.18, 0.06]
BASELINE	IE	2008-2011	12.6m	961	19	-0.30 [-0.76, 0.16]
SWS	UK	1998-2005	12.6m	2554	35	-0.10 [-0.42, 0.23]
BIS	AU	2010-2013	13.0m	673	35	-0.11 [-0.43, 0.22]
ABCD	NL	2003-2004	13.1m	4168	51	-0.08 [-0.34, 0.19]
AOF	CA	2008-2011	13.2m	1386	36	-0.10 [-0.40, 0.19]
MUBICOS	IT	2009-2015	13.2m	101	54	-0.33 [-0.75, 0.08]
MoBa	NO	1998-2008	16.0m	49097	1442	-0.09 [-0.15, -0.04]
GASPII	IT	2003-2004	16.2m	554	8	-0.23 [-1.16, 0.70]
G21	PT	2005-2006	14.4m	4996	72	-0.36 [-0.57, -0.15]
MoBa	NO	1998-2008	17.3m	14670	492	-0.15 [-0.24, -0.05]
pooled mean difference (I^2 = 37.0%)						-0.10 [-0.16, -0.04]
17 to 23 months						
Piccolipiù	IT	2011-2014	18.0m	1717	62	0.02 [-0.23, 0.28]
ABCD	NL	2003-2004	18.1m	4021	48	0.04 [-0.26, 0.34]
GUSTO	SG	2009-2011	18.4m	820	55	-0.23 [-0.51, 0.06]
NINFEA	IT	2006-2017	19.3m	4761	241	-0.19 [-0.32, -0.06]
ALSPAC	UK	1990-1992	20.4m	7296	45	-0.24 [-0.53, 0.05]
G21	PT	2005-2006	20.4m	4904	68	-0.29 [-0.49, -0.08]
ITR	IT	2003-2018	17.4m	237	53	-0.07 [-0.39, 0.26]
pooled mean difference (I^2 = 0.0%)						-0.16 [-0.22, -0.09]
2 years						
AOF	CA	2008-2011	2.0y	1406	41	0.06 [-0.26, 0.37]
EDEN	FR	2003-2006	2.0y	1163	17	0.36 [-0.12, 0.83]
ELFE	FR	2011	2.0y	6827	230	-0.02 [-0.15, 0.11]
GUiNZ	NZ	2009-2010	2.0y	3643	151	-0.04 [-0.17, 0.10]
GUSTO	SG	2009-2011	2.0y	832	58	-0.32 [-0.60, -0.04]
Piccolipiù	IT	2011-2014	2.0y	1912	79	0.06 [-0.17, 0.29]
SWS	UK	1998-2005	2.0y	2424	34	-0.12 [-0.40, 0.16]
ABCD	NL	2003-2004	2.1y	3874	47	-0.00 [-0.30, 0.30]
Gen R	NL	2002-2006	2.1y	3494	40	-0.12 [-0.41, 0.17]
BASELINE	IE	2008-2011	2.2y	1031	20	-0.22 [-0.66, 0.22]
MoBa	NO	1998-2008	2.2y	29807	937	-0.14 [-0.21, -0.07]
ITR	IT	2003-2018	2.5y	194	54	0.29 [-0.09, 0.67]
BIGCS	CN	2012-2027	2.8y	6133	267	-0.07 [-0.19, 0.05]
G21	PT	2005-2006	2.6y	4769	69	-0.31 [-0.50, -0.12]
pooled mean difference (I^2 = 32.2%)						-0.08 [-0.14, -0.01]

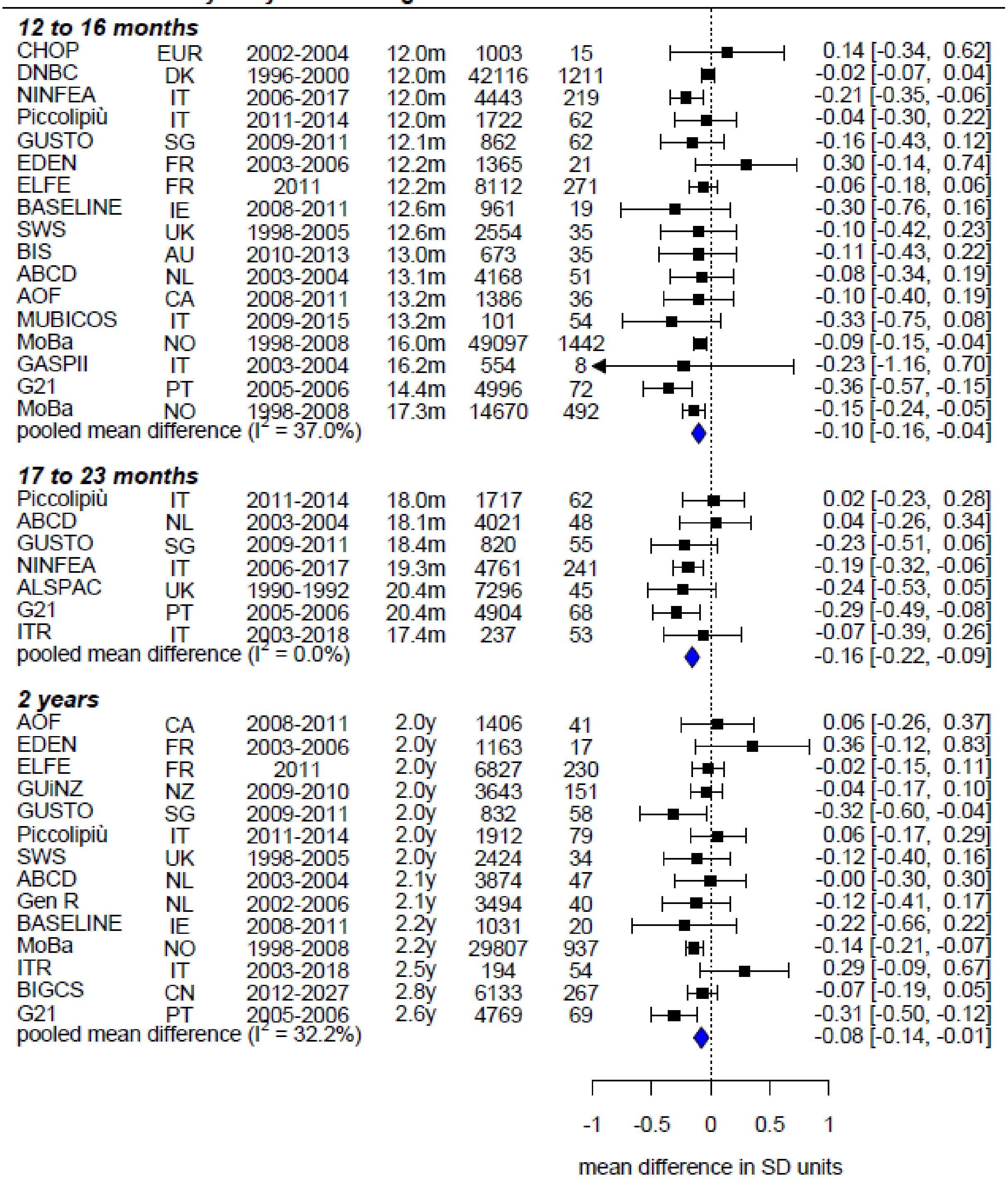

© 2022 Elhakeem A et al. *JAMA Network Open.*

c. weight: age groups 8 and 9

Cohort name	Cohort Country	Birth years	Mean age	NC	ART	Estimate [95% CI]
3 years						
AOF	CA	2008-2011	3.0y	1811	37	0.17 [-0.17, 0.51]
ELFE	FR	2011	3.0y	2228	89	-0.02 [-0.23, 0.20]
GUI	IE	2011	3.0y	8480	158	-0.07 [-0.23, 0.09]
GUSTO	SG	2009-2011	3.0y	839	59	-0.22 [-0.49, 0.05]
MUBICOS	IT	2009-2015	3.0y	63	34	-0.42 [-0.83, -0.01]
MCS	UK	2000-2002	3.1y	2153	30	-0.32 [-0.68, 0.04]
MoBa	NO	1998-2008	3.1y	42365	1316	-0.11 [-0.17, -0.05]
SWS	UK	1998-2005	3.1y	2323	34	-0.13 [-0.35, 0.10]
EDEN	FR	2003-2006	3.2y	1091	19	0.38 [-0.08, 0.83]
ITR	IT	2003-2018	3.5y	188	44	0.02 [-0.32, 0.36]
ALSPAC	UK	1990-1992	3.7y	6167	37	-0.45 [-0.77, -0.12]
NINFEA	IT	2006-2017	4.2y	3778	147	-0.02 [-0.19, 0.16]
G21	PT	2005-2006	3.5y	4350	68	-0.24 [-0.43, -0.04]
pooled mean difference (I^2 = 0.0%)						-0.11 [-0.16, -0.07]
4 to 5 years						
GASPII	IT	2003-2004	4.1y	528	8	-0.24 [-1.08, 0.59]
SWS	UK	1998-2005	4.1y	1101	18	-0.06 [-0.46, 0.34]
BIS	AU	2010-2013	4.2y	447	25	-0.04 [-0.46, 0.37]
G21	PT	2005-2006	4.4y	5158	89	-0.26 [-0.42, -0.09]
Piccolipiù	IT	2011-2014	4.4y	1745	49	-0.03 [-0.31, 0.25]
GUiNZ	NZ	2009-2010	4.5y	4274	173	-0.09 [-0.21, 0.03]
GUSTO	SG	2009-2011	4.6y	805	61	-0.25 [-0.52, 0.02]
BASELINE	IE	2008-2011	5.1y	813	13	-0.37 [-0.90, 0.17]
NINFEA	IT	2006-2017	5.1y	1076	42	-0.09 [-0.37, 0.18]
AOF	CA	2008-2011	5.2y	1763	41	0.11 [-0.17, 0.39]
GUI	IE	2011	5.2y	7864	151	-0.04 [-0.20, 0.12]
MCS	UK	2000-2002	5.2y	2060	30	0.05 [-0.43, 0.53]
MoBa	NO	1998-2008	5.3y	34249	1029	-0.12 [-0.18, -0.05]
ITR	IT	2003-2018	5.5y	143	46	-0.38 [-0.76, -0.01]
ABCD	NL	2003-2004	5.7y	3581	35	-0.15 [-0.46, 0.15]
EDEN	FR	2003-2006	5.7y	980	14	0.23 [-0.29, 0.75]
pooled mean difference (I^2 = 0.0%)						-0.11 [-0.16, -0.07]

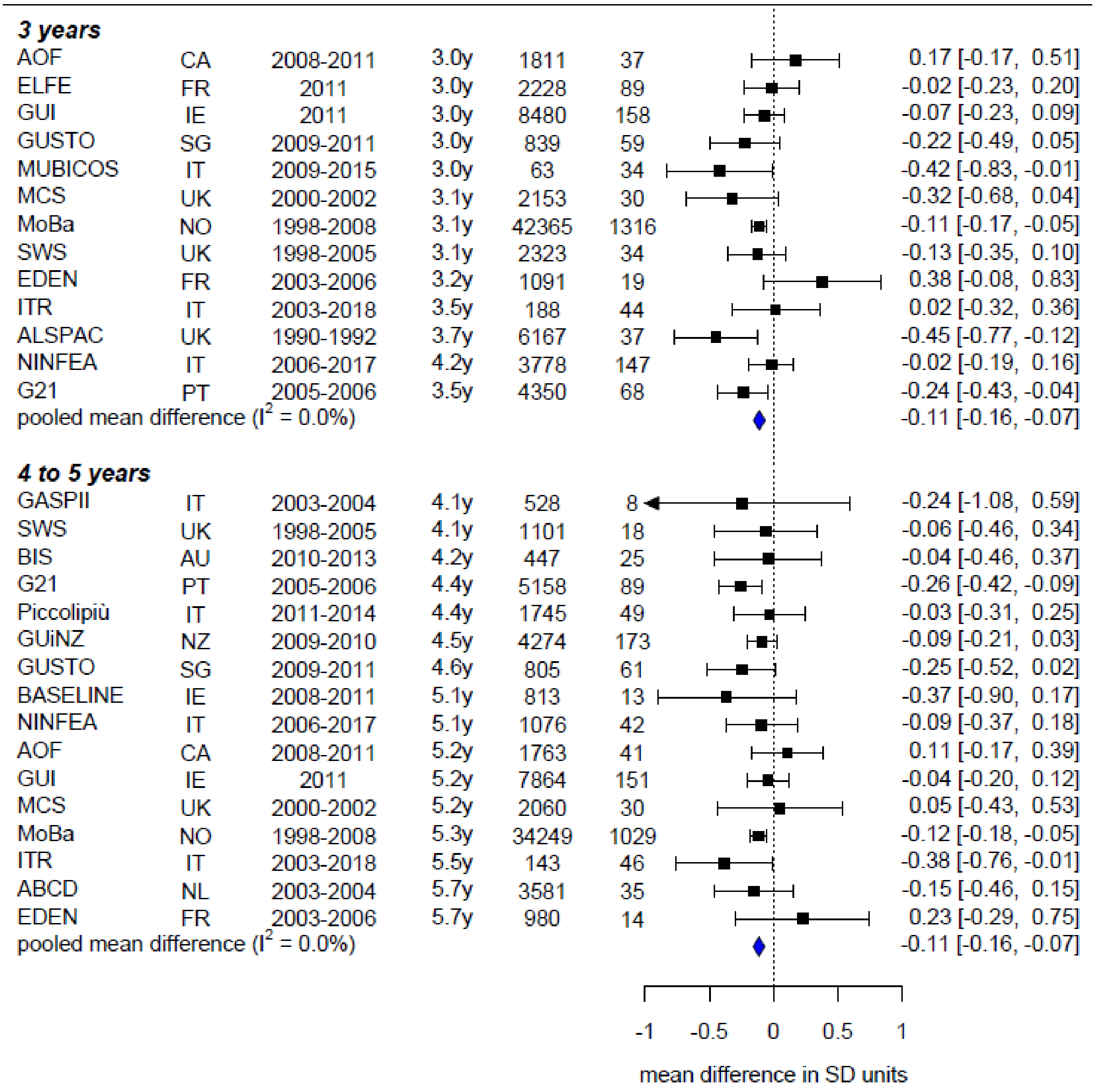

© 2022 Elhakeem A et al. *JAMA Network Open.*

d. weight: age groups 10 to 13

Cohort name	Cohort Country	Birth years	Mean age	NC	ART	Estimate [95% CI]
6 to 9 years						
SWS	UK	1998-2005	6.7y	1822	28	0.02 [-0.26, 0.30]
NINFEA	IT	2006-2017	7.3y	1940	77	-0.14 [-0.34, 0.07]
MoBa	NO	1998-2008	7.1y	44397	1264	-0.06 [-0.12, -0.00]
MCS	UK	2000-2002	6.8y	1925	27	-0.21 [-0.59, 0.17]
ITR	IT	2003-2018	8.0y	367	46	0.18 [-0.17, 0.53]
GUSTO	SG	2009-2011	6.1y	748	54	-0.34 [-0.62, -0.06]
GUiNZ	NZ	2009-2010	8.6y	3755	157	-0.02 [-0.12, 0.09]
Gen R	NL	2002-2006	6.2y	4334	45	-0.04 [-0.32, 0.24]
G21	PT	2005-2006	7.2y	5746	98	-0.24 [-0.41, -0.07]
GASPII	IT	2003-2004	7.8y	453	8	-0.22 [-1.08, 0.65]
DNBC	DK	1996-2000	7.0y	34899	1481	-0.06 [-0.11, -0.01]
ALSPAC	UK	1990-1992	7.5y	6167	47	-0.16 [-0.40, 0.09]
ABCD	NL	2003-2004	10.6y	2741	36	-0.15 [-0.46, 0.16]
pooled mean difference (I^2 = 0.0%)						-0.07 [-0.10, -0.04]
10 to 13 years						
SWS	UK	1998-2005	9.2y	1122	19	-0.16 [-0.52, 0.20]
NINFEA	IT	2006-2017	10.2y	863	34	0.05 [-0.26, 0.35]
MCS	UK	2000-2002	11.2y	1826	27	-0.17 [-0.62, 0.28]
ITR	IT	2003-2018	13.6y	819	34	0.21 [-0.15, 0.57]
HGS	GR	2007-2009	11.2y	2182	63	-0.30 [-0.54, -0.05]
Gen R	NL	2002-2006	9.8y	3754	42	-0.09 [-0.38, 0.20]
G21	PT	2005-2006	10.2y	4664	92	-0.29 [-0.48, -0.10]
DNBC	DK	1996-2000	11.3y	35821	1393	0.02 [-0.09, 0.02]
ALSPAC	UK	1990-1992	12.8y	5089	47	-0.00 [-0.24, 0.23]
ABCD	NL	2003-2004	11.7y	2304	30	-0.03 [-0.35, 0.29]
pooled mean difference (I^2 = 49.0%)						-0.08 [-0.18, 0.03]
14 to 17 years						
MCS	UK	2000-2002	17.2y	1391	20	-0.01 [-0.41, 0.40]
HUNT	NO	1984-2006	15.0y	9711	121	-0.02 [-0.19, 0.14]
ALSPAC	UK	1990-1992	15.5y	4109	37	-0.02 [-0.30, 0.27]
pooled mean difference (I^2 = 0.0%)						-0.02 [-0.15, 0.12]
>17 years						
HUNT	NO	1984-2006	18.1y	7281	42	0.15 [-0.38, 0.69]
CHART	AU	1982-1992	27.4y	73	130	0.02 [-0.32, 0.37]
ALSPAC	UK	1990-1992	24.5y	2976	31	0.07 [-0.22, 0.36]
pooled mean difference (I^2 = 0.0%)						0.07 [-0.14, 0.27]

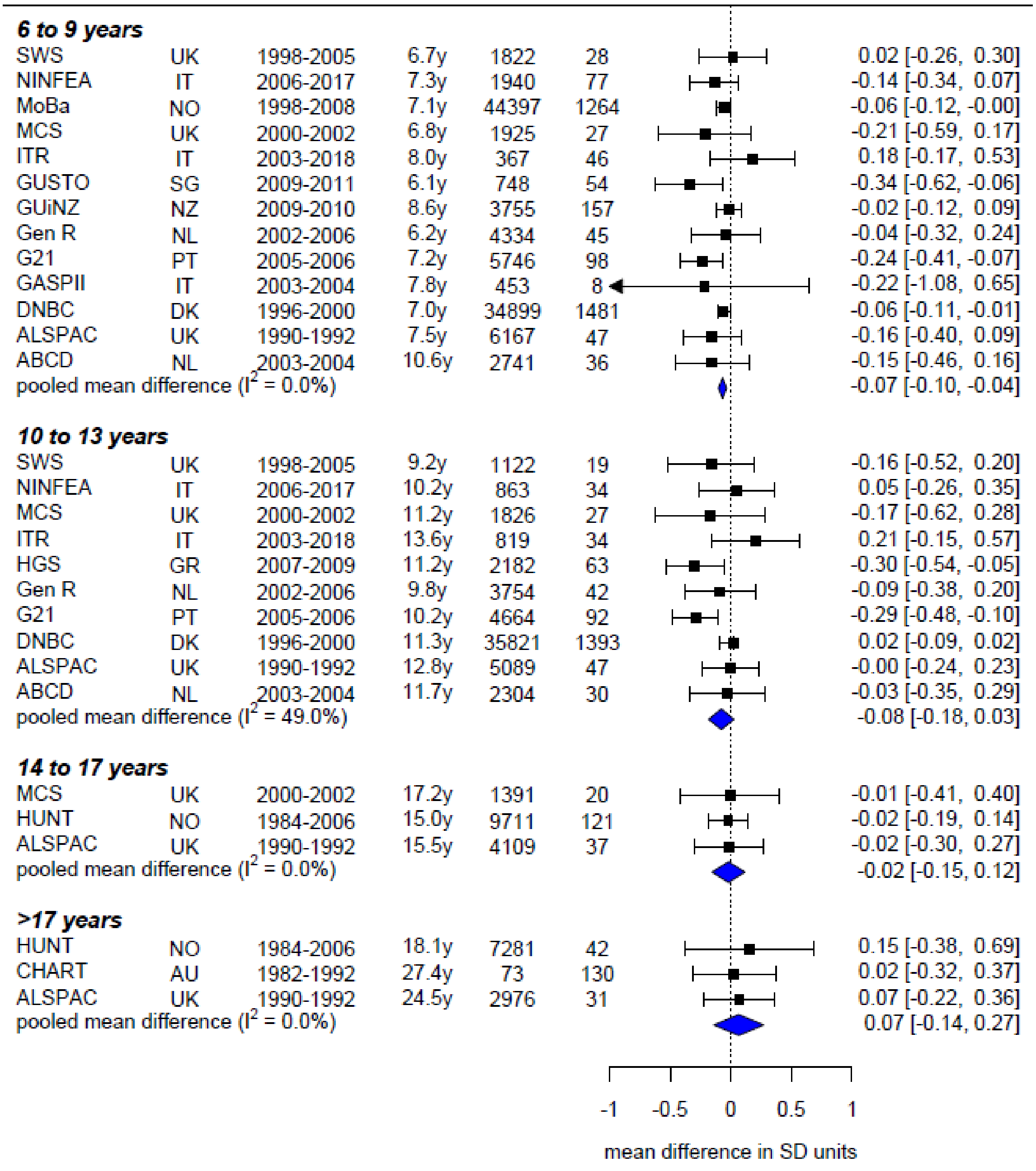

Estimates represent the cohort-specifc confounder-adjusted mean differences in SD units [and 95% confidence intervals] in weight at each age group between ART-conceived and NC offspring (ART minus NC). Estimates were adjusted (as fully as possible) for maternal age, parity, BMI, smoking, education, ethnicity (or country of birth), plus offspring sex and age at outcome assessment. Cohorts are arranged by the offspring's mean age at outcome assessment. Blue diamonds represent pooled mean differences from random-effects meta-analyses. NC is the number of NC ofspring; ART is the number of ART-conceived offspring; I² represents the percentage of total variability due to between cohort heterogeneity.

© 2022 Elhakeem A et al. *JAMA Network Open.*

eFigure 4. Cohort-Specific Mean Differences in Body Mass Index Between Offspring Conceived via ART and Those Who Were NC

a. body mass index: age groups 1 to 4

Cohort name	Cohort Country	Birth years	Mean age	NC	ART	Estimate [95% CI]
<3 months						
CHOP	EUR	2002-2004	0.6m	1472	20	-0.16 [-0.41, 0.10]
GUSTO	SG	2009-2011	0.7m	932	63	0.01 [-0.24, 0.27]
Piccolipiù	IT	2011-2014	1.0m	2213	80	-0.08 [-0.31, 0.15]
Gen R	NL	2002-2006	1.1m	3146	41	-0.04 [-0.34, 0.26]
BIGCS	CN	2012-2025	1.3m	9668	344	-0.09 [-0.20, 0.01]
MoBa	NO	1998-2008	1.5m	54263	1556	-0.40 [-0.46, -0.35]
ALSPAC	UK	1990-1992	1.7m	8106	49	-0.17 [-0.42, 0.07]
BASELINE	IE	2008-2011	2.2m	961	16	-0.41 [-0.91, 0.08]
ABCD	NL	2003-2004	2.5m	4288	60	-0.13 [-0.36, 0.10]
G21	PT	2005-2006	2.4m	4887	68	-0.25 [-0.44, -0.07]
pooled mean difference (I^2 = 66.9%)						-0.18 [-0.28, -0.08]
3 to 5 months						
CHOP	EUR	2002-2004	3.0m	1214	16	-0.05 [-0.46, 0.37]
NINFEA	IT	2006-2017	3.0m	4181	229	-0.10 [-0.25, 0.04]
Piccolipiù	IT	2011-2014	3.0m	2088	76	-0.17 [-0.40, 0.07]
GUSTO	SG	2009-2011	3.1m	918	66	-0.13 [-0.39, 0.13]
MoBa	NO	1998-2008	3.2m	75106	2083	-0.21 [-0.26, -0.16]
EDEN	FR	2003-2006	3.6m	1325	21	0.21 [-0.22, 0.64]
ABCD	NL	2003-2004	4.0m	4394	50	-0.26 [-0.51, -0.01]
ELFE	FR	2011	4.1m	9322	304	-0.01 [-0.12, 0.10]
DNBC	DK	1996-2000	4.8m	45203	1292	0.02 [-0.03, 0.08]
ALSPAC	UK	1990-1992	5.1m	1306	15	-0.28 [-0.71, 0.15]
G21	PT	2005-2006	4.8m	5163	70	-0.14 [-0.35, 0.07]
pooled mean difference (I^2 = 66.1%)						-0.10 [-0.18, -0.02]
6 to 8 months						
CHOP	EUR	2002-2004	6.0m	1107	15	0.00 [-0.39, 0.40]
Piccolipiù	IT	2011-2014	6.0m	1775	65	0.03 [-0.23, 0.28]
GUSTO	SG	2009-2011	6.1m	880	62	-0.11 [-0.38, 0.15]
BASELINE	IE	2008-2011	6.2m	1024	18	-0.35 [-0.82, 0.12]
BIGCS	CN	2012-2027	6.3m	9770	373	0.05 [-0.05, 0.16]
SWS	UK	1998-2005	6.3m	2132	24	-0.04 [-0.44, 0.36]
Gen R	NL	2002-2006	6.4m	3738	45	-0.04 [-0.32, 0.23]
ITR	IT	2003-2018	6.8m	156	48	-0.15 [-0.58, 0.27]
NINFEA	IT	2006-2017	7.0m	4485	253	-0.12 [-0.25, 0.01]
ABCD	NL	2003-2004	7.5m	4273	49	-0.18 [-0.44, 0.08]
MoBa	NO	1998-2008	8.3m	57342	1689	-0.06 [-0.12, -0.01]
EDEN	FR	2003-2006	8.8m	1141	17	0.43 [-0.06, 0.91]
G21	PT	2005-2006	7.2m	4983	68	-0.13 [-0.39, 0.13]
pooled mean difference (I^2 = 12.7%)						-0.05 [-0.11, 0.00]
9 to 11 months						
GUI	IE	2011	9.0m	9735	173	-0.06 [-0.18, 0.06]
ELFE	FR	2011	9.1m	8940	295	-0.03 [-0.14, 0.09]
GUSTO	SG	2009-2011	9.1m	841	64	-0.04 [-0.31, 0.23]
ALSPAC	UK	1990-1992	9.4m	7696	46	-0.29 [-0.56, -0.02]
ABCD	NL	2003-2004	9.8m	4225	46	-0.13 [-0.39, 0.13]
Gen R	NL	2002-2006	11.2m	3864	45	0.04 [-0.24, 0.32]
BIGCS	CN	2012-2025	12.0m	6848	271	0.13 [-0.00, 0.25]
MoBa	NO	1998-2008	12.3m	60074	1777	-0.07 [-0.12, -0.02]
G21	PT	2005-2006	9.6m	4264	57	-0.13 [-0.41, 0.15]
pooled mean difference (I^2 = 39.2%)						-0.04 [-0.11, 0.02]

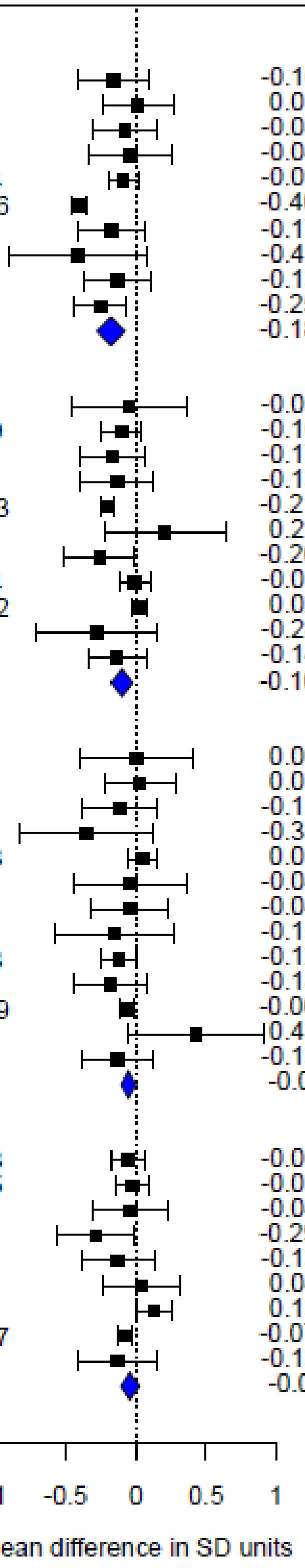

© 2022 Elhakeem A et al. *JAMA Network Open.*

b. body mass index: age groups 5 to 7

Cohort name	Cohort Country	Birth years	Mean age	NC	ART	Estimate [95% CI]
12 to 16 months						
CHOP	EUR	2002-2004	12.0m	1003	15	-0.05 [-0.49, 0.39]
DNBC	DK	1996-2000	12.0m	42116	1211	0.03 [-0.03, 0.09]
NINFEA	IT	2006-2017	12.0m	3929	190	-0.05 [-0.21, 0.10]
Piccolipiù	IT	2011-2014	12.0m	1520	55	-0.17 [-0.44, 0.11]
GUSTO	SG	2009-2011	12.1m	860	62	0.11 [-0.16, 0.38]
EDEN	FR	2003-2006	12.2m	1361	21	0.24 [-0.19, 0.68]
ELFE	FR	2011	12.2m	7866	266	-0.09 [-0.21, 0.03]
BASELINE	IE	2008-2011	12.6m	958	19	-0.27 [-0.73, 0.19]
SWS	UK	1998-2005	12.6m	2465	35	0.07 [-0.21, 0.36]
BIS	AU	2010-2013	13.0m	627	35	-0.12 [-0.46, 0.22]
ABCD	NL	2003-2004	13.1m	4075	49	-0.16 [-0.42, 0.10]
AOF	CA	2008-2011	13.2m	1292	35	0.07 [-0.45, 0.59]
MUBICOS	IT	2009-2015	13.2m	98	54	-0.28 [-0.74, 0.17]
MoBa	NO	1998-2008	16.0m	47486	1398	-0.09 [-0.14, -0.03]
GASPII	IT	2003-2004	16.2m	543	7	-0.07 [-0.80, 0.65]
G21	PT	2005-2006	14.4m	4996	72	-0.22 [-0.43, -0.00]
MoBa	NO	1998-2008	17.3m	14190	471	-0.06 [-0.16, 0.04]
pooled mean difference (I^2 = 28.5%)						-0.06 [-0.11, -0.00]
17 to 23 months						
Piccolipiù	IT	2011-2014	18.0m	1607	59	-0.14 [-0.41, 0.12]
ABCD	NL	2003-2004	18.1m	3912	48	-0.10 [-0.38, 0.17]
GUSTO	SG	2009-2011	18.4m	772	50	0.02 [-0.27, 0.32]
NINFEA	IT	2006-2017	19.3m	4393	221	-0.11 [-0.25, 0.03]
ALSPAC	UK	1990-1992	20.4m	7060	44	-0.15 [-0.63, 0.33]
G21	PT	2005-2006	20.4m	4904	68	-0.13 [-0.36, 0.10]
ITR	IT	2003-2018	17.4m	232	53	0.04 [-0.31, 0.39]
pooled mean difference (I^2 = 0.0%)						-0.08 [-0.15, -0.01]
2 years						
SWS	UK	1998-2005	2.0y	2331	34	0.03 [-0.31, 0.37]
AOF	CA	2008-2011	2.0y	1357	41	-0.17 [-0.33, -0.01]
EDEN	FR	2003-2006	2.0y	1154	17	0.11 [-0.36, 0.59]
ELFE	FR	2011	2.0y	6745	227	-0.06 [-0.19, 0.06]
GUiNZ	NZ	2009-2010	2.0y	3721	152	0.07 [-0.08, 0.22]
GUSTO	SG	2009-2011	2.0y	800	56	-0.13 [-0.41, 0.16]
Piccolipiù	IT	2011-2014	2.0y	1717	71	-0.03 [-0.28, 0.21]
ABCD	NL	2003-2004	2.1y	3798	44	0.01 [-0.28, 0.30]
Gen R	NL	2002-2006	2.1y	3432	40	0.11 [-0.20, 0.42]
BASELINE	IE	2008-2011	2.2y	1004	20	-0.21 [-0.65, 0.24]
MoBa	NO	1998-2008	2.2y	28809	895	-0.13 [-0.19, -0.06]
ITR	IT	2003-2018	2.5y	190	54	0.07 [-0.33, 0.47]
BIGCS	CN	2012-2027	2.8y	6055	268	-0.06 [-0.19, 0.06]
G21	PT	2005-2006	2.6y	4769	69	-0.23 [-0.43, -0.04]
pooled mean difference (I^2 = 16.1%)						-0.08 [-0.13, -0.02]

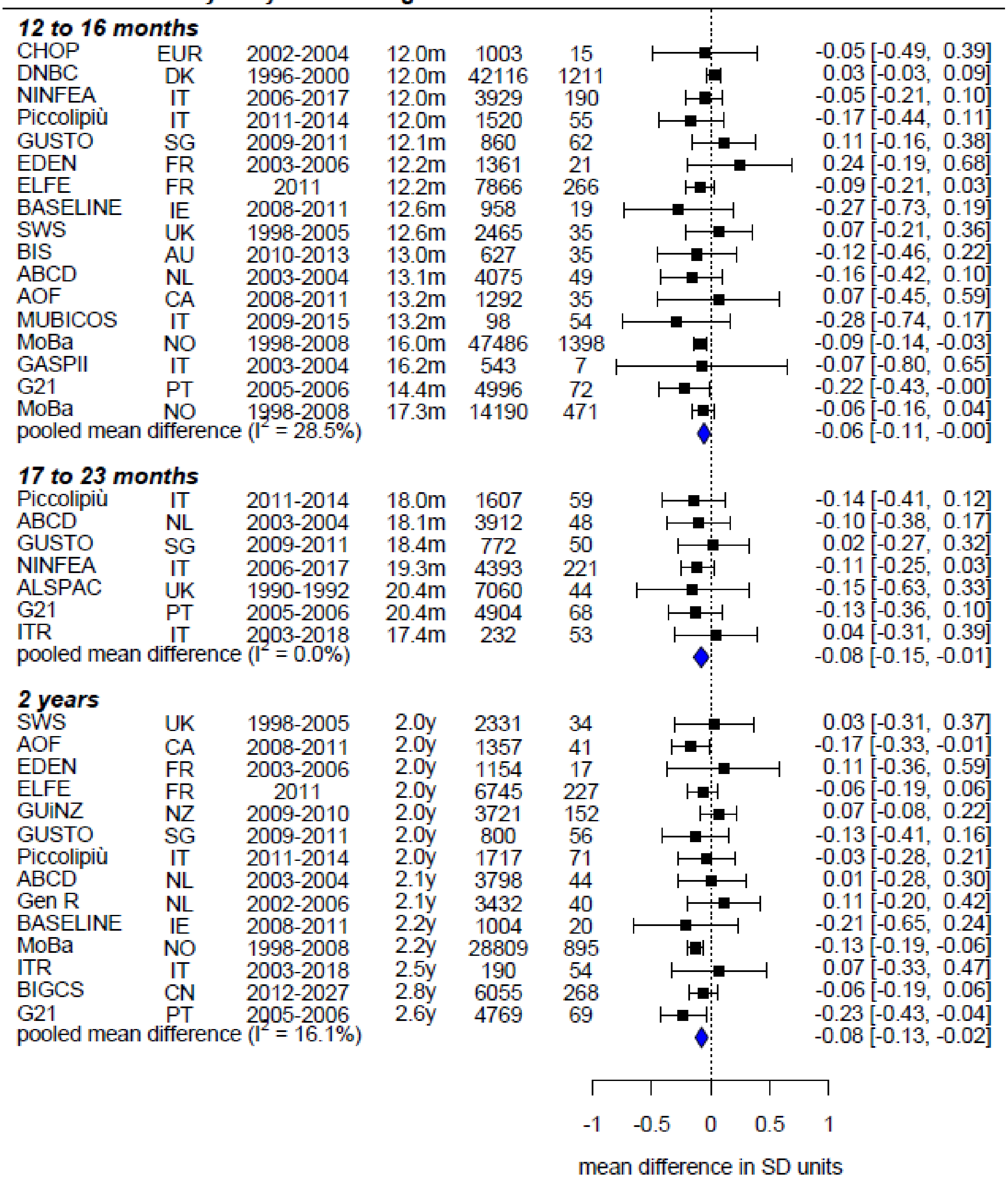

© 2022 Elhakeem A et al. *JAMA Network Open.*

c. body mass index: age groups 8 and 9

Cohort name	Cohort Country	Birth years	Mean age	NC	ART	Estimate [95% CI]
3 years						
AOF	CA	2008-2011	3.0y	1774	37	0.16 [-0.14, 0.46]
ELFE	FR	2011	3.0y	2217	89	-0.05 [-0.27, 0.17]
GUI	IE	2011	3.0y	9437	157	-0.09 [-0.25, 0.06]
GUSTO	SG	2009-2011	3.0y	832	59	-0.03 [-0.29, 0.24]
MUBICOS	IT	2009-2015	3.0y	61	34	-0.39 [-0.82, 0.05]
MCS	UK	2000-2002	3.1y	2107	30	-0.19 [-0.52, 0.15]
MoBa	NO	1998-2008	3.1y	39431	1233	-0.11 [-0.17, -0.05]
SWS	UK	1998-2005	3.1y	2280	33	0.11 [-0.12, 0.34]
EDEN	FR	2003-2006	3.2y	1088	19	0.03 [-0.42, 0.49]
ITR	IT	2003-2018	3.5y	186	42	-0.12 [-0.54, 0.29]
ALSPAC	UK	1990-1992	3.7y	6492	37	-0.49 [-0.82, -0.15]
NINFEA	IT	2006-2017	4.2y	3673	143	-0.02 [-0.19, 0.15]
G21	PT	2005-2006	3.5y	4350	68	-0.15 [-0.35, 0.05]
pooled mean difference (I^2 = 0.1%)						-0.10 [-0.14, -0.05]
4 to 5 years						
GASPII	IT	2003-2004	4.1y	528	8	-0.22 [-1.00, 0.57]
SWS	UK	1998-2005	4.1y	1097	18	-0.03 [-0.37, 0.31]
BIS	AU	2010-2013	4.2y	535	26	0.04 [-0.34, 0.41]
G21	PT	2005-2006	4.4y	5150	89	-0.20 [-0.38, -0.01]
Piccolipiù	IT	2011-2014	4.4y	1758	49	-0.08 [-0.37, 0.20]
GUiNZ	NZ	2009-2010	4.5y	4274	173	-0.03 [-0.15, 0.10]
GUSTO	SG	2009-2011	4.6y	805	61	-0.11 [-0.38, 0.15]
BASELINE	IE	2008-2011	5.1y	812	13	-0.49 [-1.03, 0.05]
NINFEA	IT	2006-2017	5.1y	971	39	0.07 [-0.32, 0.46]
AOF	CA	2008-2011	5.2y	1725	41	-0.05 [-0.23, 0.12]
GUI	IE	2011	5.2y	7850	151	-0.08 [-0.23, 0.08]
MCS	UK	2000-2002	5.2y	2058	30	-0.07 [-0.53, 0.38]
MoBa	NO	1998-2008	5.3y	33142	1006	-0.12 [-0.18, -0.06]
ITR	IT	2003-2018	5.5y	140	46	-0.54 [-0.97, -0.10]
ABCD	NL	2003-2004	5.7y	3498	35	-0.06 [-0.36, 0.24]
EDEN	FR	2003-2006	5.7y	981	14	-0.10 [-0.61, 0.42]
pooled mean difference (I^2 = 0.0%)						-0.10 [-0.15, -0.06]

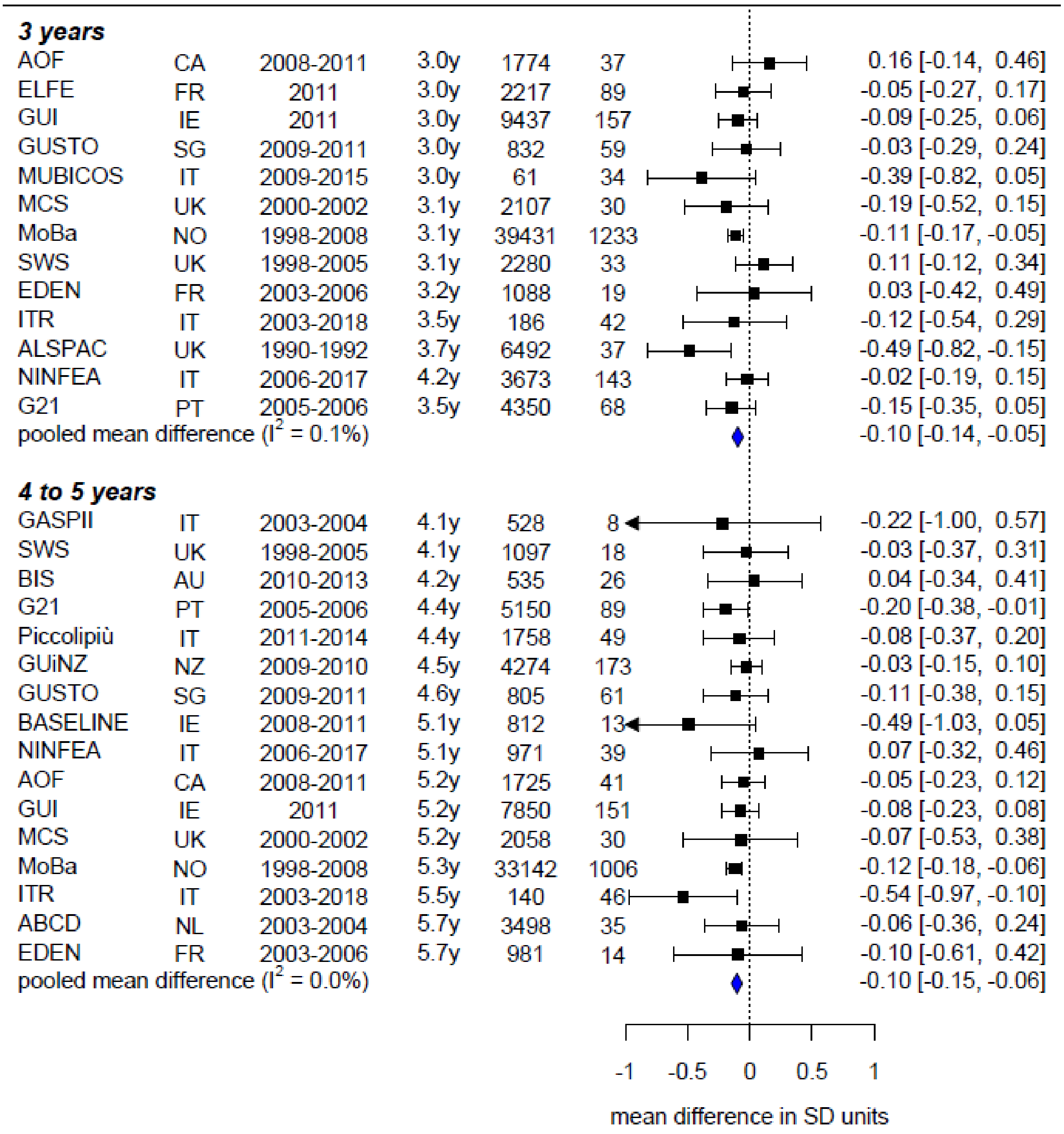

© 2022 Elhakeem A et al. *JAMA Network Open.*

d. body mass index: age groups 10 to 13

Cohort name	Cohort Country	Birth years	Mean age	NC	ART	Estimate [95% CI]
6 to 9 years						
SWS	UK	1998-2005	6.7y	1818	28	0.14 [-0.11, 0.39]
NINFEA	IT	2006-2017	7.3y	1876	74	-0.07 [-0.28, 0.15]
MoBa	NO	1998-2008	7.1y	43087	1236	-0.09 [-0.14, -0.03]
MCS	UK	2000-2002	6.8y	1925	27	-0.22 [-0.62, 0.19]
ITR	IT	2003-2018	8.0y	365	46	-0.05 [-0.41, 0.30]
GUSTO	SG	2009-2011	6.1y	747	54	-0.25 [-0.52, 0.03]
GUiNZ	NZ	2009-2010	8.6y	3751	157	0.02 [-0.11, 0.14]
Gen R	NL	2002-2006	6.2y	4334	45	0.07 [-0.17, 0.32]
G21	PT	2005-2006	7.2y	5591	98	-0.23 [-0.40, -0.05]
GASPII	IT	2003-2004	7.8y	453	8	-0.37 [-1.19, 0.46]
DNBC	DK	1996-2000	7.0y	34899	1481	-0.00 [-0.05, 0.05]
ALSPAC	UK	1990-1992	7.5y	6166	47	-0.17 [-0.42, 0.07]
ABCD	NL	2003-2004	10.6y	2738	36	-0.22 [-0.52, 0.08]
pooled mean difference (I^2 = 36.7%)						-0.06 [-0.12, -0.01]
10 to 13 years						
SWS	UK	1998-2005	9.2y	1121	19	-0.12 [-0.47, 0.22]
NINFEA	IT	2006-2017	10.2y	846	33	0.12 [-0.23, 0.47]
MCS	UK	2000-2002	11.2y	1826	27	-0.14 [-0.61, 0.33]
ITR	IT	2003-2018	13.6y	815	32	0.11 [-0.29, 0.52]
HGS	GR	2007-2009	11.2y	2182	63	-0.24 [-0.49, 0.00]
Gen R	NL	2002-2006	9.8y	3754	42	0.01 [-0.27, 0.29]
G21	PT	2005-2006	10.2y	4663	92	-0.28 [-0.47, -0.09]
DNBC	DK	1996-2000	11.3y	35821	1393	0.01 [-0.04, 0.07]
ALSPAC	UK	1990-1992	12.8y	5089	47	0.00 [-0.24, 0.25]
ABCD	NL	2003-2004	11.7y	2236	28	-0.08 [-0.39, 0.23]
pooled mean difference (I^2 = 38.0%)						-0.07 [-0.16, 0.03]
14 to 17 years						
MCS	UK	2000-2002	17.2y	1390	20	-0.03 [-0.44, 0.38]
HUNT	NO	1984-2006	15.0y	9704	121	0.01 [-0.17, 0.18]
ALSPAC	UK	1990-1992	15.5y	4109	37	0.04 [-0.27, 0.34]
pooled mean difference (I^2 = 0.0%)						0.01 [-0.13, 0.15]
>17 years						
HUNT	NO	1984-2006	18.1y	7278	42	0.10 [-0.39, 0.59]
CHART	AU	1982-1992	27.4y	68	120	0.00 [-0.36, 0.36]
ALSPAC	UK	1990-1992	24.5y	2974	31	0.16 [-0.19, 0.51]
pooled mean difference (I^2 = 0.0%)						0.09 [-0.14, 0.31]

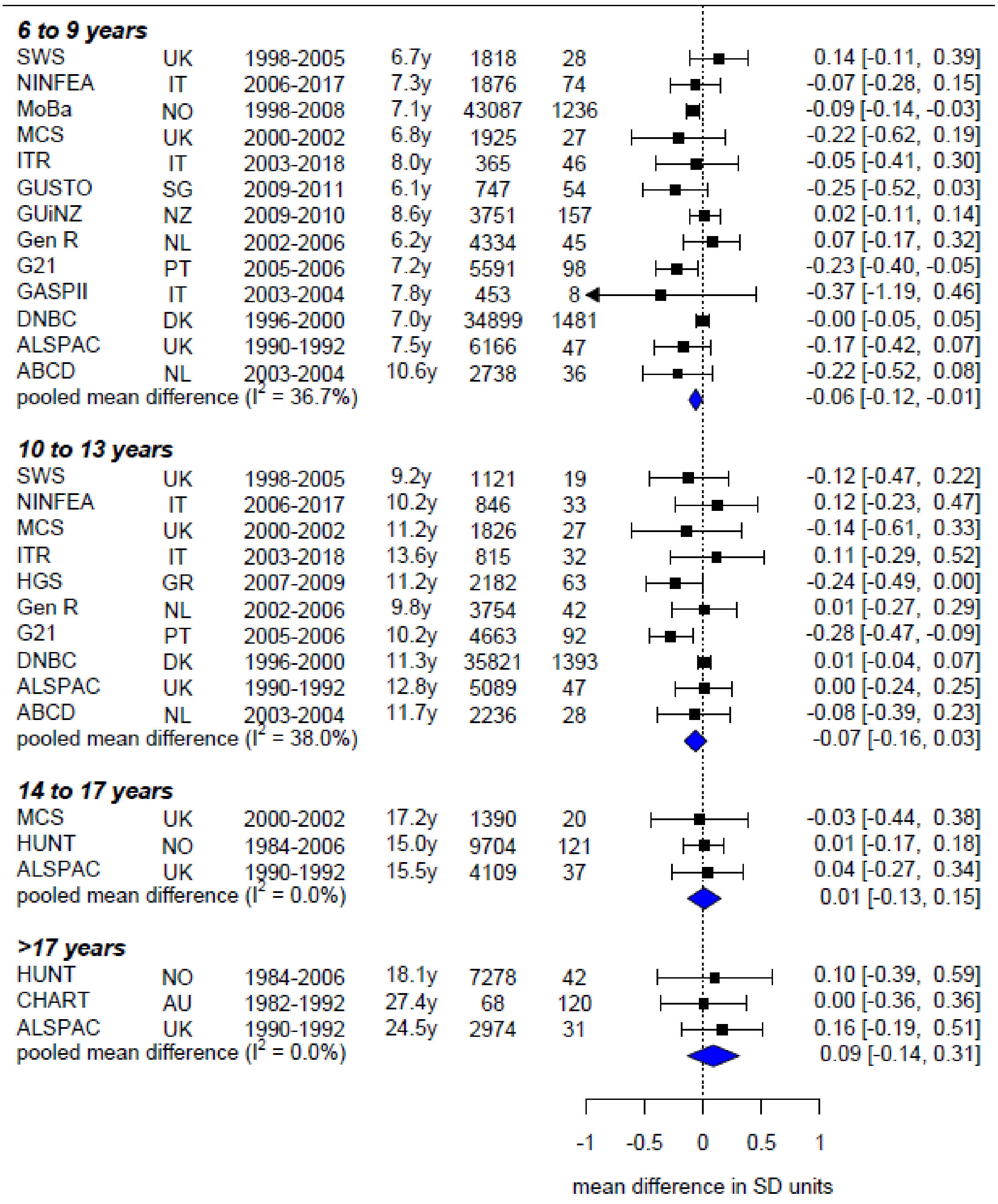

Estimates represent the cohort-specifc confounder-adjusted mean differences in SD units [and 95% confidence intervals] in body mass index at each age group between ART-conceived and NC offspring (ART minus NC). Estimates were adjusted (as fully as possible) for maternal age, parity, BMI, smoking, education, ethnicity (or country of birth), plus offspring sex and age at outcome assessment. Cohorts are arranged by the offspring's mean age at outcome assessment. Blue diamonds represent the pooled mean differences from random-effects meta-analyses. NC is the number of NC ofspring; ART is the number of ART-conceived offspring; I² represents the percentage of total variability due to between cohort heterogeneity.

© 2022 Elhakeem A et al. *JAMA Network Open.*

eFigure 5. Cohort-Specific Mean Differences in Waist Circumference Between Offspring Conceived via ART and Those Who Were NC

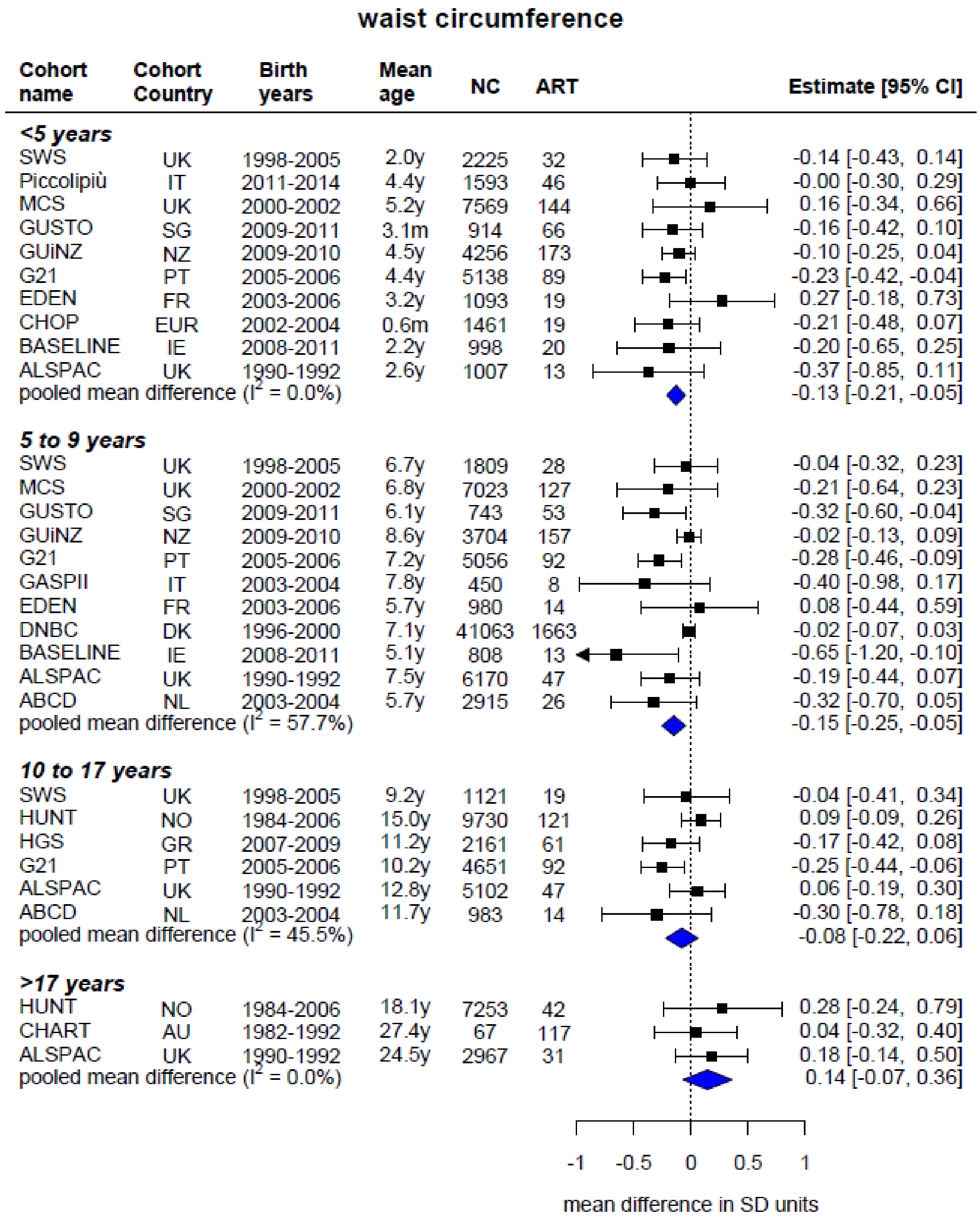

Estimates represent the cohort-specifc confounder-adjusted mean differences in SD units [and 95% confidence intervals] in waist circumference at each age group between ART-conceived and NC offspring (ART minus NC). Estimates were adjusted (as fully as possible) for maternal age, parity, BMI, smoking, education, ethnicity (or country of birth), plus offspring sex and age at outcome assessment. Cohorts are arranged by the offspring's mean age at outcome assessment. Blue diamonds represent the pooled mean differences from random-effects meta-analyses. NC is the number of NC ofspring; ART is the number of ART-conceived offspring; I² represents the percentage of total variability due to between cohort heterogeneity.

© 2022 Elhakeem A et al. *JAMA Network Open.*

eFigure 6. Cohort-Specific Mean Differences in Body Fat Percentage Between Offspring Conceived via ART and Those Who Were NC

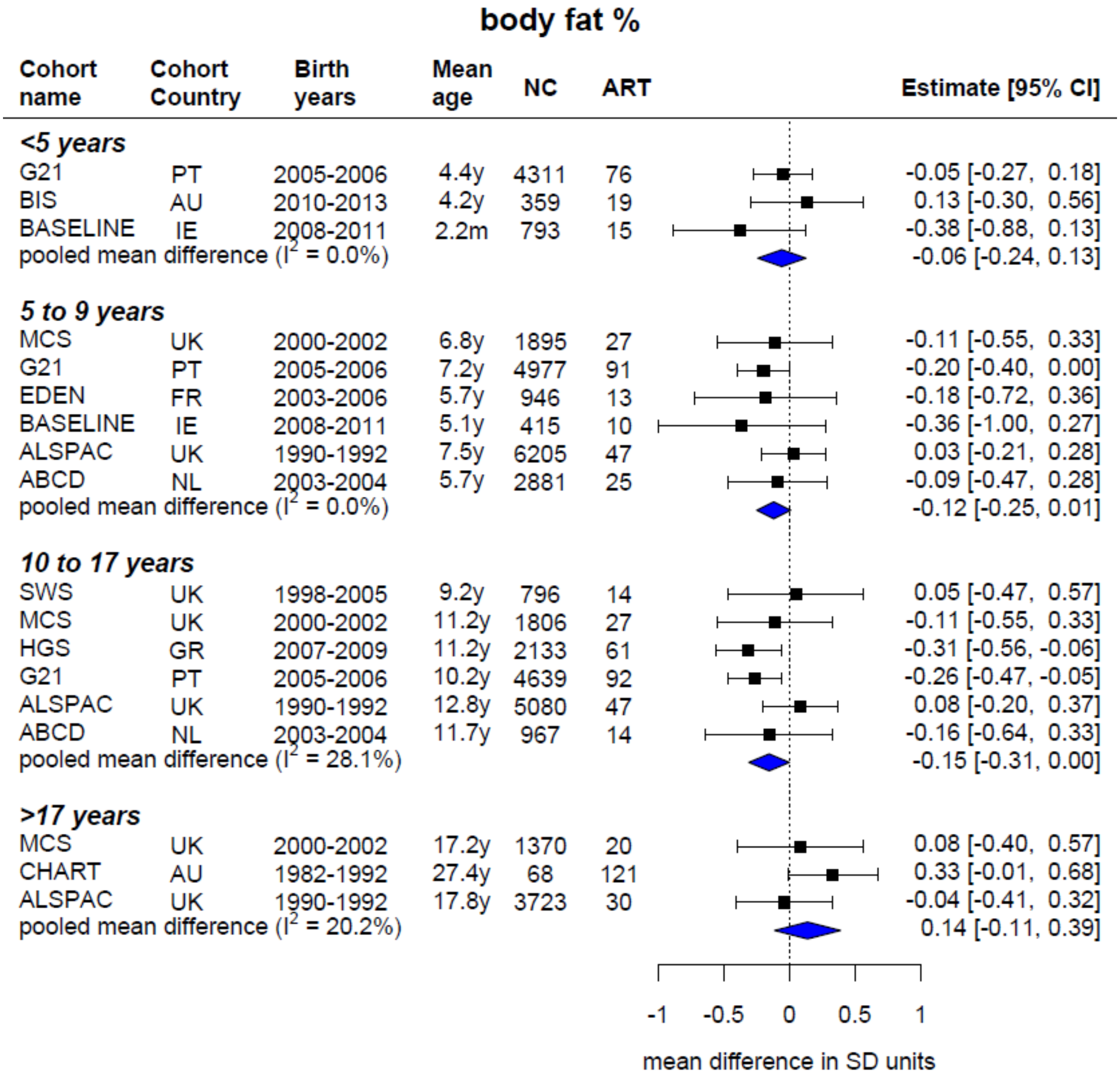

Estimates represent the cohort-specifc confounder-adjusted mean differences in SD units [and 95% confidence intervals] in body fat % at each age group between ART-conceived and NC offspring (ART minus NC). Estimates were adjusted (as fully as possible) for maternal age, parity, BMI, smoking, education, ethnicity (or country of birth), plus offspring sex and age at outcome assessment. Cohorts are arranged by the offspring's mean age at outcome assessment. Blue diamonds represent the pooled mean differences from random-effects meta-analyses. NC is the number of NC ofspring; ART is the number of ART-conceived offspring; I² represents the percentage of total variability due to between cohort heterogeneity.

eFigure 7. Cohort-Specific Mean Differences in Fat Mass Index Between Offspring Conceived via ART and Those Who Were NC

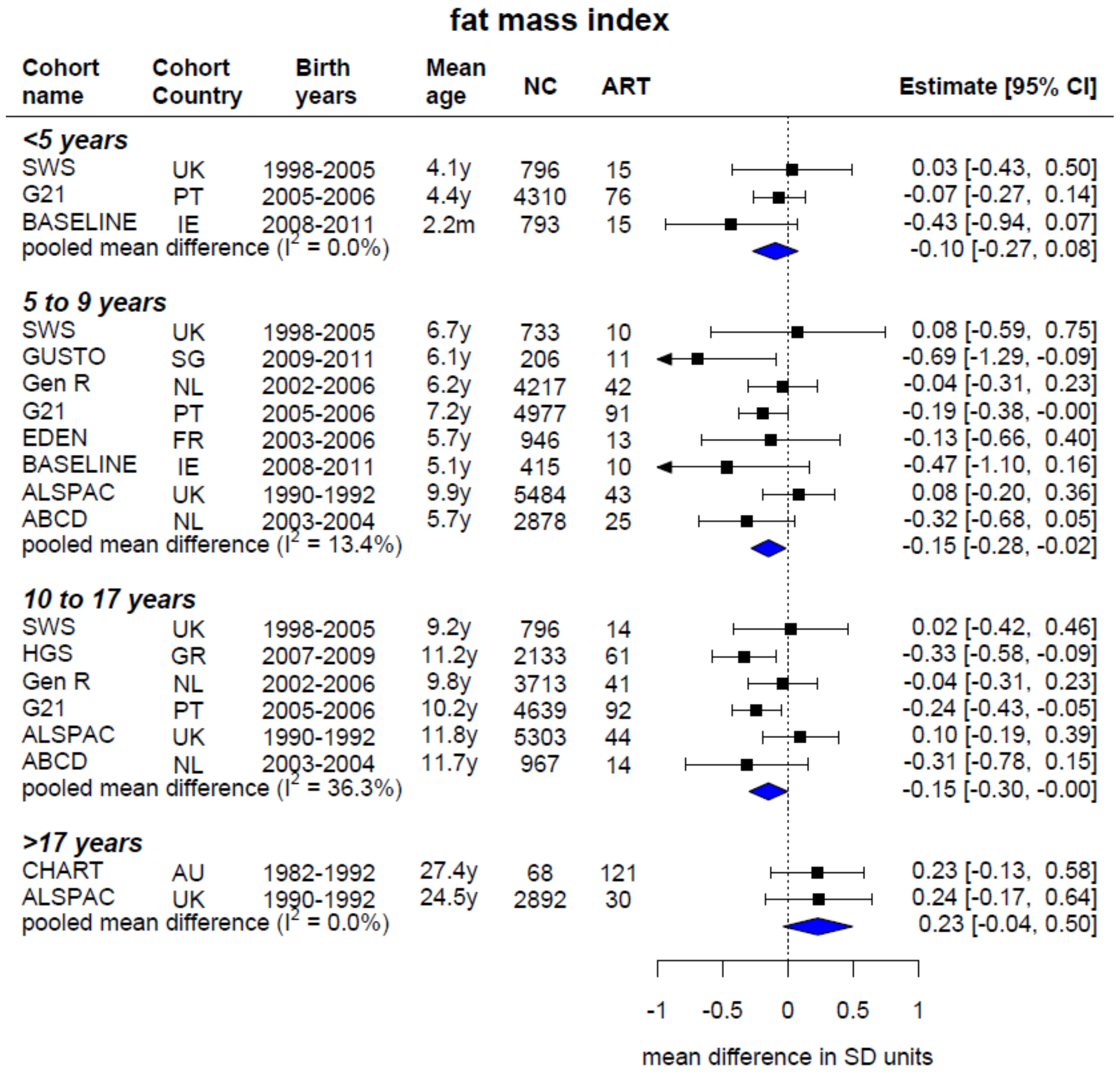

Estimates represent the cohort-specifc confounder-adjusted mean differences in SD units [and 95% confidence intervals] in fat mass index at each age group between ART-conceived and NC offspring (ART minus NC). Estimates were adjusted (as fully as possible) for maternal age, parity, BMI, smoking, education, ethnicity (or country of birth), plus offspring sex and age at outcome assessment. Cohorts are arranged by the offspring's mean age at outcome assessment. Blue diamonds represent the pooled mean differences from random-effects meta-analyses. NC is the number of NC ofspring; ART is the number of ART-conceived offspring; I² represents the percentage of total variability due to between cohort heterogeneity.

© 2022 Elhakeem A et al. *JAMA Network Open.*

eFigure 8. Mean Difference In Length / Height, Weight, and Body Mass Index at Ages Younger Than 3 Months and 3 to 5 Months Between Offspring Conceived via ART and Those Who Were NC, After Leaving Each Cohort Study Out of Meta-Analysis

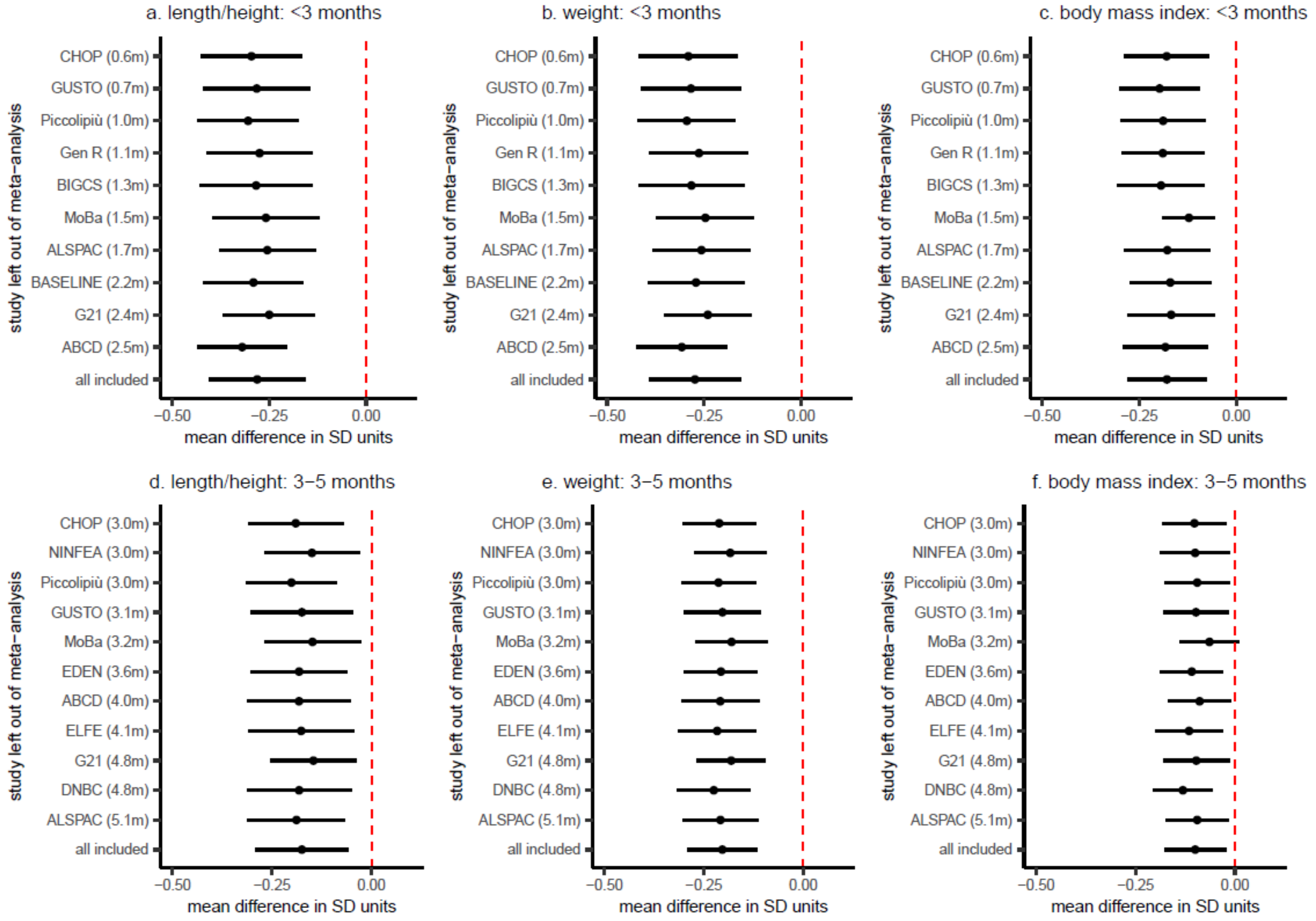

Estimates represent the confounder-adjusted pooled mean differences in SD units [and 95% confidence intervals] in length/height, weight, and body mass index at ages <3 months and 3-5 months between ART-conceived and NC offspring (ART minus NC), across all studies (bottom rows) and after refitting the meta-analysis models with each cohort study omitted in turn. Estimates were adjusted (as fully as possible) for maternal age, parity, BMI, smoking, education, ethnicity (or country of birth), plus offspring sex and age at outcome assessment. Cohorts arranged by offspring's mean age at outcome assessment.

© 2022 Elhakeem A et al. *JAMA Network Open.*

eFigure 9. Mean Difference in Growth and Adiposity Outcomes Between Offspring Conceived via ART and Those Who Were NC, Stratified by Sex

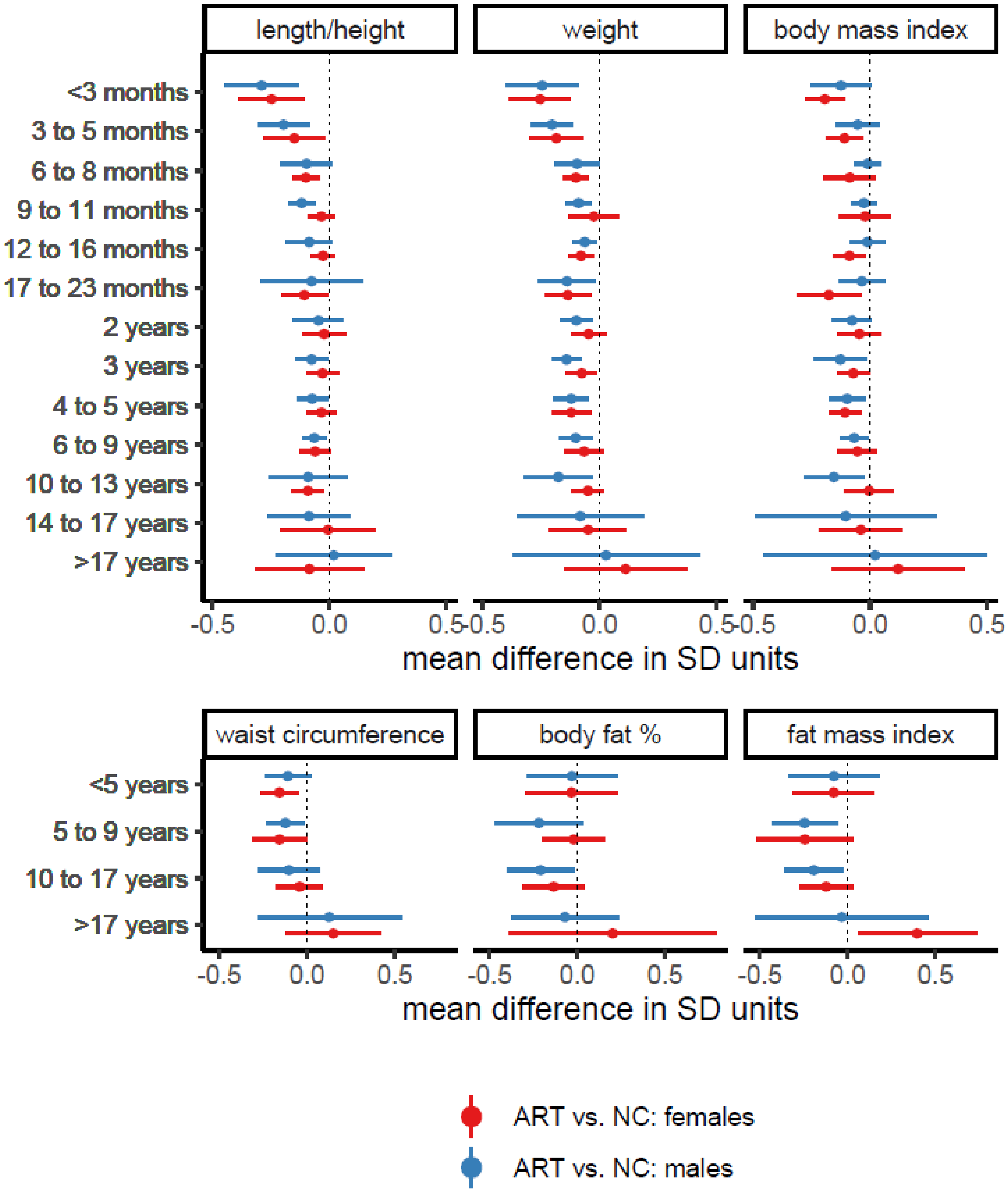

Estimates represent the confounder-adjusted pooled mean differences in SD units [and 95% confidence intervals] in growth and adiposity outcomes at each age group between ART-conceived and NC offspring (ART minus NC), seperately in females and males. Estimates were adjusted (as fully as possible) for maternal age, parity, BMI, smoking, education, ethnicity (or country of birth), plus offspring age at outcome assessment.

© 2022 Elhakeem A et al. *JAMA Network Open.*

eFigure 10. Mean Difference in Growth and Adiposity Outcomes Between Offspring Conceived via ART and Those Who Were NC, Comparing Results in all Participants With Singleton Births Only

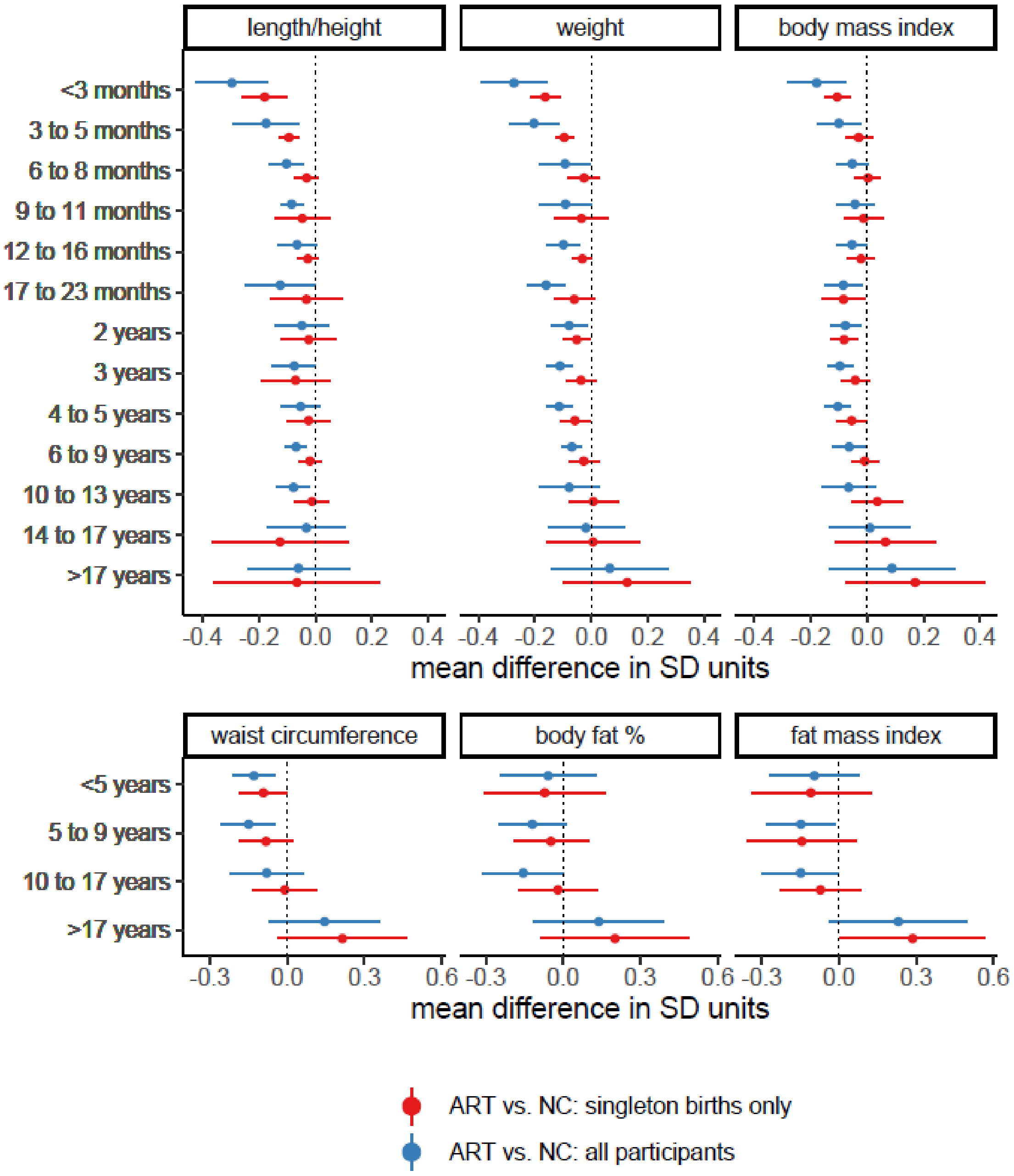

Estimates represent the confounder-adjusted pooled mean differences in SD units and 95% confidence intervals in growth and adiposity outcomes at each age group between ART-conceived and NC offspring (ART minus NC), comparing results in all participants (i.e., those presented in Figures 1-2) to singleton birth offspring. Cohort-specific estimates were adjusted (as fully as possible) for maternal age, parity, BMI, smoking, education, ethnicity (or country of birth), plus offspring sex and age at outcome assessment. Of the total 26 cohorts in this study, 15 cohorts included both singletons and multiple births, 9 cohorts included singletons only, and 2 cohorts included multiple births only.

© 2022 Elhakeem A et al. *JAMA Network Open.*

eFigure 11. Mean Difference in Growth and Adiposity Outcomes Between Offspring Conceived via ART and Those Who Were NC, After Further Adjustment for Birth Weight and Gestational Age

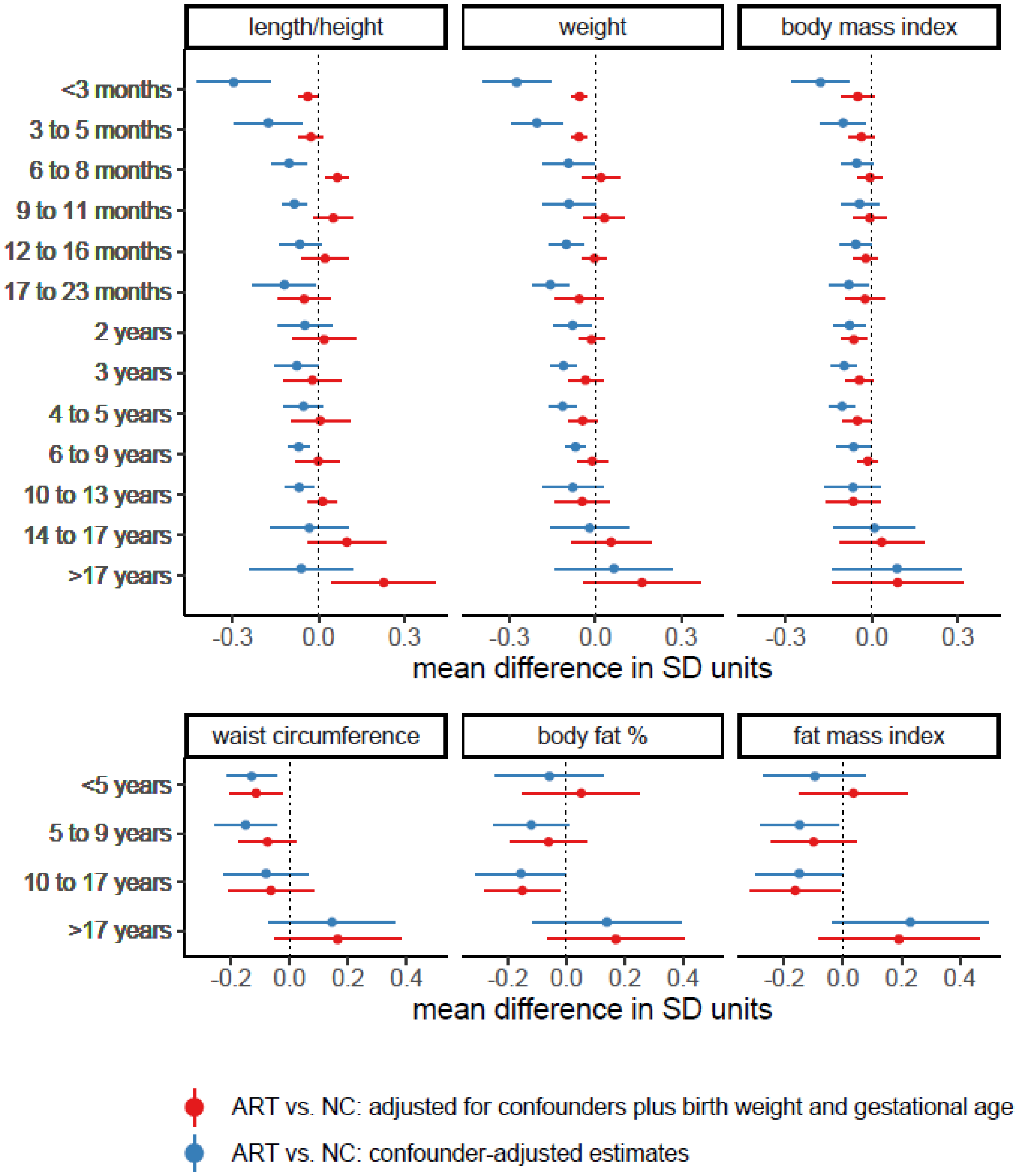

Estimates represent the confounder-adjusted pooled mean differences in SD units and 95% confidence intervals in growth and adiposity outcomes at each age group between ART-conceived and NC offspring (ART minus NC), before and after further adjustment for the potential mediators birthweight and gestational age. The confounder-adjusted estimates were adjusted (as fully as possible) for maternal age, parity, BMI, smoking, education, ethnicity (or country of birth), plus offspring sex and age at outcome assessment.

© 2022 Elhakeem A et al. *JAMA Network Open.*

eFigure 12. Mean Difference in Length / Height, Weight, and Body Mass Index Between Offspring Conceived via ART and Those Who Were NC, Separately for Fresh and Frozen-Thawed Embryo Transfer, After Further Adjustment for Birth Weight and Gestational Age

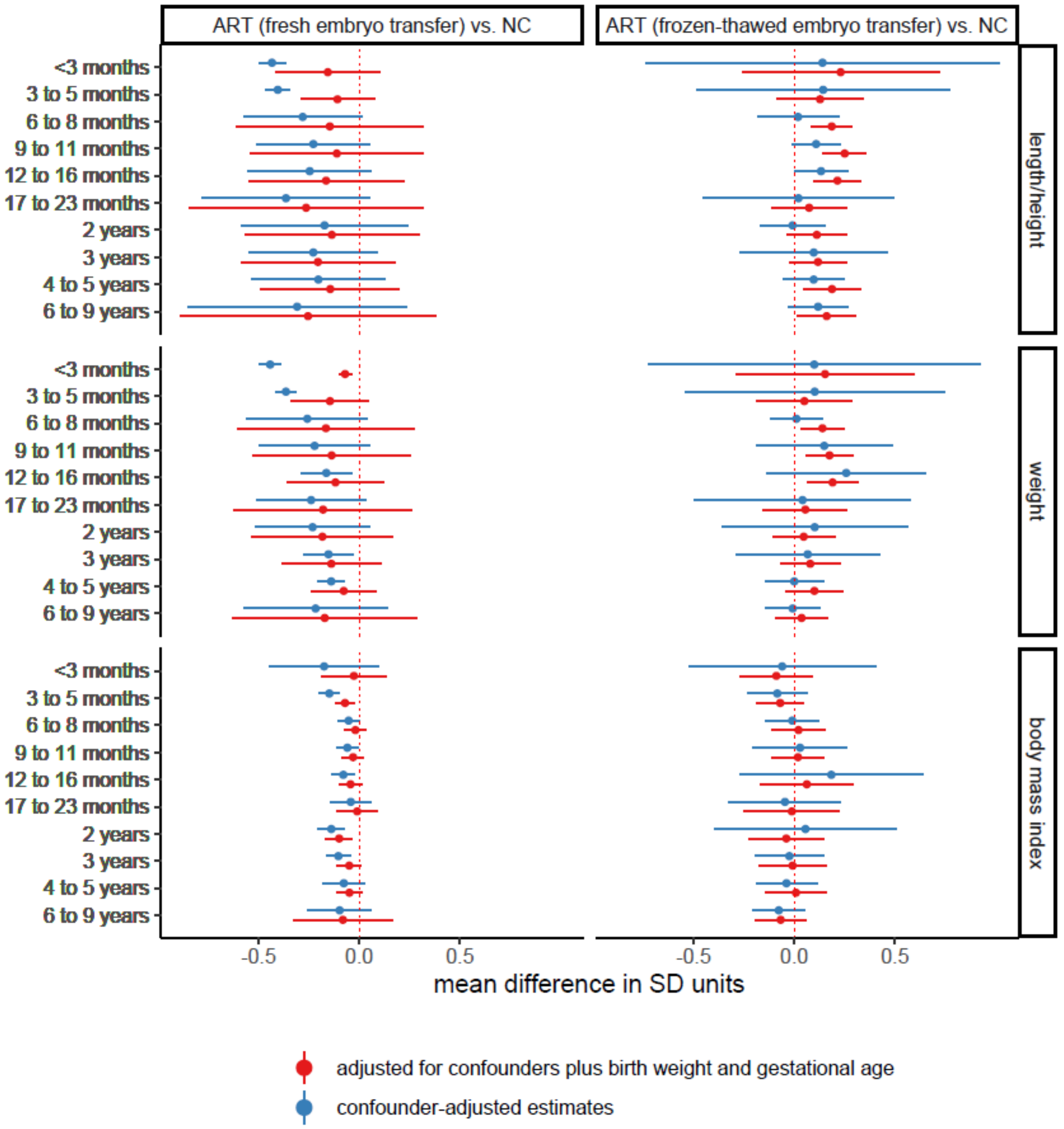

Estimates represent the confounder-adjusted pooled mean differences in SD units and 95% confidence intervals in length/height, weight, and body mass index at each age group between ART-conceived and NC offspring (ART minus NC), separately for fresh embryo transfer and frozen-thawed embryo transfer, before and after further adjustment for the potential mediators birthweight and gestational age. The confounder-adjusted estimates were adjusted (as fully as possible) for maternal age, parity, BMI, smoking, education, ethnicity (or country of birth), plus offspring sex and age at outcome assessment.

© 2022 Elhakeem A et al. *JAMA Network Open.*

eAppendix. Cohort-Specific Acknowledgements and Funding

All Our Families Study (AOF)

The authors acknowledge the tremendous contribution and support of AOB/F participants and AOB/F team members. All Our Families is funded through Alberta Innovates Interdisciplinary Team Grant #200700595, the Alberta Children's Hospital Foundation, the Canadian Institutes of Health Research, and the Social Sciences and Humanities Research Council.

The authors acknowledge the tremendous contribution and support of AOF participants and AOF team members. All Our Families is funded through Alberta Innovates Interdisciplinary Team Grant #200700595, the Alberta Children's Hospital Foundation, the Canadian Institutes of Health Research, and the Social Sciences and Humanities Research Council.

Avon Longitudinal Study of Parents and Children (ALSPAC)

We are extremely grateful to all of the families who took part in ALSPAC, the midwives for their help in recruiting them, and the whole ALSPAC team, which includes interviewers, computer and laboratory technicians, clerical workers, research scientists, volunteers, managers, receptionists and nurses.

Core funding for the Avon Longitudinal Study of Parents and Children (ALSPAC) is provided by the UK Medical Research Council and Wellcome (217065/Z/19/Z) and the University of Bristol. A comprehensive list of grants funding is available on the ALSPAC website (http://www.bristol.ac.uk/alspac/external/documents/grant-acknowledgements.pdf). DAL and AK work in a unit that is supported by the University of Bristol and UK Medical Research Council (MC_UU_00011/6) and DAL holds a European Research Council Advanced Grant (ERC grant agreement no 669545) and is a NIHR Senior Investigator (NF-0616-10102). The funders had no role in the design of the study, the collection, analysis, or interpretation of the data; the writing of the manuscript, or the decision to submit the manuscript for publication. The views expressed in this paper are those of the authors and not necessarily those of any funder.

Amsterdam Born Children and their Development Study (ABCD)

We are grateful to all participating hospitals, obstetric clinics, and general practitioners for their assistance in implementing the ABCD study and thank all of the women who participated for their cooperation. Core funding of the ABCD-study is provided by the Academic Medical Centre, Amsterdam, the Public Health Services, Amsterdam, and the Dutch Organization for Health Research and Development (ZonMw).

Babies After SCOPE: Evaluating the Longitudinal Impact on Neurological and Nutritional Endpoints (BASELINE)

The authors thank the families for their continued support and the Cork BASELINE Birth Cohort Study research team. SCOPE Ireland was supported by the Health Research Board, Ireland (CSA 2007/2). The BASELINE cohort was funded by the National Children's Research Centre, Dublin, Ireland, and the Food Standards Agency of the United Kingdom (grant no. TO7060).

Barwon Infant Study (BIS)

We thank the BIS participants for the generous contribution they have made to this project. We also thank current and past staff for their efforts in recruiting and maintaining the cohort and in obtaining and processing the data and biospecimens.

The establishment work and infrastructure for the BIS was provided by the Murdoch Children's Research Institute, Deakin University and Barwon Health. Subsequent funding was secured from the National Health and Medical Research Council of Australia, The Jack Brockhoff Foundation, the Scobie Trust, the Shane O'Brien Memorial Asthma Foundation, the Our Women's Our Children's Fund Raising Committee Barwon Health, The Shepherd Foundation, the Rotary Club of Geelong, the Ilhan Food Allergy Foundation, GMHBA Limited and the Percy Baxter Charitable Trust, Perpetual Trustees. In-kind support was provided by the Cotton On Foundation and CreativeForce. Research at Murdoch Children's Research Institute is supported by the Victorian Government's Operational Infrastructure Support Program. This work was also supported by NHMRC Senior Research Fellowships (1064629 to DB; 1045161 to RS) and NHMRC Investigator Grants to DB (1175744).

© 2022 Elhakeem A et al. *JAMA Network Open.*

Born in Guangzhou Cohort Study (BIGCS)

We are grateful to the pregnant women who participated in the BIGCS and all obstetric care providers who assisted in the implementation of the study. This work was supported the Department of Science and Technology of Guangdong Province, China (no. 2020B1111170001).

Clinical review of the Health of 22–33 years old conceived with and without ART (CHART)

We would like to acknowledge the participants who generously gave their time to the study and the invaluable contribution of Ms. Jane Koleff to the development of the protocol and in the training of all assessors to undertake the clinical assessments.

The CHART study was supported by the Victorian State Government Operational Infrastructure Support and the Australian Government NHMRC IRIISS awarded to the Murdoch Children's Research Institute, and funded by a National Health & Medical Research Council Project Grant (APP1099641; 2016–2017), Royal Children's Hospital Research Foundation, Monash IVF Research and Education Foundation, and Reproductive Biology Unit Sperm Fund, Melbourne IVF

Danish National Birth Cohort (DNBC)

The authors would like to thank the participants, the first Principal Investigator of DNBC Prof. Jørn Olsen, the scientific managerial team, and DNBC secretariat for being, establishing, developing and consolidating the Danish National Birth Cohort.

The Danish National Birth Cohort was established with a significant grant from the Danish National Research Foundation. Additional support was obtained from the Danish Regional Committees, the Pharmacy Foundation, the Egmont Foundation, the March of Dimes Birth Defects Foundation, the Health Foundation and other minor grants. The DNBC Biobank has been supported by the Novo Nordisk Foundation and the Lundbeck Foundation. Follow-up of mothers and children have been supported by the Danish Medical Research Council (SSVF 0646, 271-08-0839/06-066023, O602-01042B, 0602-02738B), the Lundbeck Foundation (195/04, R100-A9193), The Innovation Fund Denmark 0603-00294B (09-067124), the Nordea Foundation (02-2013-2014), Aarhus Ideas (AU R9-A959-13-S804), University of Copenhagen Strategic Grant (IFSV 2012), and the Danish Council for Independent Research (DFF – 4183-00594 and DFF - 4183-00152).

Etude de cohorte généraliste, menée en France sur les Déterminants pré et post natals précoces du développement psychomoteur et de la santé de l'Enfant (EDEN)

The authors thank the cohort participants and the EDEN mother-child study group, whose members are: I. Annesi-Maesano, J.Y. Bernard, J. Botton, M.A. Charles, P. Dargent-Molina, B. de Lauzon-Guillain, P. Ducimetière, M. de Agostini, B. Foliguet, A. Forhan, X. Fritel, A. Germa, V. Goua, R. Hankard, B. Heude, M. Kaminski, B. Larroque†, N. Lelong, J. Lepeule, G. Magnin, L. Marchand, C. Nabet, F Pierre, R. Slama, M.J. Saurel-Cubizolles, M. Schweitzer, O. Thiebaugeorges.

The EDEN study was supported by Foundation for medical research (FRM), National Agency for Research (ANR), National Institute for Research in Public health (IRESP: TGIR cohorte santé 2008 program), French Ministry of Health (DGS), French Ministry of Research, INSERM Bone and Joint Diseases National Research (PRO-A) and Human Nutrition National Research Programs, Paris-Sud University, Nestlé, French National Institute for Population Health Surveillance (InVS), French National Institute for Health Education (INPES), the European Union FP7 programmes (FP7/2007- 2013, HELIX, ESCAPE, ENRIECO, Medall projects), Diabetes National Research Program (through a collaboration with the French Association of Diabetic Patients (AFD)), French Agency for Environmental Health Safety (now ANSES), Mutuelle Générale de l'Education Nationale a complementary health insurance (MGEN), French national agency for food security, French speaking association for the study of diabetes and metabolism (ALFEDIAM).

Etude Longitudinale Franc¸aise depuis l'Enfance (ELFE)

The authors are grateful to 1) the former members of the Elfe unit without whom the project would never have started: Henri Léridon, initiator and former Principal Investigator of the project, Stéphanie Vandentorren, Claudine Pirus, and Ando Rakotonirina; 2) the expertise and assistance of members of the unit for support

functions, 3) all the researchers who contribute to the projects as members of the Elfe thematic groups and especially their coordinators; 4) all the field research assistants and interviewers; 5) and above all, all the Elfe families who have placed their confidence in us and given up their time to the study.

The Elfe cohort received funding from the National Research Agency Investment for the Future program [ANR-11-EQPX-0038]; French National Institute for Research in Public Health (IRESP TGIR 2009-2001 program); Ministry of Higher Education and Research; Ministry of Environment; Ministry of Health; French Agency for Public Health; Ministry of Culture; and National Family Allowance Fund.

EU Childhood Obesity Project (CHOP)

The authors would particularly like to thank all the cohort participants for their generous collaboration. Furthermore, thanks to all persons who designed and conducted the study, entered the data, and participated in the data analysis and who are represented by the European Childhood Obesity Trial Study Group participants: B Koletzko, V Grote, M Totzauer, K Gürlich, P Schwarzfischer, N Aumüller, V Luque, M Zaragoza-Jordana, N Ferré, J Escribano, R Closa-Monasterolo, A Xhonneux, Jean-Paul Langhendries, E Verduci, E Riva, D Gruszfeld.

The CHOP study has been carried out with partial financial support from the Commission of the European Community, specific RTD Programme "Quality of Life and Management of Living Resources", within the Fifth Framework Program (research grants no. QLRT-2001-00389 and QLK1-CT-200230582), the Sixth Framework Program (contract no. 007036), and Seventh Framework Programme (EarlyNutrition; grant agreement no. 289346), the EU H2020 project LIFECYCLE under grant no. 733206 and the European Research Council Advanced Grant META-GROWTH (ERC-2012-AdG – no.322605) and with financial support from Polish Ministry of Science and Higher Education (2571/7.PR/2012/2). This manuscript does not necessarily reflect the views of the Commission and in no way anticipates the future policy in this area. No funding bodies had any role in the study design, data collection and analysis.

Generation R (Gen R)

The authors gratefully acknowledge the contribution of participants, research collaborators, general practitioners, hospitals, midwives, and pharmacies in Rotterdam.

The general design of the Generation R Study is made possible by financial support from the Erasmus MC, University Medical Center, Rotterdam, Erasmus University Rotterdam, Netherlands Organization for Health Research and Development (ZonMw), Netherlands Organisation for Scientific Research (NWO), Ministry of Health, Welfare and Sport and Ministry of Youth and Families. This project received funding from the European Union's Horizon 2020 research and innovation programme (LIFECYCLE, grant agreement No 733206, 2016, European Joint Programming Initiative “A Healthy Diet for a Healthy Life” (JPI HDHL, EndObesity project, ZonMW the Netherlands no. 529051026). RG received funding of the Dutch Heart Foundation (grant number 2017T013), the Dutch Diabetes Foundation (grant number 2017.81.002), and the Netherlands Organization for Health Research and Development (NWO, ZonMW, grant number 543003109). The study sponsors had no role in the study design, data analysis, interpretation of data, or writing of this report.

Generation XXI (G2I)

G21 was funded by Programa Operacional de Saúde – Saúde XXI, Quadro Comunitário de Apoio III and Administração Regional de Saúde Norte (Regional Department of Ministry of Health) and by Foundation for Science and Technology – FCT (UIDB/04750/2020 - Unidade de Investigação em Epidemiologia (EPIUnit), Instituto de Saúde Pública da Universidade do Porto). The funders had no role in study design, data collection and analysis, interpretation of data, or writing of this report.

Growing Up in Ireland Infant Cohort (GUI)

The authors sincerely thank the thousands of Irish families that contribute to the Growing Up in Ireland Project. None of this would be possible without your time and effort.

Growing Up in Ireland (GUI) is funded by the Department of Children, Equality, Disability, Integration and Youth (DCEDIY). It is being carried out by a consortium of researchers led by the Economic and Social Research Institute (ESRI) and Trinity College Dublin (TCD). GUI is managed by DCEDIY in association with

the Central Statistics Office (CSO). Results in this report are based on analyses of data from Research Microdata Files provided by the Central Statistics Office (CSO). Neither the CSO nor DCEDIY take any responsibility for the views expressed or the outputs generated from these analyses.

Growing Up in New Zealand (GUiNZ)

We thank the participating families of the *Growing Up in New Zealand* cohort study who have given their time and shared the information that allowed us to conduct this research. The *Growing Up in New Zealand* study has been funded by the New Zealand Ministries of Social Development, Health, Education and Justice; the former Ministry of Science Innovation and the former Department of Labour (now both part of the Ministry of Business, Innovation and Employment); the former Ministry of Pacific Island Affairs (now the Ministry for Pacific Peoples); the former Ministry of Women's Affairs (now the Ministry for Women); the Department of Corrections; the Families Commission and the former Social Policy Evaluation and Research Unit; Te Puni Kokiri; New Zealand Police; Sport New Zealand; Housing New Zealand Corporation; and the former Mental Health Commission (now part of the Office of the Health and Disability Commissioner); The University of Auckland and Auckland UniServices Limited. Other support for the study has been provided by the Health Research Council of New Zealand, Statistics New Zealand, the Office of the Children's Commissioner and the Office of Ethnic Affairs (now the Office of Ethnic Communities).

Growing up in Singapore Towards healthy Outcomes (GUSTO)

We thank the GUSTO study group and all clinical and home-visit staff involved. The voluntary participation of all participants is greatly appreciated.

The GUSTO study group includes. Allan Sheppard,Amutha Chinnadurai, Anne Ferguson-Smith, Anne Eng Neo Goh, Arijit Biswas, Audrey Chia, Birit Leutscher-Broekman, Borys Shuter, Shirong Cai, Cheryl Ngo, Chai Kiat Chng, Shang Chee Chong, Christiani Jeyakumar Henry, Mei Chien Chua, Cornelia Yin Ing Chee, Yam Thiam Daniel Goh, Dennis Bier, Chun Ming Ding, Doris Fok, Eric Andrew Finkelstein, Fabian Kok Peng Yap, George Seow Heong Yeo, Wee Meng Han, Helen Chen, Hugo P S Van Bever, Hazel Inskip, Iliana Magiati, Inez Bik Yun Wong, Jeevesh Kapur, Jenny L Richmond, Jerry Kok Yen Chan, Joshua J Gooley, Krishnamoorthy Niduvaje, Bee Wah Lee, Yung Seng Lee, Leher Singh, Sok Bee Lim, Lourdes Mary Daniel, Seong Feei Loh, Yen-Ling Low, Pei-Chi Lynette Shek, Marielle Fortier, Mark Hanson, Mary Foong-Fong Chong, Michael Meaney, Susan Morton, Wei Wei Pang, Pratibha Agarwal, Anqi Qiu, Boon Long Quah, Rob M van Dam, David Stringer, Salome Antonette Rebello, Wing Chee So, Chin-Ying Hsu, Lin Lin Su, Jenny Tang, Kok Hian Tan, Soek Hui Tan, Oon Hoe Teoh, Victor Samuel Rajadurai, PC Wong and Sudhakar K Venkatesh

Healthy Growth Study (HGS)

The HGS was co-funded by the European Union (European Social Fund – ESF) and Greek national funds through the Operational Program "Education and Lifelong Learning" of the National Strategic Reference Framework (NSRF) - Research Funding Program: Heracleitus II. Investing in knowledge society through the European Social Fund.

Italian Twin register (ITR)

The authors thank all twins enrolled in the ITR for study participation. ITR has been funded by the Italian Ministry of Health (MINSAN) (D.L. 502/92, finalised research and by the GenomEUtwin Project (European Union Contract no. QLG2-CT-2002-01254)

Millenium Cohort Study (MCS)

We are grateful to the Centre for Longitudinal Studies (CLS), UCL Social Research Institute, for the use of these data and to the UK Data Service for making them available, and to the children and families who take part in the study. Neither CLS nor the UK Data Service bear any responsibility for the analysis or interpretation of these data.

Multiple Birth Cohort Study (MUBICOS)

The authors thank all MUBICOS families for participation. MUBICOS was partly funded by Chiesi Onlus Foundation.

© 2022 Elhakeem A et al. *JAMA Network Open.*

Nascita e INFanzia: gli Effetti dell'Ambiente (NINFEA)

The authors thank all families participating in the NINFEA cohort.

The NINFEA cohort was initially funded by the Compagnia SanPaolo Foundation and the Piedmont Region. It received funding from European projects: CHICOS (FP7 grant number HEALTH-FP7-2009-241604, LifeCycle (H2020 grant number 733206), ATHLETE (H2020 grant number 874583).

Norwegian Mother, Father and Child Cohort Study (MoBa)

The authors are grateful to all the participating families in Norway who take part in this on-going cohort study.

The Norwegian Mother, Father and Child Cohort Study is supported by the Norwegian Ministry of Health and Care Services and the Ministry of Education and Research.

The study was partly funded by the Norwegian Research Council's Centres of Excellence Funding Scheme, no 262700. MCM is funded by the European Research Council (ERC) under the European Union's Horizon 2020 research and innovation programme (grant agreement No 947684)

Data from the Norwegian Mother, Father and Child Cohort Study and the Medical Birth Registry of Norway used in this study are managed by the national health register holders in Norway (Norwegian Institute of public health) and can be made available to researchers, provided approval from the Regional Committees for Medical and Health Research Ethics (REC), compliance with the EU General Data Protection Regulation (GDPR) and approval from the data owners. The consent given by the participants does not open for storage of data on an individual level in repositories or journals. Researchers who want access to data sets for replication should apply through helsedata.no. Access to data sets requires approval from The Regional Committee for Medical and Health Research Ethics in Norway and an agreement with MoBa.

Piccolipiù

The authors thank all the families who took part in the study, and the Piccolipiù research group.

The study was funded by the Italian National Centre for Disease Prevention and Control (CCM grant 2010), by the Italian Ministry of Health (art 12 and 12bis Dl .gs. vo 502/92).

Prospective Study on Infancy in Italy (GASPII)

The authors are grateful to all the participating families in Rome who take part in this on-going cohort study, and to all the field workers and interviewers of the project. The study was funded by Italian Ministry of Health.

Southampton Women's Survey (SWS)

The authors are grateful to the women of Southampton who gave their time to take part in the Southampton Women's Survey and to the research nurses and other staff who collected and processed the data.

The SWS is supported by grants from the Medical Research Council, National Institute for Health Research Southampton Biomedical Research Centre, British Heart Foundation, UK Food Standards Agency, British Lung Foundation, Versus Arthritis, University of Southampton and University Hospital Southampton National Health Service Foundation Trust, and the European Union's Seventh Framework Programme (FP7/2007-2013), project Early Nutrition (grant 289346) and from the European Union's Horizon 2020 research and innovation programme (LIFECYCLE, grant agreement No 733206). Study participants were drawn from a cohort study funded by the Medical Research Council and the Dunhill Medical Trust.

The Trøndelag Health Study (HUNT)

The Trøndelag Health Study (HUNT) is a collaboration between HUNT Research Centre (Faculty of Medicine and Health Sciences, NTNU, Norwegian University of Science and Technology), Trøndelag County Council, Central Norway Regional Health Authority, and the Norwegian Institute of Public Health.

© 2022 Elhakeem A et al. *JAMA Network Open.*
